# Complexes of Copper and Iron with Pyridoxamine, Ascorbic Acid, and a Model Amadori Compound: Exploring Pyridoxamine’s Secondary Antioxidant Activity

**DOI:** 10.3390/antiox10020208

**Published:** 2021-02-01

**Authors:** Guillermo García-Díez, Roger Monreal-Corona, Nelaine Mora-Diez

**Affiliations:** Department of Chemistry, Thompson Rivers University, Kamloops, BC V2C 0C8, Canada; garciadiezg18@mytru.ca (G.G.-D.); monrealcoronar18@mytru.ca (R.M.-C.)

**Keywords:** pyridoxamine, ascorbate, Amadori compounds, aminoguanidine, superoxide radical anion, glycation inhibitor, copper complexes, iron complexes, Haber–Weiss cycle, Marcus theory

## Abstract

The thermodynamic stability of 11 complexes of Cu(II) and 26 complexes of Fe(III) is studied, comprising the ligands pyridoxamine (PM), ascorbic acid (ASC), and a model Amadori compound (AMD). In addition, the secondary antioxidant activity of PM is analyzed when chelating both Cu(II) and Fe(III), relative to the rate constant of the first step of the Haber-Weiss cycle, in the presence of the superoxide radical anion $(O_{2}^{•-}$) or ascorbate (ASC^−^). Calculations are performed at the M05(SMD)/6-311+G(d,p) level of theory. The aqueous environment is modeled by making use of the SMD solvation method in all calculations. This level of theory accurately reproduces the experimental data available. When put in perspective with the stability of various complexes of aminoguanidine (AG) (which we have previously studied), the following stability trends can be found for the Cu(II) and Fe(III) complexes, respectively: ASC < AG < AMD < PM and AG < ASC < AMD < PM. The most stable complex of Cu(II) with PM (with two bidentate ligands) presents a $\Delta G_{f}^{0}$ value of −35.8 kcal/mol, whereas the Fe(III) complex with the highest stability (with three bidentate ligands) possesses a $\Delta G_{f}^{0}$ of −58.9 kcal/mol. These complexes can significantly reduce the rate constant of the first step of the Haber-Weiss cycle with both $O_{2}^{•-}$ and ASC^−^. In the case of the copper-containing reaction, the rates are reduced up to 9.70 × 10^3^ and 4.09 × 10^13^ times, respectively. With iron, the rates become 1.78 × 10^3^ and 4.45 × 10^15^ times smaller, respectively. Thus, PM presents significant secondary antioxidant activity since it is able to inhibit the production of ·OH radicals. This work concludes a series of studies on secondary antioxidant activity and allows potentially new glycation inhibitors to be investigated and compared relative to both PM and AG.

## 1. Introduction

Glycation (also known as non-enzymatic glycosylation) is the process by which saccharides found in the bloodstream react with different nucleophiles (DNA, lipids, or proteins) to form compounds, which can pose a hazard to the body. First, a Schiff base intermediate forms due to the attack of the nucleophilic groups to the carbonyl groups present in the sugars. After a series of intramolecular rearrangement reactions, these intermediates become Amadori compounds, which can further react and become advanced glycation end-products (AGEs) [1,2]. While some of these are harmless, others are extremely reactive, and can be the source of different ailments, such as Alzheimer’s disease or eye disease [3,4]. Carbonyl species, radicals, and Cu(II) and Fe(III) ions (which catalyse the autoxidation of Amadori compounds) are known to increase the formation of AGEs, and, thus, scavenging these could halt this damaging process [5].

Several compounds, such as aminoguanidine (AG), pyridoxamine (PM), metformin, LR-74, carnosine, or tenilsetam, have already been tested as potential glycation inhibitors, both experimentally and theoretically [6,7,8,9]. Moreover, AG and PM have been investigated as drugs for diabetic nephropathy (under the name pimagedine and the brand name Pyridorin, respectively) [10,11,12]. From an experimental standpoint, PM is known to inhibit the formation of AGEs, while AG is not capable of doing so [13]. Theoretically, the complexes of AG, PM, ascorbic acid (ASC), and a model Amadori compound (AMD) with Cu(II) and Fe(III) have also been studied [14,15]. Finally, the reaction between various carbonyl species (sugars such as ribose or glucose) and glycation inhibitors (metformin, PM, and its analogues) have been studied experimentally and theoretically [16,17,18,19].

Previous studies of the Cu(II) and Fe(III) complexes with AG, PM, ASC, and AMD were performed at the B3LYP(CPCM)/6-31+G(d), and both the B3LYP(CPCM)/6-31+G(d) and the M06(CPCM)/6-31+G(d,p) levels of theory, respectively [14,15]. In all cases, solvent effects (water) were simulated by making use of the Cosmo Polarizable Continuum Method (CPCM). Nonetheless, pH was not taken into account, even though all the ligands present acid-base properties, and Gibbs free energy changes were reported at the 1 atm reference state relative to the isolated Cu^2+^ and Fe^3+^, the free ligands, and H_2_O species involved in each case. In both publications, the stability order of the complexes studied was reported to be ASC < AG < AMD < PM. Regarding the Cu(II) complexes, the most stable complexes were always square-planar and presented two ligands. On the other hand, the most stable Fe(III) complexes were invariably octahedral, and had three ligands.

Ramis et al. recently studied the free-radical scavenging activity of AG (i.e., its primary antioxidant activity) [20]. The level of theory employed was M05-2X(SMD)/6-311+G(d,p). The thermodynamics and kinetics of the reactions of AG with the ·OCH_3_ and ·OOH radicals were investigated under physiological conditions in polar and non-polar environments. It was found that AG (which is mostly protonated in aqueous solution at physiological pH) is a moderate free-radical scavenger. This exclusively happens via hydrogen-atom transfer (HAT), with a larger rate constant in nonpolar media. Thus, one of the three mechanisms of AG as a glycation inhibitor has been examined. More recently, the primary antioxidant activity of PM was also investigated by making use of the same level of theory [21]. It was found that PM can trap ·OCH_3_ radicals in aqueous and lipidic media via the HAT reaction. The reactive hydrogens being transferred are the ones attached to the protonated pyridine, the protonated amino group, and the phenolic oxygen atom. The reactivity of this ligand toward ·OOH and ·OOCH_3_ is much reduced, but PM does scavenge these species with a moderate rate constant in aqueous media. The authors argue that these properties help to explain the activity of this molecule as a glycation inhibitor.

To study the activity of AG as a chelator of Cu(II) and Fe(III) (another potential mechanism of a glycation inhibitor), our group revisited previous studies done by Ortega-Castro et al. [14,15]. We opted to use the M05(SMD)/6-311+G(d,p) level of theory to be consistent with the research of Ramis et al. [20]. This way, proper comparisons can be made between the different publications, and the potential of AG as a glycation inhibitor can be fully understood. We investigated the thermodynamic stability of thirty Cu(II) complexes and sixty Fe(III) complexes with AG. We found that AG will only form stable complexes if deprotonated, even though this molecule is protonated at physiological pH. Moreover, when comparing coordination compounds with the same number of ligands and type of coordination, the Cu(II) complexes were more stable. Nonetheless, since Fe(III) can coordinate to three AG molecules, whereas Cu(II) can only bond to two, the most stable complex was a 1:3 Fe(III)-AG complex. We hypothesized that, at lower concentrations, AG will tend to coordinate with Cu(II), and only at higher concentrations will the most stable Fe(III) complex form [22,23].

**Figure 1:**
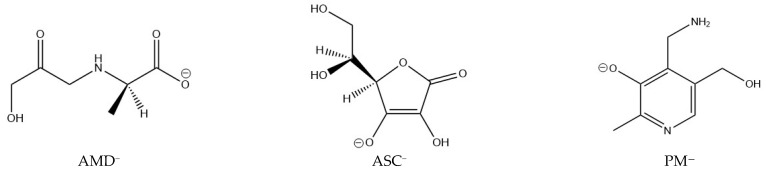
Structures of the ligands studied that form the most stable complexes with Cu(II) and Fe(III) at physiological pH: pyridoxamine (PM), ascorbic acid (ASC), and a model Amadori compound (AMD).

To fully understand the chelating activity of AG, the stability of the complexes this ligand forms has to be put in perspective, as done by previous researchers. AG will prevent the oxidation of Amadori compounds if it can form more thermodynamically stable complexes with Cu(II) and Fe(III) than the Amadori compounds, immobilizing the metal ions. In the present work, the thermodynamic stability of 37 complexes of PM, ASC, and AMD with Cu(II) and Fe(III) at physiological pH is examined and compared. These complexes contain varying numbers of ligands. Deprotonation energies are considered wherever necessary, as AMD is a neutral zwitterion and PM is a cationic zwitterion at physiological pH, but the most stable ligand species in each case are the anionic forms (see Figure 1).

We also study the secondary antioxidant activity of PM. A compound is said to present this activity if it can coordinate to Cu(II) or Fe(III) cations and slow down the rate constant of the first step of the Haber-Weiss cycle, as shown in Equation (1) (focusing on iron). If this process is not hindered, the reduced metal ions can react with hydrogen peroxide, leading to the formation of very reactive ·OH radicals (this second step is known as the Fenton reaction) [24].

Fe^3+^ + O_2_·^−^ → Fe^2+^ + O_2_ (1)

Fe^2+^ + H_2_O_2_ → Fe^3+^ + OH^−^ + ·OH

The reductant in this process can also be the ascorbate anion (ASC^−^) [25,26], since this compound undergoes oxidation in the presence of Cu(II) and Fe(III) [27]. However, it is known experimentally that both AG and PM can slow down this reaction [6,28]. In our previous publications, we showed that both AG and dihydrolipoic acid can slow down the reaction rate of the first step of the Haber–Weiss cycle with both metals when the reducing agent is ASC^−^ [22,23,29]. However, they exhibited no secondary antioxidant activity when the reducing agent is the superoxide radical anion (O_2_·^−^).

## 2. Computational Details

Calculations were performed by means of the Gaussian09 software package, and the structures were fully optimized and characterized at the M05(SMD)/6-311+G(d,p) level of theory [30]. The SMD (solvation model based on density) method was employed to take into account the solvent effects (water) in all calculations, and the ultrafine integration grid was also used [31]. Both M05 and M06 are hybrid meta functionals that accurately model metallic interactions with M06 being an improvement of the M05 functional [32,33]. Nonetheless, we decided to use the M05 over the M06 to be consistent with our previous publications, which all made use of this functional [20,22,23].

All the relevant thermodynamic information (absolute standard Gibbs free energies and enthalpies at 298.15 K) of the species studied can be found in Table S1 of the Supporting Information document. The Cartesian coordinates of the calculated complexes and the structures of relevant ones are also displayed in the Supporting Information. The standard Gibbs free energy of formation ($\Delta G_{f}^{0}$) for each complex was calculated using Equation (2), employing the G^0^ values of the reactants and products. This value refers to the formation of a complex from its infinitely separated ligands and solvated central ion. Consequently, $\Delta G_{f}^{0}$ was used to calculate the formation constant ($K_{f}$), as shown in Equation (3).

(2) $$\Delta G_{f}^{0}=\sum G_{products}^{0}-\sum G_{reactants}^{0}$$

(3) $$K_{f}=e^{-\frac{\Delta G_{f}^{0}}{RT}}$$

Using Equation (4), the rate constant (*k*) was calculated following conventional transition state theory. The standard Gibbs free energy of activation ($\Delta G^{\neq }$) was estimated by applying Marcus theory [34,35]. For rate constants in the diffuse-limited regime (k > 1.0 × 10^8^ M^−1^ s^−1^), the Collins-Kimball theory [36] was applied to determine apparent rate constants (k_app_) in combination with the steady-state Smoluchowski rate constant expression for an irreversible diffusion-controlled bimolecular reaction [37], and the Stokes-Einstein approach for the diffusion coefficients [38,39]. Details on the expressions applied are provided in Appendix 1 of the Supporting Information.

(4) $$k=\frac{k_{B}T}{h}e^{-\frac{\Delta G^{\neq }}{RT}}$$

Most of the complexes studied throughout this paper contain a metal centre with unpaired electrons. Because of this, it is crucial to examine whether these present any spin contamination before or after annihilation. This effect arises due to the merging of different electronic spin states, and it may have an effect on the energies and/or geometries calculated [40]. Table S2 shows the $\langle {\hat{S}}^{2}\rangle$ values of all the Cu(II), Fe(III), and Fe(II) complexes before and after annihilation. It is expected that species with two, four, and five unpaired electrons present $\langle {\hat{S}}^{2}\rangle$ values of 0.75, 6.00, and 8.75, respectively, when there is no spin contamination. As observed, spin contamination is negligible after spin annihilation in all the species considered.

## 3. Results and Discussion

### 3.1. Considerations Taken When Working with the AMD, ASC, and PM Ligands

To put into perspective the results of our previous publications and to effectively compare the stability of the AG complexes with other complexes of relevance [22,23], we decided to optimize several Cu(II) and Fe(III) complexes with AMD, ASC, and PM, following an equivalent methodological strategy as in the previously mentioned papers. Eleven complexes were calculated with Cu(II), and, of the 26 complexes optimized with Fe(III), 12 present an octahedral environment and 14 exhibit lower coordination numbers.

The structures of the deprotonated ligands (AMD, ASC, and PM), which form the most stable complexes at physiological pH, are shown in Figure 1. Concerning stereochemistry, the L isomer of ascorbic acid was used [41], as this is the naturally occurring isomer (it was used in a different stereochemistry in previous studies [14,15]). In the case of the Amadori compound model, we decided to employ the R isomer. PM presents no stereochemistry.

Different coordination sites were explored for the different ligands. In the case of AMD, three different sets of bidentate coordination points with Cu(II) were explored: the alcohol and ketone groups, the ketone and the amine groups, and the amine and the carboxylate groups. Another possibility for AMD is to chelate in a tridentate fashion, via the carboxylate, the amine, and the ketone groups. These complexes showed high stability. However, in agreement with several experimental studies [42,43], coordination via the carboxylate and the amine groups only led to the most stable complexes by a wide margin, and, thus, these coordination points were explored for the remaining bidentate complexes with AMD.

Regarding ASC, bidentate complexes in which this ligand would coordinate via the deprotonated hydroxyl group and the hydroxyl group vicinal to it, as done by Ortega-Castro et al. [14,15], were attempted. Nevertheless, in all cases studied, the ligand lost one coordination while keeping the coordination through the deprotonated site, becoming monodentate. Some of the Cu(II) and Fe(III) complexes with ASC, which lost the second coordination site, showed an unusually low coordination number (unknown from experimental data) and were discarded. Their structures and thermodynamic values are reported in Figure S1 and Table S3 of the Supporting Information. Finally, PM was invariably used as a bidentate ligand, coordinating through the amine and the deprotonated phenol group.

Initially, Equations (5)–(10) were used to calculate the Gibbs free energy of formation of the complexes.

*x*AMD^−^ + [Cu(H_2_O)_4_]^2+^ ⇆ [Cu(AMD)_x_(H_2_O)_n_]^(2−x)+^ + (4 − n)H_2_O (5)

*x*ASC^−^ + [Cu(H_2_O)_4_]^2+^ ⇆ [Cu(ASC)_x_(H_2_O)_n_]^(2−x)+^ + (4 − n)H_2_O (6)

*x*PM^−^ + [Cu(H_2_O)_4_]^2+^ ⇆ [Cu(PM)_x_(H_2_O)_n_]^(2−x)+^ + (4 − n)H_2_O (7)

*x*AMD^−^ + [Fe(H_2_O)_6_]^3+^ ⇆ [Fe(AMD)_x_(H_2_O)_n_]^(3−x)+^ + (6 − n)H_2_O (8)

*x*ASC^−^ + [Fe(H_2_O)_6_]^3+^ ⇆ [Fe(ASC)_x_(H_2_O)_n_]^(3−x)+^ + (6 − n)H_2_O (9)

*x*PM^−^ + [Fe(H_2_O)_6_]^3+^ ⇆ [Fe(PM)_x_(H_2_O)_n_]^(3−x)+^ + (6 − n)H_2_O (10)

To maintain an equivalent number of reactant and product species, clusters with x ligands and (4 − n) or (6 − n) water molecules were calculated. Their thermodynamic data and structures are reported in the Supporting Information along with their Cartesian coordinates (see the species labelled **{F3}** to **{F17}**). This was also the methodology followed in previous publications [22,23,29,44].

Nonetheless, given that the ligands studied possess acid-base properties, deprotonation energies ($\Delta G_{dep}$) were taken into account when studying the stability of the various complexes. In the case of ASC, this was not necessary since it is already deprotonated at physiological pH (pK_a_ = 4.1) [45]. In the case of AMD and PM, different approaches were carried out in order to estimate this deprotonation energy, which is a task for which a pK value is required. The model Amadori compound would be a neutral zwitterion at physiological pH, as it possesses an amine and a carboxylic group. Following the approach described by Brown and Mora-Diez, we were able to calculate the pK_a_ of the (neutral) model Amadori compound [46]. More information can be found in Appendix 2 of the Supporting Information.

On the other hand, PM is also protonated at physiological pH. In addition to this, the phenolic proton migrates to one of the two nitrogen atoms. Thus, PM is a protonated zwitterion in these conditions. However, the species that forms the most stable Cu(II) and Fe(III) complexes is the anion displayed in Figure 1. The Gibbs free energy ($\Delta G_{dep}$) cost to doubly-deprotonate the most stable form at physiological pH was found by means of the pK_a_ values reported by Casasnovas et al. [9]. Each possible conformer of PM was optimized in order to find the most stable ones. Please refer to Appendix 3 in the Supporting Information for a more in-depth explanation of the procedure followed.

The deprotonation equilibrium of AMD is displayed in Equation (11). The set of equations employed to find the deprotonation energy of the model Amadori compound is shown in Equations (12)–(15). The approach would be identical for PM, but two deprotonations would occur instead of one and Equation (16) would result. The $\Delta G_{dep}$ values for AMD and PM were calculated to be 2.2 and 7.2 kcal/mol, respectively, at physiological pH and 298.15 K. We should expect some level of error associated with the value calculated for AMD, which, in itself, is an approximation for an Amadori compound.

(11) $$AMD\;⇆\;AMD^{-}+\;H^{+}$$

(12) $$pK=-logK=-log\frac{\left[AMD^{-}\right]\left[H^{+}\right]}{\left[AMD\right]}=\frac{\Delta G^{0}}{RTln10}$$

(13) $$K^{\prime }=\frac{K}{\left[H^{+}\right]}=\frac{\left[AMD^{-}\right]}{\left[AMD\right]}=\frac{e^{\frac{-\Delta G^{0}}{RT}}}{\left(10^{-pH}\right)}=e^{\frac{-\Delta G^{\prime }}{RT}}$$

(14) $$\Delta G^{\prime }=\Delta G^{0}-\;pHRTln10$$

(15) $$\Delta G_{dep}=\left(pK-pH\right)RTln10$$

(16) $$\Delta G_{dep}=\left(pK-2pH\right)RTln10$$

This deprotonation energy was subtracted from the Gibbs free energy of formation of the complexes (taking into account the number of ligands present in the coordination compounds), leading to the standard Gibbs free energy change ($\Delta G_{f}^{{}^{\circ}}$) displayed in Table 1, Table 2 and Table 3. The actual equilibria used to study the thermodynamics of the formation of the AMD^−^ and PM-containing complexes were as follows:

(17) $$x\mathrm{AMD}\;+\;[\mathrm{Cu}{\left(H_{2}O\right)}_{4}]{}^{2+}\;⇆\;{\left[\mathrm{Cu}{\left(\mathrm{AMD}\right)}_{x}{\left(H_{2}O\right)}_{n}\right]}^{(2-x)+}\;+\;(4\;-\;n)H_{2}O\;+\;xH^{+}$$

(18) $$x{\mathrm{PM}}^{+}\;+\;{\left[\mathrm{Cu}{\left(H_{2}O\right)}_{4}\right]}^{2+}\;⇆\;{\left[\mathrm{Cu}(\mathrm{PM}{)}_{x}(H_{2}O{)}_{n}\right]}^{(2-x)+}\;+\;(4\;-\;n)H_{2}O\;+\;2xH^{+}$$

(19) $$x\mathrm{AMD}\;+\;{\left[\mathrm{Fe}{\left(H_{2}O\right)}_{6}\right]}^{3+}\;⇆\;{\left[\mathrm{Fe}(\mathrm{AMD}{)}_{x}(H_{2}O{)}_{n}\right]}^{(3-x)+}\;+\;(6\;-\;n)H_{2}O\;+\;xH^{+}$$

(20) $$x{\mathrm{PM}}^{+}\;+\;{\left[\mathrm{Fe}{\left(H_{2}O\right)}_{6}\right]}^{3+}\;⇆\;{\left[\mathrm{Fe}(\mathrm{PM}{)}_{x}(H_{2}O{)}_{n}\right]}^{(3-x)+}\;+\;(6\;-\;n)H_{2}O\;+\;2xH^{+}$$

### 3.2. Complexes of Cu(II) with AMD, ASC, and PM

**Figure 2:**
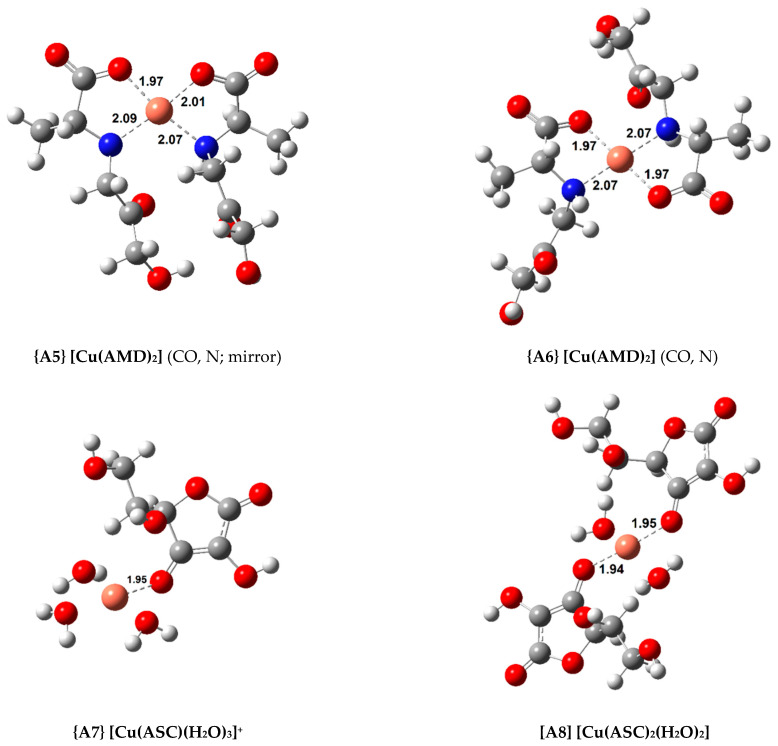

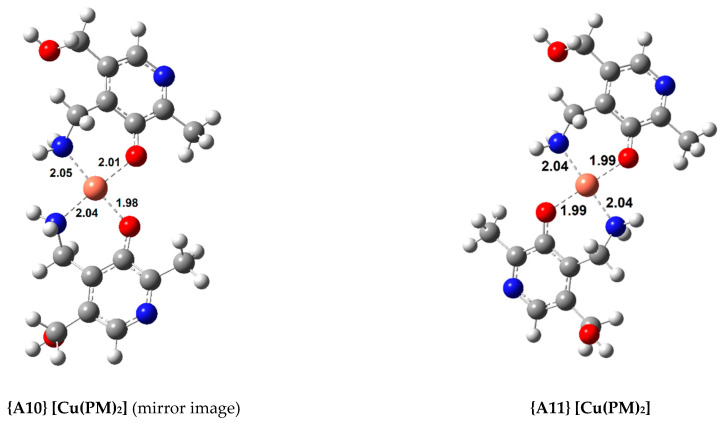
Optimized geometries of the most stable Cu(II) complexes with AMD^−^, ASC^−^, and PM^−^ (bond distances in Å).

In total, 11 complexes were calculated for Cu(II) with a square-planar geometry (with a coordination number four). Their standard formation Gibbs free energy change ($\Delta G_{f}^{{}^{\circ}}$) and formation constant ($K_{f}$, $logK_{f}$) are shown in Table 1. Figure 2 displays the structures of the most relevant complexes with AMD, ASC, and PM, respectively.

As explained earlier, four 1:1 AMD complexes were attempted, including three bidentate (complexes **{A1}**, **{A2}** and **{A3}**) and a tridentate one, complex **{A4}**. Complex **{A3}**, in which AMD coordinates via the carboxylate (CO) and the amine (N) groups, proved to be the most stable bidentate complex ($\Delta G_{f}^{{}^{\circ}}$ = −19.7 kcal/mol). However, if a third coordination point is added (the ketone, k), the stability of the complex slightly increases (complex **{A4}**, $\Delta G_{f}^{{}^{\circ}}$ = −20.7 kcal/mol). Introducing a second AMD ligand further raises the stability of the complexes. Two 1:2 complexes were optimized. One of them contains a plane of symmetry (complex **{A5}**, labeled “mirror”), whereas the other does not (complex **{A6}**). This difference can be easily seen in Figure 2. A similar approach was taken in our previous publications with AG, and showed that sometimes the “mirror” complexes were more stable than the asymmetrical ones [22,23]. Nevertheless, in this case, the symmetrical complexes displayed a lower stability by almost 1.5 kcal/mol. The most stable Cu(II) complex with AMD is complex **{A6}** ($\Delta G_{f}^{{}^{\circ}}$ = −35.3 kcal/mol).

Two ASC complexes, complexes **{A7}** and **{A8}**, were calculated. As expected, adding a second ASC ligand to complex **{A8}** increased stability ($\Delta G_{f}^{{}^{\circ}}$ = −13.7 kcal/mol), but this was the least stable group of complexes studied.

Finally, three complexes were optimized with PM and Cu(II), a 1:1 complex, **{A9}**, and two 1:2 complexes (**{A10}** and **{A11}**, displayed in Figure 2). Once again, both the symmetrical and asymmetrical complexes were optimized. As expected, the 1:2 complexes are more stable than the 1:1 complex (almost twice as stable), with the asymmetrical one being more so, **{A11}** ($\Delta G_{f}^{{}^{\circ}}$ = −35.8 kcal/mol). The most stable Cu(II) complex with AMD is almost as stable as the most stable PM complex. The experimental formation constants ($logK_{f}$) of complexes **{A9}** and **{A11}** have been reported as 10.80 and 25.46 (from different groups), respectively [47,48]. These values were derived from the solvated ions in solutions of varying pH (ranging from 2.5 to 7.0). Previous calculations report formation constants for **{A9}** and **{A11}** of 13.44/12.53 and 24.98/22.24, respectively [9]. This group followed a different procedure from ours, using the complexes of Cu(II) with glycine and β-alanine as references. Casasnovas et al. also investigated the pK_a_ values and protonations of these complexes [9]. They found that complex **{A11}** is even more stable when the pyridine nitrogens are protonated. Nonetheless, the experimental pK_a_ value for these nitrogens are 6.4 and 7.1, and they showed that, at physiological pH, these will be mostly deprotonated, and **{A11}** will be one of the major species, if not the most abundant. Our formation constants for **{A9}** and **{A11}** are 14.22 and 26.23, respectively, which is in good agreement with the previous values (especially with the experimental value of the 1:2 complex, which is much more stable). This agreement validates our methodology.

When the stability of these groups of complexes is compared with the most stable Cu(II) complexes with AG studied in our previous publication (their calculated thermodynamic data is shown in Table 1) [22], interesting observations can be made. The least stable Cu(II) complexes are with ASC, followed by those of AG, in agreement with experimental results [6,28]. The most stable Cu(II) complex with AG is a bidentate 1:2 complex with $\Delta G_{f}^{{}^{\circ}}$ = −29.7 kcal/mol. If both AG and ASC are present, AG would coordinate Cu(II) preferentially, protecting ASC from being oxidized by these ions. Moreover, as we found in our previous paper, the Cu(II) complexes with AG will also slow down the first step of the Haber-Weiss cycle when the reductant is ascorbate [22]. Actually, the most stable complex is capable of reducing the rate constant (when comparing to the hydrated Cu(II) ion) by a factor of 10^7^.

At the other end of the spectrum lie the complexes with AMD and PM, with similar stability (see complexes **{A6}** and **{A11}**). Thus, the overall stability order is ASC < AG < AMD < PM. These results are in agreement with the experimental data because it is known that AG is capable of preventing the oxidation of ascorbic acid, but not of Amadori compounds [13]. The AG complexes are much less stable than those of AMD, and, thus, the AG ligand cannot protect AMD from oxidation with Cu(II). PM, however, can hinder both oxidations (ASC and AMD) because it forms more stable Cu(II) complexes than ascorbic acid and it can compete with the Amadori compounds, which partially chelate this ion and prevent it from oxidizing AMD [13].

The previous study of Cu(II) complexes with ASC, PM, and AMD was done at the B3LYP(CPCM)/6-31+G(d) level of theory [14]. These results differ significantly from ours mainly due to the methodology followed to calculate the $\Delta G_{f}^{{}^{\circ}}$ values. The previous study reports $\Delta G_{f}^{{}^{\circ}}$ values that do not include converting to the 1 M reference state and do not take pH into account, among other important details [14]. Regarding the PM complexes, this group optimized two square-planar and two octahedral complexes. We have not been able to optimize an octahedral Cu(II) complex so far, as this metal ion prefers square-planar geometry. Nonetheless, their square-planar complexes match our complexes **{A9}** and **{A11}**. The $\Delta G_{f}^{{}^{\circ}}$ values they report for these complexes in solution are −143.4 and −177.2 kcal/mol, respectively. Our values are −19.4 and −35.8 kcal/mol, respectively. Concerning the AMD complexes, Ortega-Castro et al. optimized only two complexes, equivalent to complexes **{A4}** and **{A6}** in this paper. Once again, the stability values reported greatly differ from ours. $\Delta G_{f}^{{}^{\circ}}$ values were calculated as −137.4 and −158.1 kcal/mol, respectively, while we found values of −20.7 and −35.3 kcal/mol (very similar to those of the PM complexes). Finally, two ASC complexes were calculated by Ortega-Castro et al. [14]. We attempted to model these compounds, but it was not possible to optimize their geometries at the level of theory applied, as the ASC ligand showed a marked preference to be monodentate. As can be seen, not only are the calculated $\Delta G_{f}^{{}^{\circ}}$ values extremely different, but the stability trends that do not match either (**{A11}** should be more stable than **{A6}** by 19.1 kcal/mol, according to their results). These discrepancies can be explained by different factors. First and foremost, the level of theory employed is totally different from ours. In addition to this, they calculated the $\Delta G_{f}^{{}^{\circ}}$ of the different complexes by subtracting from the standard Gibbs free energy of the complexes and the standard Gibbs free energy of the isolated Cu^2+^, ligands, and H_2_O species, without making reference state conversions. Finally, the deprotonation energy of the various ligands was not taken into account, and the stereochemistry of the ligands used was inconsistent (the isomer of ASC was not the naturally occurring one, and it appears that different isomers of AMD were employed without any apparent rationale).

### 3.3. Complexes of Fe(III) with AMD, ASC, and PM

**Figure 3:**
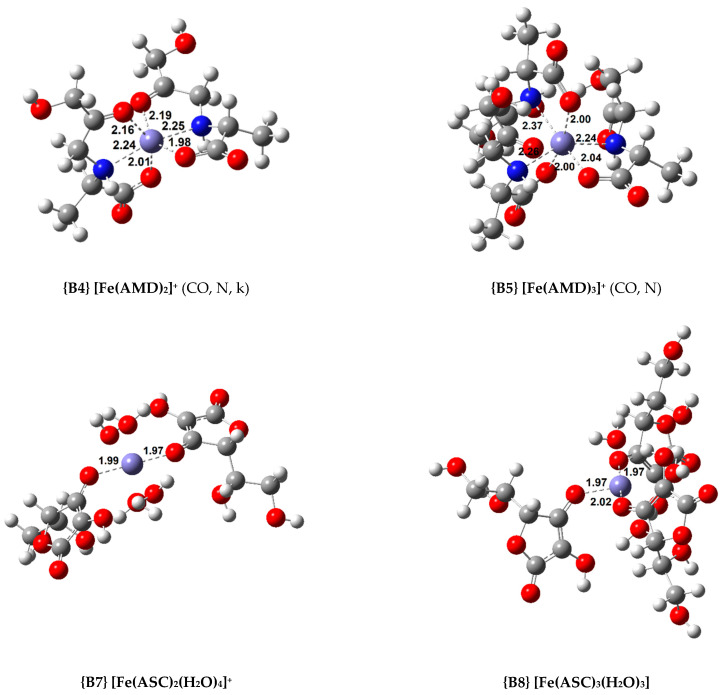

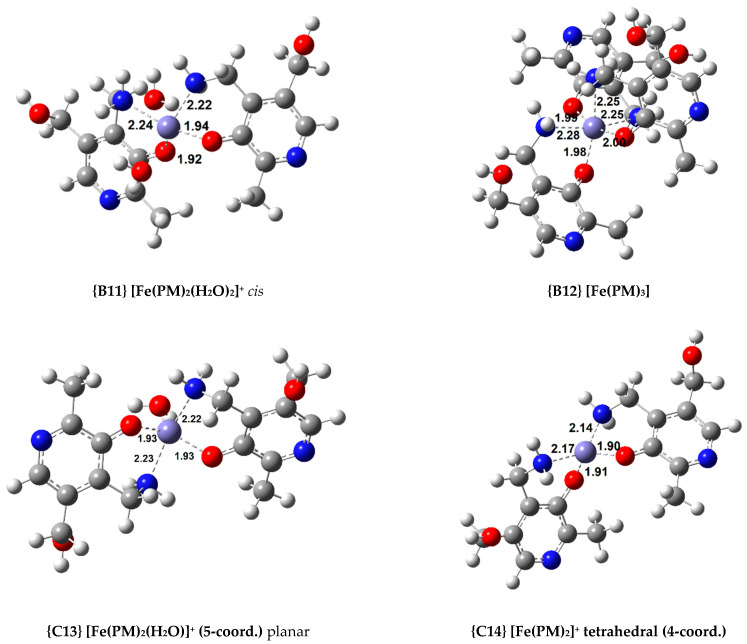
Optimized geometries of the most relevant Fe(III) complexes with AMD^−^, ASC^−^, and PM^−^ (bond distances in Å).

Twelve octahedral Fe(III) complexes were optimized and 14 complexes with lower coordination numbers were also calculated. All the calculated iron complexes are high spin [23]. Their standard Gibbs free energy of formation and formation constant are displayed in Table 2 and Table 3 for the octahedral and non-octahedral complexes, respectively. Figure 3 shows the structures of the most relevant Fe(III) complexes with AMD, ASC, and PM, respectively.

Regarding the octahedral complexes, a trend similar to the Cu(II) coordination compounds can be found. The more ligands added, the more stable the complex. In the case of the AMD complexes, a tridentate ligand confers added stability to the system (compare complexes **{B1}** and **{B2}**, and **{B3}** and **{B4}**). Nonetheless, the most stable complex of this group is the 1:3, where three bidentate AMD ligands chelate the iron centre, complex **{B5}** ($\Delta G_{f}^{{}^{\circ}}$ = −48.9 kcal/mol).

As stated previously, we attempted to model ASC complexes where the ligand would chelate in a bidentate fashion. However, we did not succeed at this, and all the complexes containing ASC are monodentate. As expected, the stability of the coordination compounds increases as the number of ligands grows. Nevertheless, these complexes are less stable than the complexes with AMD. The most stable Fe(III) complex with ASC is **{B8}** ($\Delta G_{f}^{{}^{\circ}}$ = −46.9 kcal/mol).

Finally, four Fe(III) complexes with PM were optimized, and these are among the most stable complexes calculated. For the 1:2 complexes, two isomers (*cis* and *trans*) were optimized (complexes **{B10}** and **{B11}**, the structure of complex **{B11}** can be seen in Figure 3). The *cis* isomer proved to be the more stable by 3.7 kcal/mol. Nonetheless, the 1:3 complex, **{B12}** ($\Delta G_{f}^{{}^{\circ}}$ = −58.9 kcal/mol), is the most stable by a wide margin.

On the other hand, when non-octahedral complexes are studied, interesting trends are observed. Among the AMD compounds, the 1:1 non-octahedral complexes are more stable than the octahedral ones (compare **{B2}** with **{C4}**). Conversely, the 1:2 octahedral coordination compounds show a greater stability than the analogous complexes with a lower coordination number (**{B4}** is more stable than **{C6}**). The ASC complexes we obtained show the same stability trends as the AMD ones. Complex **{C8}** is more stable than **{B6}**, but complex **{B7}** is the most stable of all the 1:2 complexes, more so than the complexes with a coordination number lower than 6. Regarding the PM complexes, a reversal in stability can be observed. The most stable 1:1 complex is octahedral (complex **{B9}**), but the most stable 1:2 complex is **{C14}**, which is a tetrahedral coordination compound. Finally, all the octahedral 1:3 complexes are more stable than the rest of the complexes in each group. Moreover, in the case of AMD and PM, no other possible geometries can be modelled.

The trend in stability of the different sets of Fe(III) complexes differs from that of the Cu(II) ones. Whereas, in the Cu(II) complexes, we found the stability order of ASC < AG < AMD < PM, in the Fe(III) complexes, the stability trend is: AG < ASC < AMD < PM. Nevertheless, this is not in total disagreement with the experimental data. As previously stated, PM is known to inhibit the oxidation of both ASC and AMD by metal ions. As shown in Table 2, the complexes that this molecule forms with Fe(III) are much more stable than the rest, thus, explaining this fact (complex **{B12}** has a $\Delta G_{f}^{{}^{\circ}}$ of −58.9 kcal/mol, whereas the most stable Fe(III) complex with ASC and AMD, **{B5}** and **{B8}**, are 10–12 kcal/mol less stable). PM can chelate Fe(III), immobilizing it and preventing it from reacting with other molecules.

Concerning the AG complexes, we reported in our previous publication that the most stable complex of Fe(III) and AG displays a $\Delta G_{f}^{{}^{\circ}}$ value of −37.9 kcal/mol. This complex is much less stable than the most stable ASC complex, **{B8}**, with a $\Delta G_{f}^{{}^{\circ}}$ value of −46.9 kcal/mol. Our calculations show that AG is not capable of chelating Fe(III) ions as strongly as ASC does. Nonetheless, as we showed in the previously mentioned paper, this complex reduces the rate constant of the first step of the Haber-Weiss cycle by a factor of 10^5^, when the reductant is ASC^−^ (compared to the rate constant for the reduction of the Fe(III) hydrated ion). This would explain why AG can prevent the oxidation of ASC by this metal ion [23]. The calculated thermodynamic data for the most stable Fe(III) complexes with AG are displayed in Table 2.

As a last note, we would like to compare and contrast our results with the ones described in the 2012 paper by Ortega-Castro et al. [15]. This group calculated the $\Delta G_{f}^{{}^{\circ}}$ values of several complexes between Fe(III) and AG, ASC, AMD, PM, and LR-74 (an inhibitor of AGEs), and referenced it to the stability of a complex between iron and EDTA (ethylenediaminetetraacetic acid). The group used two different levels of theory to optimize these complexes: B3LYP(CPCM)/6-31+G(d) and M06(CPCM)/6-31+G(d,p). This group optimized four PM-containing complexes, three of which match our complexes **{C14}**, **{B10}**, and **{B12}**. In addition to these, Ortega-Castro et al. also calculated the low-spin analogue of complex **{B12}**. We did not optimize any low-spin complexes, as these were shown in our previous publication to invariably be more unstable than the high-spin ones [23]. For the B3LYP(CPCM)/6-31+G(d) calculations, Ortega-Castro et al. reported the following $\Delta G_{f}^{{}^{\circ}}$ values: −124.3, −120.8, and −154.5 kcal/mol (for our equivalent **{C14}**, **{B10}**, and **{B12}** complexes). On the other hand, their M06(CPCM)/6-31+G(d,p) results were: −121.2, −119.7, and −158.1 kcal/mol, respectively. In comparison, we obtained the following $\Delta G_{f}^{{}^{\circ}}$ values: −51.1, −42.1, and −58.9 kcal/mol, respectively. Despite the values being vastly different, the stability trend is the same: **{B12}** > **{C14}** > **{B10}**. Ortega-Castro et al. also optimized four AMD complexes. Three of these are equivalent to our **{C3}**, **{B4}**, and **{B5}** complexes. Furthermore, they optimized the low-spin analogue of **{B5}**, which we did not. The $\Delta G_{f}^{{}^{\circ}}$ values they obtained by means of the B3LYP(CPCM)/6-31+G(d) level of theory are: −61.2, −98.5, and −115.9 kcal/mol (for the complexes equivalent to our **{C3}**, **{B4}**, and **{B5}** complexes). Their M06(CPCM)/6-31+G(d,p) calculations yielded the following results: −49.1, −100.7, and −126.1 kcal/mol, respectively. The $\Delta G_{f}^{{}^{\circ}}$ values of our complexes are: −22.5, −42.8, and −48.9 kcal/mol, with respect to **{C3}**, **{B4}**, and **{B5}**. Once again, the values are different but the trends are maintained. The ASC complexes calculated by Ortega-Castro et al. cannot be compared to ours, for the ligand is bidentate in their work, whereas ours is solely monodentate. As previously stated, these discrepancies are explained by the different levels of theory used, the approach employed to calculate the value of $\Delta G_{f}^{{}^{\circ}}$, and other issues (stereochemistry of the ligands and pH considerations).

### 3.4. Kinetic Results of the Reduction of Cu(II) and Fe(III) when PM Acts as a Ligand

In our previous publications, we studied the secondary antioxidant activity of AG relative to both the reduction of Cu(II) and Fe(III) with the superoxide radical anion ($O_{2}^{•-}$) or ascorbate (ASC^−^) [22,23]. A compound is said to present secondary antioxidant activity if it is capable of chelating a metal ion (Cu(II) or Fe(III) in this case) and reducing the rate constant of the first step of the Haber-Weiss cycle, as shown in Equation (21) (note that the metal ion can either be Cu(II) or Fe(III)). If this first step is slowed (or even better, if it is inhibited), the second step is hindered as well, minimizing the formation of harmful ·OH radicals. The reductant (indicated by the pair Ox^−^/Ox) can either be $O_{2}^{•-}$ or ASC^−^. The calculated structures for the hydrated Cu(II) and Fe(III) and their reduction product, the hydrated Cu(I) and Fe(II) complexes, are shown in Figure S2.

(21) $$\begin{array}{c} {\left[Fe{\left(H_{2}O\right)}_{6}\right]}^{3+}+\;Ox^{-}\to {\left[Fe{\left(H_{2}O\right)}_{6}\right]}^{2+}+Ox\\ {\left[Fe{\left(H_{2}O\right)}_{6}\right]}^{2+}+\;H_{2}O_{2}\to {\left[Fe{\left(H_{2}O\right)}_{6}\right]}^{3+}+OH^{-}+OH^{.} \end{array}$$

So far, several experimental publications have consistently found that both AG and PM (among other molecules) can prevent the oxidation of ascorbic acid by Cu(II) via chelation, and it appears that the same is true for Fe(III) [6,28]. Moreover, other groups have investigated from a theoretical perspective the antioxidant activity of several organic molecules in the presence of cupric ions [44,49,50]. In addition to this, our group has recently published four papers in which the antioxidant activity of AG and dihydrolipoic acid is examined [22,23,29,44]. We found that these ligands can chelate both Cu(II) and Fe(III) and greatly reduce the rate constant of the first step of the Haber-Weiss cycle when the reductant is ascorbate, but not when it is $O_{2}^{•-}$. Leaving aside these studies, we have not been able to find any publications which analyze the redox chemistry of the Fe(III)/Fe(II) pair in the presence of AG or PM from a theoretical standpoint. Given the much greater thermodynamic stability of the Cu(II) and Fe(III) complexes with PM relative to those with AG, it is, thus, of interest to examine the secondary antioxidant activity of several PM complexes we have studied and put the results in perspective with respect to our previous findings.

**Figure 4:**
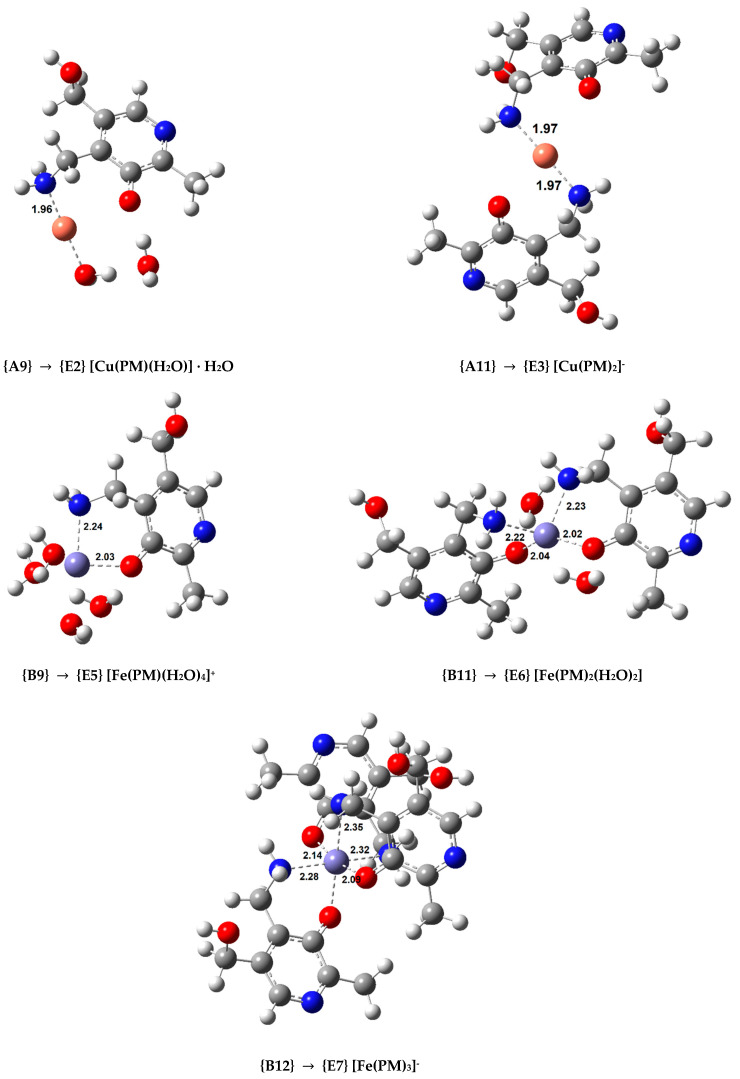
Optimized geometries of the Cu(I) and Fe(II) complexes with PM in aqueous solution (indicating the Cu(II) and Fe(III) complex used as a starting point in each case. Bond distances in Å).

In order to study the secondary antioxidant activity of PM, we selected the most stable complexes containing this ligand in different coordination patterns (complexes **{A9}**, **{A11}** with Cu(II), and complexes **{B9}**, **{B11}**, and **{B12}** with Fe(III)) and optimized the analogous Cu(I) and Fe(II) coordination compounds, whose structures are displayed in Figure 4. The Cu(I) complexes are linear while the Fe(II) complexes are octahedral. Afterward, the values of the rate constants (*k*) were calculated using both $O_{2}^{•-}$ or ASC^−^. The rate constants of the hydrated ion reduction were calculated as a reference. The resulting *k* values are shown in Table 4 in descending order, when compared to the reference reaction. Diffusion corrections were applied for the reduction reactions of the hydrated ions, and complexes **{A9}**, **{B9}**, and **{B11}** when the reducing agent is the superoxide anion, as the *k* values were larger than 10^8^. Additional kinetic and thermodynamic information on these reactions is displayed in Tables S4–S7.

The calculated rate constant for the Cu(II)/Cu(I) reduction with $O_{2}^{•-}$ for the reference reaction (7.71 × 10^9^ M^−1^ s^−1^) is in excellent agreement with the experimental value ((8.1 ± 0.5) × 10^9^ M^–1^ s^–1^) [51], which further validates our methodology. Two copper complexes were studied (**{A9}** and **{A11}**). Complex **{A9}** can reduce the rate constant of the reference reaction by a negligible amount when the reductant is $O_{2}^{•-}$, but by a factor of almost 10^6^ when ascorbate is being oxidized. On the other hand, complex **{A11}**, where Cu(II) is chelated by two bidentate PM ligands, is capable of significantly slowing down the reaction with both reducing agents. The rate constant is reduced by almost 10^4^ times when reacting with $O_{2}^{•-}$ (from 7.7 × 10^9^ to 8.0 × 10^5^ M^−1^ s^−1^) and by more than 10^13^ times when reacting with ASC^−^ (from 2.1 × 10^9^ to 5.1 × 10^−5^ M^−1^ s^−1^). PM, upon coordination with Cu(II), is able to inhibit ∙OH radical formation when reacting with ASC^−^, and can do the same when reacting with $O_{2}^{•-}$ if in a significant concentration to favour the formation of **{A11}**. In our previous studies, the highest reduction we have observed when $O_{2}^{•-}$ is the reductant was of 3.4 times with dihydrolipoic acid [29].

Three different Fe(III) complexes were considered for the kinetic calculations: **{B9}**, **{B11}**, and **{B12}**, each presenting an increasing number of bidentate PM ligands. **{B9}** is the only complex that actually speeds up the reaction by a small amount, which is a situation we observed with the AG complexes of iron and superoxide. This Fe(III) complex is also the one that slows the reaction with ASC^−^ the least, by a factor of 10^3^. Adding a second PM ligand in a *cis* fashion creates complex **{B11}**. This compound marginally reduces the rate constant of the reaction when superoxide is the reducing agent, but the rate constant reduction when the reactant is ASC^−^ is remarkable (going from 7.4 × 10^9^ to 3.9 × 10^2^ M^−1^ s^−1^, a *k* reduction that is greater than 10^7^ times). Chelating Fe(III) with a third bidentate ligand, as in complex **{B12}**, leads to a situation similar to that of the Cu(II) complex **{A11}**. **{B12}** is capable of significantly slowing down the reaction with both reducing agents, more than 10^3^ times when reacting with $O_{2}^{•-}$ (with a rate constant reduction from 7.3 × 10^9^ to 4.1 × 10^6^ M^−1^ s^−1^) and more than 10^15^ times when reacting with ASC^−^ (with a rate constant reduction from 7.4 × 10^9^ to 1.7 × 10^−6^ M^−1^ s^−1^). PM, upon coordinating with Fe(III), is able to inhibit ∙OH radical formation when reacting with ASC^−^, and can do the same when reacting with $O_{2}^{•-}$ if in a significant concentration to form a 1:3 complex.

These results contrast with our previous research with AG complexes. The most stable AG complex with Cu(II) reduced the rate constant relative to the reference reaction by a factor of 2.8 when the reactant is superoxide. On the other hand, the rate constant was reduced from 7.43 × 10^9^ to 60.9 M^−1^ s^−1^ in the presence of ASC^−^ by the same complex [22,23]. The complexes PM forms with Cu(II) are not only much more stable than with AG, but they can also reduce the rate constant of the first step of the Haber-Weiss cycle with both $O_{2}^{•-}$ and ASC^−^ to a greater extent. The same is true for Fe(III). First, none of the Fe(III) complexes with AG we studied reduced the rate constant of the reference reaction when the reducing agent was $O_{2}^{•-}$. The value of *k* was increased in all instances. Moreover, when the reactant was ASC^−^, the rate constant was reduced by a factor of 7.85 × 10^5^ by the most stable complex. The low-spin analogue of this coordination compound, much less stable, was capable of reducing *k* 1.66 × 10^9^ times. This complex, however, would be present in small amounts, given its low stability [23].

PM forms very stable complexes with both Fe(III) and Cu(II) and significantly slows down the rate constant of the first step of the Haber-Weiss cycle (with both the superoxide radical anion and ascorbate) exhibiting secondary antioxidant activity (alongside its already established primary antioxidant activity) [21]. Given that the PM complexes of Fe(III) are always more stable than the analogous ones of Cu(II) with the same number of ligands, PM will favour the chelation of Fe(III) over Cu(II). Moreover, the slowing down of the reaction with $O_{2}^{•-}$ will happen only when enough PM is present to form the much more stable 1:3 complexes.

## 4. Conclusions

Through this work, we have been able to show that ASC, AMD, and PM can form a wide array of stable complexes with both Cu(II) and Fe(III), the stability of which increases with the addition of ligands. When comparing these results with our previous calculations with AG complexes, we find that Cu(II) will form the most stable complexes with PM, which is followed by AMD. The AG complexes are of medium stability, and the least stable are the ASC ones. PM forms the most stable complexes with Fe(III) by a wide margin. AMD complexes follow, then ASC, and, finally, the AG coordination compounds (which we investigated in a previous publication). When comparing analogous complexes, the Fe(III) ones are more stable than the Cu(II) complexes (except for AG). Moreover, PM does possess significant secondary antioxidant activity. The most stable Cu(II) complex can reduce the rate constant of the reaction with superoxide 9.7 × 10^3^ times, and up to 4.1 × 10^13^ times when ascorbate is oxidized. When chelating Fe(III) and forming the most stable compound, the rate constant of the Fe(III) to Fe(II) reaction is reduced 1.8 × 10^3^ and 4.5 × 10^15^ times when the reductants are $O_{2}^{•-}$ and ascorbate, respectively. These results reveal that, at physiological conditions, PM will preferably form complexes with Fe(III), and, only at high concentrations of PM (when the 1:3 complex can be formed), the full potential of this ligand as a secondary antioxidant will be detected. However, the 1:3 complexes are significantly more stable than the 1:2 and 1:1 complexes.

With this research, our previous studies of AG as a glycation inhibitor are put in perspective with other inhibitors. As stated previously, a good glycation inhibitor should scavenge both carbonyl and radical species, and chelate metal ions. Our group has shown (following a consistent methodology) that AG is a mid-range scavenger of radical species. Moreover, it can form stable complexes with Cu(II) and Fe(III), but not as stable as those of a model Amadori compound. This indicates that AG cannot halt the oxidation of these compounds and the formation of AGEs. In addition, AG does have secondary antioxidant activity, but only when the reducing agent is ascorbate. On the other hand, PM has proved to be a much more potent secondary antioxidant, preventing the oxidation of the superoxide radical anion and of ascorbate. Furthermore, it coordinates with Cu(II) and Fe(III) forming complexes as stable (or even more) as those of AMD. All of this is in agreement with the experimental data. Both AG and PM can prevent the oxidation of ascorbate in the presence of Cu(II) and Fe(III), but only PM can hinder the formation of AGEs. The activity of AG as a carbonyl scavenger following the same level of theory employed in this study remains to be tested, as it has been theorized that this is the main mechanism of action of AG as a glycation inhibitor [20]. Finally, we would like to add that this collection of work on the primary and secondary antioxidant activities of AG and PM [20,21,22,23] sets the way for studies with potentially new drugs that now can be compared in both regards to these species.

## Figures and Tables

**Table 1:** Standard Gibbs free energy of formation ($\Delta G_{f}^{{}^{\circ}}$, in kcal/mol) and formation constant ($K_{f}$, $logK_{f}$) for the chelation of Cu(II) with AMD^−^, ASC^−^, and PM^−^ (as per Equations (6), (17), and (18), respectively) in aqueous solution at 298.15 K, taking into account the deprotonation energy for the AMD^−^ and PM-containing complexes. Similar values related to the most stable complexes with AG are included for comparison ^a^.

**COMPLEX** **${\left[Cu{\left(AMD\right)}_{x}{\left(H_{2}O\right)}_{n}\right]}^{\left(2-x\right)+}$**	$\Delta G_{f}^{{}^{\circ}}{}_{{}_{Cu^{2+}-AMD^{-}}}$	**$K_{f_{Cu^{2+}-AMD^{-}}}$**	**$logK_{f_{Cu^{2+}-AMD^{-}}}$**
**{A1}** [Cu(AMD)(H_2_O)_2_]^+^ (k, OH)	−1.8	20.2	1.31
**{A2}** [Cu(AMD)(H_2_O)_2_]^+^ (N, k)	−9.0	3.91 × 10^6^	6.59
**{A3}** [Cu(AMD)(H_2_O)_2_]^+^ (CO, N)	−19.7	2.54 × 10^14^	14.41
**{A4}** [Cu(AMD)(H_2_O)]^+^ (CO, N, k)	−20.7	1.48 × 10^15^	15.17
**{A5}** [Cu(AMD)_2_] (CO, N; mirror)	−33.6	3.93 × 10^24^	24.59
**{A6}** [Cu(AMD)_2_] (CO, N)	−35.3	7.28 × 10^25^	25.86
**COMPLEX** **${\left[Cu{\left(ASC\right)}_{x}{\left(H_{2}O\right)}_{n}\right]}^{\left(2-x\right)+}$**	**$\Delta G_{f}^{{}^{\circ}}{}_{{}_{Cu^{2+}-ASC^{-}}}$**	**$K_{f_{Cu^{2+}-ASC^{-}}}$**	**$logK_{f_{Cu^{2+}-ASC^{-}}}$**
**{A7}** [Cu(ASC)(H_2_O)_3_]^+^	−6.9	1.11 × 10^5^	5.05
**{A8}** [Cu(ASC)_2_(H_2_O)_2_]	−13.7	1.08 × 10^10^	10.03
**COMPLEX** **${\left[Cu{\left(PM\right)}_{x}{\left(H_{2}O\right)}_{n}\right]}^{\left(2-x\right)+}$**	**$\Delta G_{f}^{{}^{\circ}}{}_{{}_{Cu^{2+}-PM^{-}}}$**	**$K_{f_{Cu^{2+}-PM^{-}}}$**	**$logK_{f_{Cu^{2+}-PM^{-}}}$**
**{A9}** [Cu(PM)(H_2_O)_2_]^+^	−19.4	1.67 × 10^14^	14.22
**{A10}** [Cu(PM)_2_] (mirror image)	−33.8	5.49 × 10^24^	24.74
**{A11}** [Cu(PM)_2_]	−35.8	1.69 × 10^26^	26.23
**COMPLEX** ^b^ **${\left[Cu{\left(AG\right)}_{x}{\left(H_{2}O\right)}_{n}\right]}^{2+}$**	**$\Delta G_{f}^{{}^{\circ}}{}_{{}_{Cu^{2+}-AG^{}}}$**	**$K_{f_{Cu^{2+}-AG^{}}}$**	**$logK_{f_{Cu^{2+}-AG^{}}}$**
**{G1}** [Cu(AG)(H_2_O)_2_]^2+^	−16.3	8.75 × 10^11^	11.94
**{G2}** [Cu(AG)_2_]^2+^ (mirror image)	−29.7	6.25 × 10^21^	21.80

^a^ Coordinating atoms in the Amadori model compound are shown in parentheses for each complex: k for the ketone, OH for the alcohol group, N for the amine group, and CO for the carboxylate group. All the complexes containing ascorbate ligands coordinate through the deprotonated hydroxyl group at position 4. Similarly, the pyridoxamine ligand only bonds through the phenolate and the amine group in the methylamine chain. ^b^ Results taken from Reference [22].

**Table 2:** Standard Gibbs free energy of formation ($\Delta G_{f}^{{}^{\circ}}$, in kcal/mol) and formation constant ($K_{f}$, $logK_{f}$) for the octahedral complexes of Fe(III) with AMD^−^, ASC^−^, and PM^−^ (as per Equations (9), (19), and (20), respectively) in aqueous solution at 298.15 K, taking into account the deprotona-tion energy for the AMD^−^ and PM-containing complexes. Similar values related to the most stable complexes with AG are included for compar-ison ^a^.

**COMPLEX** **${\left[Fe{\left(AMD\right)}_{x}{\left(H_{2}O\right)}_{n}\right]}^{\left(3-x\right)+}$**	**$\Delta G_{f}^{{}^{\circ}}{}_{{}_{Fe^{3+}-AMD^{-}}}$**	**$K_{f_{Fe^{3+}-AMD^{-}}}$**	**$logK_{f_{Fe^{3+}-AMD^{-}}}$**
**{B1}** [Fe(AMD)(H_2_O)_4_]^2+^ (CO, N)	−20.1	5.07 × 10^14^	14.71
**{B2}** [Fe(AMD)(H_2_O)_3_]^2+^ (CO, N, k)	−23.4	1.39 × 10^17^	17.14
**{B3}** [Fe(AMD)_2_(H_2_O)_2_]^+^ (CO, N)	−37.4	2.60 × 10^27^	27.41
**{B4}** [Fe(AMD)_2_]^+^ (CO, N, k)	−42.8	2.24 × 10^31^	31.35
**{B5}** [Fe(AMD)_3_] (CO, N)	−48.9	6.72 × 10^35^	35.83
**COMPLEX** **${\left[Fe{\left(ASC\right)}_{x}{\left(H_{2}O\right)}_{n}\right]}^{\left(3-x\right)+}$**	**$\Delta G_{f}^{{}^{\circ}}{}_{{}_{Fe^{3+}-ASC^{-}}}$**	**$K_{f_{Fe^{3+}-ASC^{-}}}$**	**$logK_{f_{Fe^{3+}-ASC^{-}}}$**
**{B6}** [Fe(ASC)(H_2_O)_5_]^2+^	−19.3	1.30 × 10^14^	14.11
**{B7}** [Fe(ASC)_2_(H_2_O)_4_]^+^	−28.3	5.83 × 10^20^	20.77
**{B8}** [Fe(ASC)_3_(H_2_O)_3_]	−46.9	2.20 × 10^34^	34.34
**COMPLEX** **${\left[Fe{\left(PM\right)}_{x}{\left(H_{2}O\right)}_{n}\right]}^{\left(3-x\right)+}$**	$\Delta G_{f}^{{}^{\circ}}{}_{{}_{Fe^{3+}-PM^{-}}}$	**$K_{f_{Fe^{3+}-PM^{-}}}$**	**$logK_{f_{Fe^{3+}-PM^{-}}}$**
**{B9}** [Fe(PM)(H_2_O)_4_]^2+^	−28.4	6.90 × 10^20^	20.84
**{B10}** [Fe(PM)_2_(H_2_O)_2_]^+^ *trans*	−42.1	7.83 × 10^30^	30.89
**{B11}** [Fe(PM)_2_(H_2_O)_2_]^+^ *cis*	−45.8	3.48 × 10^33^	33.54
**{B12}** [Fe(PM)_3_]	−58.9	1.56 × 10^43^	43.19
**COMPLEX** ^b^ **${\left[Fe\left(AG\right)x{\left(H_{2}O\right)}_{n}\right]}^{3+}$**	**$\Delta G_{f}^{{}^{\circ}}{}_{{}_{Fe^{3+}-AG^{}}}$**	**$K_{f_{Fe^{3+}-AG^{}}}$**	**$logK_{f_{Fe^{3+}-AG^{}}}$**
**{G3}** [Fe(AG)(H_2_O)_4_]^3+^	−10.3	3.48 × 10^7^	7.54
**{G4}** [Fe(AG)_2_(H_2_O)]^3+^ (5-coord.)	−23.5	1.78 × 10^17^	17.25
**{G5}** [Fe(AG)_3_]^3+^ (same orient.)	−37.9	5.65 × 10^27^	27.75

^a^ Coordinating atoms in the Amadori model compound are shown in parentheses for each complex: k for the ketone, OH for the alcohol group, N for the amine group, and CO for the carboxylate group. All the complexes containing ascorbate ligands coordinate through the deprotonated hydroxyl group at position 4. Similarly, the pyridoxamine ligand only bonds through the phenolate and the amine group in the methylamine chain. ^b^ Results taken from Reference [23].

**Table 3:** Standard Gibbs free energy of formation ($\Delta G_{f}^{{}^{\circ}}$, in kcal/mol) and formation constant ($K_{f}$, $logK_{f}$) for the non-octahedral complexes of Fe(III) with AMD^−^, ASC^−^, and PM^−^ (as per Equations (9), (19), and (20), respectively) in aqueous solution at 298.15 K, taking into account the deprotonation energy for the AMD^−^ and PM-containing complexes ^a^.

**COMPLEX** **${\left[Fe{\left(AMD\right)}_{x}{\left(H_{2}O\right)}_{n}\right]}^{\left(3-x\right)+}$**	$\Delta G_{f}^{{}^{\circ}}{}_{{}_{Fe^{3+}-AMD^{-}}}$	**$K_{f_{Fe^{3+}-AMD^{-}}}$**	**$logK_{f_{Fe^{3+}-AMD^{-}}}$**
**{C1}** [Fe(AMD)(H_2_O)_2_]^2+^ (4-coord.) (CO, N)	−18.4	3.34 × 10^13^	13.52
**{C2}** [Fe(AMD)(H_2_O)_3_]^2+^ (5-coord.) (CO, N)	−22.1	1.59 × 10^16^	16.20
**{C3}** [Fe(AMD)(H_2_O)]^2+^ (4-coord.) (CO, N, k)	−22.5	3.17 × 10^16^	16.50
**{C4}** [Fe(AMD)(H_2_O)_2_]^2+^ (5-coord.) (CO, N, k)	−27.3	9.55 × 10^19^	19.98
**{C5}** [Fe(AMD)_2_]^+^ (4-coord.) (CO, N)	−41.7	3.40 × 10^30^	30.53
**{C6}** [Fe(AMD)_2_(H_2_O)]^+^ (5-coord.) (CO, N)	−41.7	3.77 × 10^30^	30.58
**COMPLEX** **${\left[Fe{\left(ASC\right)}_{x}{\left(H_{2}O\right)}_{n}\right]}^{\left(3-x\right)+}$**	$\Delta G_{f}^{{}^{\circ}}{}_{{}_{Fe^{3+}-ASC^{-}}}$	**$K_{f_{Fe^{3+}-ASC^{-}}}$**	**$logK_{f_{Fe^{3+}-ASC^{-}}}$**
**{C7}** [Fe(ASC)(H_2_O)_4_]^2+^ (5-coord.)	−22.6	3.78 × 10^16^	16.58
**{C8}** [Fe(ASC)(H_2_O)_3_]^2+^ (4-coord.)	−25.7	6.55 × 10^18^	18.82
**{C9}** [Fe(ASC)_2_(H_2_O)_2_]^+^ (4-coord.)	−28.3	5.15 × 10^20^	20.71
**COMPLEX** **${\left[Fe{\left(PM\right)}_{x}{\left(H_{2}O\right)}_{n}\right]}^{\left(3-x\right)+}$**	$\Delta G_{f}^{{}^{\circ}}{}_{{}_{Fe^{3+}-PM^{-}}}$	**$K_{f_{Fe^{3+}-PM^{-}}}$**	**$logK_{f_{Fe^{3+}-PM^{-}}}$**
**{C10}** [Fe(PM)(H_2_O)_3_]^2+^ (5-coord.)	−24.9	1.88 × 10^18^	18.27
**{C11}** [Fe(PM)(H_2_O)_2_]^2+^ (4-coord.)	−26.5	2.80 × 10^19^	19.45
**{C12}** [Fe(PM)_2_(H_2_O)]^+^ (5-coord.) non-planar	−46.3	8.29 × 10^33^	33.92
**{C13}** [Fe(PM)_2_(H_2_O)]^+^ (5-coord.) planar	−48.0	1.46 × 10^35^	35.16
**{C14}** [Fe(PM)_2_]^+^ tetrahedral (4-coord.)	−51.1	2.84 × 10^37^	37.45

^a^ Coordinating atoms in the Amadori model compound are shown in parentheses for each complex: k for the ketone, OH for the alcohol group, N for the amine group, and CO for the carboxylate group. All the complexes containing ascorbate ligands coordinate through the deprotonated hydroxyl group at position 4. Similarly, the pyridoxamine ligand only bonds through the phenolate and the amine group in the methylamine chain.

**Table 4:** Rate constants (in M^−1^ s^−1^) for the reduction of Fe(III) and Cu(II) complexes (with and without complexation with PM^−^) with $O_{2}^{•-}$ and ascorbate ($ASC^{-}$) in aqueous solution at 298.15 K and the rate constant ratios (using the reduction of ${\left[Fe{\left(H_{2}O\right)}_{6}\right]}^{3+}$ or ${\left[Cu{\left(H_{2}O\right)}_{4}\right]}^{2+}$ as a reference) ^a^.

	$Ox^{-}=O_{2}^{•-}$		$Ox^{-}$ $=ASC^{-}$	
Reaction	*k_app_*	Ratio	*k_app_*	Ratio
${\left[Cu{\left(H_{2}O\right)}_{4}\right]}^{2+}+Ox^{-}\to {\left[Cu{\left(H_{2}O\right)}_{2}\right]}^{+}\cdot \;2H_{2}O+Ox$	7.71 × 10^9 b^		2.10 × 10^9^	
**{A9}**$+{\;\mathrm{Ox}}^{-}$→**{E2}** + Ox	5.53 × 10^9^	1.39	2.47 × 10^3^	8.50 × 10^5^
**{A11}**$+{\;\mathrm{Ox}}^{-}$→**{E3}** + Ox	7.95 × 10^5^	9.70 × 10^3^	5.13 × 10^−5^	4.09 × 10^13^
${\left[Fe{\left(H_{2}O\right)}_{6}\right]}^{3+}+Ox^{-}\to {\left[Fe{\left(H_{2}O\right)}_{6}\right]}^{2+}+Ox$	7.28 × 10^9^		7.43 × 10^9^	
$\left\{B9\right\}+{\;\mathrm{Ox}}^{-}\to \left\{E5\right\}+\mathrm{Ox}$	8.21 × 10^9^	0.89	3.99 × 10^6^	1.86 × 10^3^
$\left\{B11\right\}+{\;\mathrm{Ox}}^{-}\to \left\{E6\right\}+\mathrm{Ox}$	7.10 × 10^9^	1.02	3.91 × 10^2^	1.90 × 10^7^
$\left\{B12\right\}+{\;\mathrm{Ox}}^{-}\to \left\{E7\right\}+\mathrm{Ox}$	4.08 × 10^6^	1.78 × 10^3^	1.67 × 10^−6^	4.45 × 10^15^

^a^ For additional kinetic and thermodynamic information on these reactions, refer to Tables S4–S7. *k* is reported instead of *k_app_* when diffusion corrections were not necessary. ^b^ k_exp_ = 8.1 ± 0.5 × 10^9^ M^−1^ s^−1^ [51].

## Data Availability

Not applicable.

## Supplementary Materials

The following are available online at https://www.mdpi.com/2076-3921/10/2/208/s1. Table S1: Absolute enthalpies and Gibbs free energies of the different species considered in this study at the M05(SMD)/6-311+G(d,p) level of theory in water at 298.15 K. Table S2: $\langle {\hat{S}}^{2}\rangle$ values for the calculated open-shell copper and iron complexes before and after annihilation of the first spin contaminant. Table S3: Standard formation Gibbs free energy change ($\Delta G_{f}^{{}^{\circ}}$) and formation constant ($K_{f}$, $logK_{f}$) for the calculated complexes of Cu(II) and Fe(III) with ASC^−^ with unusually low coordination numbers in aqueous solution at 298.15 K. Table S4: Standard Gibbs free energy of reaction (ΔG°) and activation (ΔG^≠^), various rate constants, and the rate constant ratio (using k_app_ for the reduction of ${\left[Cu{\left(H_{2}O\right)}_{4}\right]}^{2+}$ as a reference) for the initial reaction of the Haber-Weiss cycle (with and without iron complexation with PM) with $O_{2}^{•-}$ in aqueous solution at 298.15 K. Table S5: Standard Gibbs free energy of the reaction (ΔG°) and activation (ΔG^≠^), various rate constants, and the rate constant ratio (using k_app_ for the reduction of ${\left[Cu{\left(H_{2}O\right)}_{4}\right]}^{2+}$ as a reference) for the initial reaction of the Haber-Weiss cycle (with and without iron complexation with PM) with ascorbate ($ASC^{-}$) in aqueous solution at 298.15 K. Table S6: Standard Gibbs free energy of reaction (ΔG°) and activation (ΔG^≠^), various rate constants, and the rate constant ratio (using k_app_ for the reduction of ${\left[Fe{\left(H_{2}O\right)}_{6}\right]}^{3+}$ as a reference) for the initial reaction of the Haber-Weiss cycle (with and without iron complexation with PM) with $O_{2}^{•-}$ in aqueous solution at 298.15 K. Table S7: Standard Gibbs free energy of reaction (ΔG°) and activation (ΔG^≠^), various rate constants, and the rate constant ratio (using k_app_ for the reduction of ${\left[Fe{\left(H_{2}O\right)}_{6}\right]}^{3+}$ as a reference) for the initial reaction of the Haber-Weiss cycle (with and without iron complexation with PM) with ascorbate ($ASC^{-}$) in aqueous solution at 298.15 K. Figure S1: Optimized geometries of the calculated complexes of Cu(II) and Fe(III) with ASC^−^ with unusually low coordination numbers in aqueous solution (bond distances in Å). Figure S2: Optimized geometries of the most stable hydrated Cu(II), Cu(I), Fe(III), and Fe(II) complexes in aqueous solution (bond distances in Å). Appendix 1: Additional details regarding the calculation of rate constants. Appendix 2: Additional explanation on the pK_a_ calculation for the neutral (zwitterion) model Amadori compound. Appendix 3: Additional explanation on the pK calculation for the equilibrium between protonated pyridoxamine H_2_PM^+^ and the anionic form PM^−^. M05(SMD)/6-311+G(d,p) Cartesian coordinates of the optimized geometries in water of the species calculated in this study.
