# Supplementary material for: Complexes of Copper and Iron with Pyridoxamine, Ascorbic Acid, and a Model Amadori Compound: Exploring Pyridoxamine’s Secondary Antioxidant Activity
Source: Antioxidants (Basel). 2021 Feb 1;10(2):208. doi: 10.3390/antiox10020208 (PMC7912584; doi:10.3390/antiox10020208)

**Complexes of copper and iron with pyridoxamine, ascorbate and a model Amadori compound: Exploring pyridoxamine's secondary antioxidant activity**

Guillermo García-Díez, Roger Monreal-Corona, Nelaine Mora-Diez*

Thompson Rivers University, Department of Chemistry, Kamloops, B.C., V2C 0C8 Canada

# Electronic Supplementary Information

(58 pages)

## Contents:

**Table S1.** Absolute enthalpies and Gibbs free energies of the different species considered in this study at the M05(SMD)/6-311+G(d,p) level of theory in water at 298.15 K.

**Table S2.** $\langle \hat{S}^2 \rangle$ values for the calculated open-shell copper and iron complexes before and after annihilation of the first spin contaminant.

**Table S3.** Standard formation Gibbs free energy change ($\Delta G_f^\circ$, kcal/mol) and formation constant ($K_f$, $logK_f$) for the calculated complexes of Cu(II) and Fe(III) with $ASC^-$ with unusually low coordination numbers in aqueous solution at 298.15 K.

**Table S4.** Standard Gibbs free energy of reaction (ΔG°, kcal/mol) and activation (ΔG≠, kcal/mol), various rate constants (k, $k_D$ and $k_{app}$, $M^{-1}$ $s^{-1}$) and the rate constant ratio (using $k_{app}$ for the reduction of $[Cu(H_2O)_4]^{2+}$ as reference) for the initial reaction of the Haber-Weiss cycle (with and without iron complexation with PM) with $O_2^{\bullet-}$ in aqueous solution at 298.15 K.

**Table S5.** Standard Gibbs free energy of reaction (ΔG°, kcal/mol) and activation (ΔG≠, kcal/mol), various rate constants (k, $k_D$ and $k_{app}$, $M^{-1}$ $s^{-1}$) and the rate constant ratio (using $k_{app}$ for the reduction of $[Cu(H_2O)_4]^{2+}$ as reference) for the initial reaction of the Haber-Weiss cycle (with and without iron complexation with PM) with ascorbate ($ASC^-$) in aqueous solution at 298.15 K.

**Table S6.** Standard Gibbs free energy of reaction (ΔG°, kcal/mol) and activation (ΔG≠, kcal/mol), various rate constants (k, $k_D$ and $k_{app}$, $M^{-1}$ $s^{-1}$) and the rate constant ratio (using $k_{app}$ for the reduction of $[Fe(H_2O)_6]^{3+}$ as reference) for the initial reaction of the Haber-Weiss cycle (with and without iron complexation with PM) with $O_2^{\bullet-}$ in aqueous solution at 298.15 K.

**Table S7.** Standard Gibbs free energy of reaction (ΔG°, kcal/mol) and activation (ΔG≠, kcal/mol), various rate constants (k, $k_D$ and $k_{app}$, $M^{-1}$ $s^{-1}$) and the rate constant ratio (using $k_{app}$ for the reduction of $[Fe(H_2O)_6]^{3+}$ as reference) for the initial reaction of the Haber-Weiss cycle (with and without iron complexation with PM) with ascorbate ($ASC^-$) in aqueous solution at 298.15 K.

* Corresponding author e-mail: nmora@tru.ca

**Figure S1.** Optimized geometries of the calculated complexes of Cu(II) and Fe(III) with $ASC^-$ with unusually low coordination numbers in aqueous solution (bond distances in Å).

**Figure S2.** Optimized geometries of the most stable hydrated Cu(II), Cu(I), Fe(III) and Fe(II) complexes in aqueous solution (bond distances in Å); both iron complexes are high spin.

**Appendix 1.** Additional details regarding the calculation of the rate constants.

**Appendix 2.** Additional explanation on the $pK_a$ calculation for the neutral (zwitterion) model Amadori compound.

**Appendix 3.** Additional explanation on the pK calculation for the equilibrium between protonated pyridoxamine $H_2PM^+$ and the anionic form $PM^-$.

M05(SMD)/6-311+G(d,p) Cartesian coordinates of the optimized geometries in water of the species calculated in this study.

**Table S1.** Absolute enthalpies and Gibbs free energies of the different species considered in this study at the M05(SMD)/6-311+G(d,p) level of theory in water at 298.15 K.

| | Species | H° (au) | G° (au) |
|---|---|---|---|
| {A1} | $[Cu(AMD)(H_2O)_2]^+$ (k, OH) | -2383.157242 | -2383.223632 |
| {A2} | $[Cu(AMD)(H_2O)_2]^+$ (N, k) | -2383.168403 | -2383.235125 |
| {A3} | $[Cu(AMD)(H_2O)_2]^+$ (CO, N) | -2383.185705 | -2383.252111 |
| {A4} | $[Cu(AMD)(H_2O)]^+$ (CO, N, k) | -2306.775679 | -2306.835380 |
| {A5} | $[Cu(AMD)_2]$ (CO, N; mirror image) | -2820.520524 | -2820.604359 |
| {A6} | $[Cu(AMD)_2]$ (CO, N) | -2820.520607 | -2820.607115 |
| {A7} | $[Cu(ASC)(H_2O)_3]^+$ | -2553.498486 | -2553.572102 |
| {A8} | $[Cu(ASC)_2(H_2O)_2]$ | -3161.155384 | -3161.254985 |
| {A9} | $[Cu(PM)(H_2O)_2]^+$ | -2364.205990 | -2364.271336 |
| {A10} | $[Cu(PM)_2]$ (mirror image) | -2782.554828 | -2782.638992 |
| {A11} | $[Cu(PM)_2]$ | -2782.556474 | -2782.642229 |
| | | | |
| {B1} | $[Fe(AMD)(H_2O)_4]^{2+}$ | -2159.111785 | -2159.187045 |
| {B2} | $[Fe(AMD)(H_2O)_3]^{2+}$ | -2082.704888 | -2082.773955 |
| {B3} | $[Fe(AMD)_2(H_2O)_2]^+$ | -2596.449124 | -2596.544772 |
| {B4} | $[Fe(AMD)_2]^+$ | -2443.634657 | -2443.717116 |
| {B5} | $[Fe(AMD)_3]$ | -3033.783198 | -3033.892945 |
| {B6} | $[Fe(ASC)(H_2O)_5]^{2+}$ | -2329.442475 | -2329.526095 |
| {B7} | $[Fe(ASC)_2(H_2O)_4]^+$ | -2937.106467 | -2937.212603 |
| {B8} | $[Fe(ASC)_3(H_2O)_3]$ | -3544.769220 | -3544.897186 |
| {B9} | $[Fe(PM)(H_2O)_4]^{2+}$ | -2140.140570 | -2140.220000 |
| {B10} | $[Fe(PM)_2(H_2O)_2]^+$ *trans* | -2558.493091 | -2558.586654 |
| {B11} | $[Fe(PM)_2(H_2O)_2]^+$ *cis* | -2558.496888 | -2558.592411 |
| {B12} | $[Fe(PM)_3]$ | -2976.844512 | -2976.954114 |
| | | | |
| {C1} | $[Fe(AMD)(H_2O)_2]^{2+}$ (4-coord.) | -2006.281349 | -2006.347340 |
| {C2} | $[Fe(AMD)(H_2O)_3]^{2+}$ (5-coord.) | -2082.700877 | -2082.771906 |
| {C3} | $[Fe(AMD)(H_2O)]^{2+}$ (4-coord.) | -1929.876757 | -1929.936790 |
| {C4} | $[Fe(AMD)(H_2O)_2]^{2+}$ (5-coord.) | -2006.296791 | -2006.361377 |
| {C5} | $[Fe(AMD)_2]^+$ (4-coord.) | -2443.630456 | -2443.715336 |
| {C6} | $[Fe(AMD)_2(H_2O)]^+$ (5-coord.) | -2520.044562 | -2520.134623 |
| {C7} | $[Fe(ASC)(H_2O)_4]^{2+}$ (5-coord.) | -2253.033728 | -2253.111602 |
| {C8} | $[Fe(ASC)(H_2O)_3]^{2+}$ (4-coord.) | -2176.629503 | -2176.698078 |
| {C9} | $[Fe(ASC)_2(H_2O)_2]^+$ (4-coord.) | -2784.278468 | -2784.375350 |
| {C10} | $[Fe(PM)(H_2O)_3]^{2+}$ (5-coord.) | -2063.724489 | -2063.796034 |
| {C11} | $[Fe(PM)(H_2O)_2]^{2+}$ (4-coord.) | -1987.314979 | -1987.379840 |
| {C12} | $[Fe(PM)_2(H_2O)]^+$ (5-coord.) non-planar | -2482.087165 | -2482.176206 |

| | | | |
|---|---|---|---|
| {C13} | $[Fe(PM)_2(H_2O)]^+$ (5-coord.) planar | -2482.088724 | -2482.178914 |
| {C14} | $[Fe(PM)_2]^+$ tetrahedral (4-coord.) | -2405.681532 | -2405.764702 |
| | | | |
| {D1} | $[Cu(ASC)(H_2O)_2]^+$ (3-coord.) | -2477.089038 | -2477.156659 |
| {D2} | $[Cu(ASC)_2]$ (2-coord.) | -3008.333883 | -3008.424369 |
| {D3} | $[Fe(ASC)(H_2O)_2]^{2+}$ (3-coord.) | -2100.200962 | -2100.269594 |
| {D4} | $[Fe(ASC)_2]^+$ (2-coord.) | -2631.447417 | -2631.538605 |
| {D5} | $[Fe(ASC)_2(H_2O)]^+$ (3-coord.) | -2707.865995 | -2707.960754 |
| {D6} | $[Fe(ASC)_3]$ (3-coord.) | -3315.538126 | -3315.653006 |
| | | | |
| {E1} | $[Cu(H_2O)_2]^+ \cdot 2H_2O$ | -1946.026769 | -1946.081005 |
| {E2} | $[Cu(PM)(H_2O)] \cdot H_2O$ | -2364.368739 | -2364.434269 |
| {E3} | $[Cu(PM)_2]^-$ | -2782.694000 | -2782.781644 |
| {E4} | $[Fe(H_2O)_6]^{2+}$ | -1721.959274 | -1722.023834 |
| {E5} | $[Fe(PM)(H_2O)_4]^+$ | -2140.304596 | -2140.384189 |
| {E6} | $[Fe(PM)_2(H_2O)_2]$ | -2558.649481 | -2558.747734 |
| {E7} | $[Fe(PM)_3]^-$ | -2976.971137 | -2977.088005 |
| | | | |
| {F1} | $[Cu(H_2O)_4]^{2+}$ | -1945.844939 | -1945.891638 |
| {F2} | $[Fe(H_2O)_6]^{3+}$ | -1721.770188 | -1721.825920 |
| {F3} | $AMD^-$ | -590.120229 | -590.172764 |
| {F4} | $[(AMD)_2]^{2-}$ | -1180.253361 | -1180.336430 |
| {F5} | $[(AMD)_3]^{3-}$ | -1770.385423 | -1770.498959 |
| {F6} | $ASC^-$ | -684.045489 | -684.096816 |
| {F7} | $[(ASC)_2]^{2-}$ | -1368.105837 | -1368.188712 |
| {F8} | $[(ASC)_3]^{3-}$ | -2052.149882 | -2052.262171 |
| {F9} | $PM^-$ | -571.133806 | -571.184492 |
| {F10} | $[(PM)_2]^{2-}$ | -1142.269060 | -1142.354964 |
| {F11} | $[(PM)_3]^{3-}$ | -1713.403739 | -1713.520440 |
| {F12} | $H_2O$ | -76.405902 | -76.427325 |
| {F13} | $(H_2O)_2$ | -152.814809 | -152.847175 |
| {F14} | $(H_2O)_3$ | -229.224017 | -229.265566 |
| {F15} | $(H_2O)_4$ | -305.636444 | -305.684310 |
| {F16} | $(H_2O)_5$ | -382.046592 | -382.101334 |
| {F17} | $(H_2O)_6$ | -458.460845 | -458.520521 |
| | | | |
| {G1} | $[Cu(AG_D)(H_2O)_2]^{2+}$ | -2053.584492 | -2053.636206 |
| {G2} | $[Cu(AG_D)_2]^{2+}$ (mirror image) | -2161.321112 | -2161.375573 |
| {G3} | $[Fe(AG)(H_2O)_4]^{3+}$ | -1829.500673 | -1829.560921 |
| {G4} | $[Fe(AG)_2(H_2O)]^{3+}$ (5-coord.) | -1860.822714 | -1860.882948 |
| {G5} | $[Fe(AG_D)_3]^{3+}$ (same orientation) | -2044.965806 | -2045.037927 |

**Table S2.** $\langle \hat{S}^2 \rangle$ values for the calculated open-shell copper and iron complexes before and after annihilation of the first spin contaminant.

| | Species | $\langle \hat{S}^2 \rangle$ before annihilation | $\langle \hat{S}^2 \rangle$ after annihilation |
|---|---|---|---|
| {A1} | $[Cu(AMD)(H_2O)_2]^+$ (k, OH) | 0.7520 | 0.7500 |
| {A2} | $[Cu(AMD)(H_2O)_2]^+$ (N, k) | 0.7524 | 0.7500 |
| {A3} | $[Cu(AMD)(H_2O)_2]^+$ (CO, N) | 0.7527 | 0.7500 |
| {A4} | $[Cu(AMD)(H_2O)]^+$ (CO, N, k) | 0.7526 | 0.7500 |
| {A5} | $[Cu(AMD)_2]$ (CO, N; mirror image) | 0.7530 | 0.7500 |
| {A6} | $[Cu(AMD)_2]$ (CO, N) | 0.7523 | 0.7500 |
| {A7} | $[Cu(ASC)(H_2O)_3]^+$ | 0.7528 | 0.7500 |
| {A8} | $[Cu(ASC)_2(H_2O)_2]$ | 0.7522 | 0.7500 |
| {A9} | $[Cu(PM)(H_2O)_2]^+$ | 0.7528 | 0.7500 |
| {A10} | $[Cu(PM)_2]$ (mirror image) | 0.7530 | 0.7500 |
| {A11} | $[Cu(PM)_2]$ | 0.7531 | 0.7500 |
| | | | |
| {B1} | $[Fe(AMD)(H_2O)_4]^{2+}$ | 8.7560 | 8.7500 |
| {B2} | $[Fe(AMD)(H_2O)_3]^{2+}$ | 8.7570 | 8.7500 |
| {B3} | $[Fe(AMD)_2(H_2O)_2]^+$ | 8.7571 | 8.7500 |
| {B4} | $[Fe(AMD)_2]^+$ | 8.7577 | 8.7500 |
| {B5} | $[Fe(AMD)_3]$ | 8.7578 | 8.7500 |
| {B6} | $[Fe(ASC)(H_2O)_5]^{2+}$ | 8.7650 | 8.7500 |
| {B7} | $[Fe(ASC)_2(H_2O)_4]^+$ | 8.7607 | 8.7500 |
| {B8} | $[Fe(ASC)_3(H_2O)_3]$ | 8.7621 | 8.7500 |
| {B9} | $[Fe(PM)(H_2O)_4]^{2+}$ | 8.7648 | 8.7501 |
| {B10} | $[Fe(PM)_2(H_2O)_2]^+$ *trans* | 8.7612 | 8.7500 |
| {B11} | $[Fe(PM)_2(H_2O)_2]^+$ *cis* | 8.7672 | 8.7500 |
| {B12} | $[Fe(PM)_3]$ | 8.7618 | 8.7500 |
| | | | |
| {C1} | $[Fe(AMD)(H_2O)_2]^{2+}$ (4-coord.) | 8.7566 | 8.7500 |
| {C2} | $[Fe(AMD)(H_2O)_3]^{2+}$ (5-coord.) | 8.7568 | 8.7500 |
| {C3} | $[Fe(AMD)(H_2O)]^{2+}$ (4-coord.) | 8.7573 | 8.7500 |
| {C4} | $[Fe(AMD)(H_2O)_2]^{2+}$ (5-coord.) | 8.7575 | 8.7500 |
| {C5} | $[Fe(AMD)_2]^+$ (4-coord.) | 8.7585 | 8.7500 |
| {C6} | $[Fe(AMD)_2(H_2O)]^+$ (5-coord.) | 8.7579 | 8.7500 |
| {C7} | $[Fe(ASC)(H_2O)_4]^{2+}$ (5-coord.) | 8.7662 | 8.7500 |
| {C8} | $[Fe(ASC)(H_2O)_3]^{2+}$ (4-coord.) | 8.7643 | 8.7500 |
| {C9} | $[Fe(ASC)_2(H_2O)_2]^+$ (4-coord.) | 8.7646 | 8.7501 |
| {C10} | $[Fe(PM)(H_2O)_3]^{2+}$ (5-coord.) | 8.7663 | 8.7501 |
| {C11} | $[Fe(PM)(H_2O)_2]^{2+}$ (4-coord.) | 8.7669 | 8.7501 |
| {C12} | $[Fe(PM)_2(H_2O)]^+$ (5-coord.) non-planar | 8.7630 | 8.7500 |

| | | | |
|---|---|---|---|
| {C13} | $[Fe(PM)_2(H_2O)]^+$ (5-coord.) planar | 8.7604 | 8.7500 |
| {C14} | $[Fe(PM)_2]^+$ tetrahedral (4-coord.) | 8.7640 | 8.7501 |
| | | | |
| {D1} | $[Cu(ASC)(H_2O)_2]^+$ (3-coord.) | 0.7521 | 0.7500 |
| {D2} | $[Cu(ASC)_2]$ (2-coord.) | 0.7524 | 0.7500 |
| {D3} | $[Fe(ASC)(H_2O)_2]^{2+}$ (3-coord.) | 8.7730 | 8.7501 |
| {D4} | $[Fe(ASC)_2]^+$ (2-coord.) | 8.7687 | 8.7501 |
| {D5} | $[Fe(ASC)_2(H_2O)]^+$ (3-coord.) | 8.7663 | 8.7501 |
| {D6} | $[Fe(ASC)_3]$ (3-coord.) | 8.7628 | 8.7500 |
| | | | |
| {E4} | $[Fe(H_2O)_6]^{2+}$ | 6.0104 | 6.0000 |
| {E5} | $[Fe(PM)(H_2O)_4]^+$ | 6.0122 | 6.0000 |
| {E6} | $[Fe(PM)_2(H_2O)_2]$ | 6.0143 | 6.0000 |
| {E7} | $[Fe(PM)_3]^-$ | 6.0138 | 6.0000 |
| | | | |
| {F1} | $[Cu(H_2O)_4]^{2+}$ | 0.7519 | 0.7500 |
| {F2} | $[Fe(H_2O)_6]^{3+}$ | 8.7545 | 8.7500 |

**Table S3.** Standard Gibbs free energy of formation ($\Delta G_f^{\circ}$, kcal/mol) and formation constant ($K_f$, $logK_f$) for the calculated complexes of Cu(II) and Fe(III) with $ASC^-$ with unusually low coordination numbers in aqueous solution at 298.15 K.

| COMPLEX $[\boldsymbol{Cu(ASC)_x(H_2O)_n}]^{(2-x)+}$ | $\Delta \boldsymbol{G}^{\circ}_{\boldsymbol{f}_{Cu^{2+}-ASC^-}}$ | $\boldsymbol{K}_{\boldsymbol{f}_{Cu^{2+}-ASC^-}}$ | $\boldsymbol{logK}_{\boldsymbol{f}_{Cu^{2+}-ASC^-}}$ |
|---|---|---|---|
| **{D1} $[Cu(ASC)(H_2O)_2]^+$ (3-coord.)** | -9.7 | $1.19 \times 10^{7}$ | 7.07 |
| **{D2] $[Cu(ASC)_2]$ (2-coord.)** | -17.8 | $1.07 \times 10^{13}$ | 13.03 |

| COMPLEX $[\boldsymbol{Fe(ASC)_x(H_2O)_n}]^{(3-x)+}$ | $\Delta \boldsymbol{G}^{\circ}_{\boldsymbol{f}_{Fe^{3+}-ASC^-}}$ | $\boldsymbol{K}_{\boldsymbol{f}_{Fe^{3+}-ASC^-}}$ | $\boldsymbol{logK}_{\boldsymbol{f}_{Fe^{3+}-ASC^-}}$ |
|---|---|---|---|
| **{D3} $[Fe(ASC)(H_2O)_2]^{2+}$ (3-coord.)** | -19.6 | $2.17 \times 10^{14}$ | 14.34 |
| **{D4] $[Fe(ASC)_2]^+$ (2-coord.)** | -27.9 | $2.92 \times 10^{20}$ | 20.47 |
| **{D5} $[Fe(ASC)_2(H_2O)]^+$ (3-coord.)** | -29.8 | $6.73 \times 10^{21}$ | 21.83 |
| **{D6] $[Fe(ASC)_3]$ (3-coord.)** | -53.6 | $1.99 \times 10^{39}$ | 39.30 |

**Table S4.** Standard Gibbs free energy of reaction (ΔG°, kcal/mol) and activation (ΔG≠, kcal/mol), various rate constants (k, $k_D$ and $k_{app}$, $M^{-1}\ s^{-1}$) and the rate constant ratio (using $k_{app}$ for the reduction of $[Cu(H_2O)_4]^{2+}$ as reference) for the initial reaction of the Haber-Weiss cycle (with and without iron complexation with PM) with $O_2^{\bullet-}$ in aqueous solution at 298.15 K.[a]

| **Reaction** | **ΔG⁰** | **ΔG≠** | **k** | **$k_D$** | **$k_{app}$** | **Ratio** |
|---|---|---|---|---|---|---|
| $[Cu(H_2O)_4]^{2+} + O_2^{\bullet-} \rightarrow [Cu(H_2O)_4]^{+} + O_2$ | -32.6 | 0.2 | $4.37 \times 10^{12}$ | $7.73 \times 10^{9}$ | $7.71 \times 10^{9}$ | |
| **{A9}**$[Cu(PM)(H_2O)_2]^+ + O_2^{\cdot-} \rightarrow$ **{E2}**$[Cu(PM)(H_2O)_2] + O_2$ | -16.0 | 3.5 | $1.77 \times 10^{10}$ | $8.04 \times 10^{9}$ | $5.53 \times 10^{9}$ | 1.39 |
| **{A11}**$[Cu(PM)_2] + O_2^{\cdot-} \rightarrow$ **{E3}**$[Cu(PM)_2]^- + O_2$ | -1.2 | 9.4 | $7.95 \times 10^{5}$ | | | $9.70 \times 10^{3}$ |

**Table S5.** Standard Gibbs free energy of reaction (ΔG°, kcal/mol) and activation (ΔG≠, kcal/mol), various rate constants (k, $k_D$ and $k_{app}$, $M^{-1}\ s^{-1}$) and the rate constant ratio (using $k_{app}$ for the reduction of $[Cu(H_2O)_4]^{2+}$ as reference) for the initial reaction of the Haber-Weiss cycle (with and without iron complexation with PM) with ascorbate ($ASC^-$) in aqueous solution at 298.15 K.[a]

| **Reaction** | **ΔG⁰** | **ΔG≠** | **k** | **$k_D$** | **$k_{app}$** | **Ratio** |
|---|---|---|---|---|---|---|
| $[Cu(H_2O)_4]^{2+} + ASC^- \rightarrow [Cu(H_2O)_4]^{+} + ASC^\bullet$ | -9.5 | 4.5 | $2.92 \times 10^{9}$ | $7.43 \times 10^{9}$ | $2.10 \times 10^{9}$ | |
| **{A9}**$[Cu(PM)(H_2O)_2]^+ + ASC^- \rightarrow$ **{E2}**$[Cu(PM)(H_2O)_2] + ASC^\bullet$ | 7.1 | 12.8 | $2.47 \times 10^{3}$ | | | $8.50 \times 10^{5}$ |
| **{A11}**$[Cu(PM)_2] + ASC^- \rightarrow$ **{E3}**$[Cu(PM)_2]^- + ASC^\bullet$ | 21.9 | 23.3 | $5.13 \times 10^{-5}$ | | | $4.09 \times 10^{13}$ |

**Table S6.** Standard Gibbs free energy of reaction (ΔG°, kcal/mol) and activation (ΔG≠, kcal/mol), various rate constants (k, $k_D$ and $k_{app}$, $M^{-1}$ $s^{-1}$) and the rate constant ratio (using $k_{app}$ for the reduction of $[Fe(H_2O)_6]^{3+}$ as reference) for the initial reaction of the Haber-Weiss cycle (with and without iron complexation with PM) with $O_2^{\bullet -}$ in aqueous solution at 298.15 K.[a]

| Reaction | ΔG⁰ | ΔG≠ | k | $k_D$ | $k_{app}$ | Ratio |
|---|---|---|---|---|---|---|
| $[Fe(H_2O)_6]^{3+} + O_2^{\bullet -} \rightarrow [Fe(H_2O)_6]^{2+} + O_2$ | -37.9 | 2.2 | $1.42 \times 10^{11}$ | $7.67 \times 10^{9}$ | $7.28 \times 10^{9}$ | |
| **{B9}**$[Fe(PM)(H_2O)_4]^{3+} + O_2^{\cdot -} \rightarrow$ **{E5}**$[Fe(PM)(H_2O)_4]^{2+} + O_2$ | -16.8 | 0.4 | $3.20 \times 10^{12}$ | $8.23 \times 10^{9}$ | $8.21 \times 10^{9}$ | 0.89 |
| **{B11}**$[Fe(PM)_2(H_2O)_2]^{3+} + O_2^{\cdot -} \rightarrow$ **{E6}**$[Fe(PM)_2(H_2O)_2]^{2+} + O_2$ | -11.2 | 3.0 | $3.89 \times 10^{10}$ | $8.68 \times 10^{9}$ | $7.10 \times 10^{9}$ | 1.02 |
| **{B12}**$[Fe(PM)_3]^{3+} + O_2^{\cdot -} \rightarrow$ **{E7}**$[Fe(PM)_3]^{2+} + O_2$ | -2.3 | 8.4 | $4.08 \times 10^{6}$ | | | $1.78 \times 10^{3}$ |

**Table S7.** Standard Gibbs free energy of reaction (ΔG°, kcal/mol) and activation (ΔG≠, kcal/mol), various rate constants (k, $k_D$ and $k_{app}$, $M^{-1}$ $s^{-1}$) and the rate constant ratio (using $k_{app}$ for the reduction of $[Fe(H_2O)_6]^{3+}$ as reference) for the initial reaction of the Haber-Weiss cycle (with and without iron complexation with PM) with ascorbate ($ASC^{-}$) in aqueous solution at 298.15 K.[a]

| Reaction | ΔG⁰ | ΔG≠ | k | $k_D$ | $k_{app}$ | Ratio |
|---|---|---|---|---|---|---|
| $[Fe(H_2O)_6]^{3+} + ASC^{-} \rightarrow [Fe(H_2O)_6]^{2+} + ASC^{\bullet}$ | -14.8 | 0.3 | $3.17 \times 10^{12}$ | $7.45 \times 10^{9}$ | $7.43 \times 10^{9}$ | |
| **{B9}**$[Fe(PM)(H_2O)_4]^{3+} + ASC^{-} \rightarrow$ **{E5}**$[Fe(PM)(H_2O)_4]^{2+} + ASC^{\bullet}$ | 6.3 | 8.4 | $3.99 \times 10^{6}$ | | | $1.86 \times 10^{3}$ |
| **{B11}**$[Fe(PM)_2(H_2O)_2]^{3+} + ASC^{-} \rightarrow$ **{E6}**$[Fe(PM)_2(H_2O)_2]^{2+} + ASC^{\bullet}$ | 11.9 | 13.9 | $3.91 \times 10^{2}$ | | | $1.90 \times 10^{7}$ |
| **{B12}**$[Fe(PM)_3]^{3+} + ASC^{-} \rightarrow$ **{E7}**$[Fe(PM)_3]^{2+} + ASC^{\bullet}$ | 25.3 | 25.3 | $1.67 \times 10^{-6}$ | | | $4.45 \times 10^{15}$ |

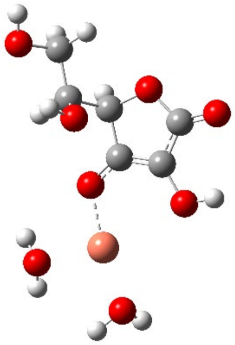

{D1} $[Cu(ASC)(H_2O)_2]^+$ (3-coord.)

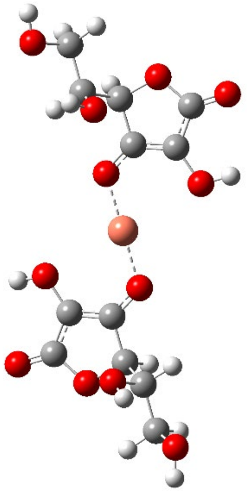

{D2} $[Cu(ASC)_2]$ (2-coord.)

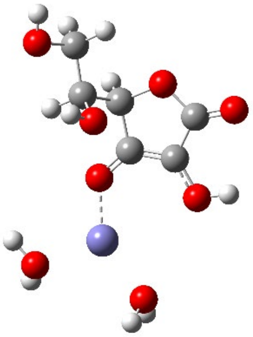

{D3} $[Fe(ASC)(H_2O)_2]^{2+}$ (3-coord.)

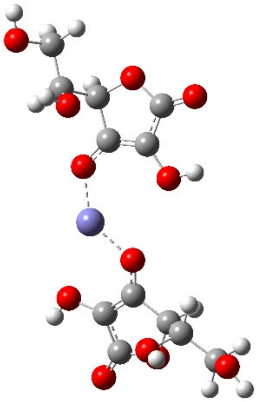

{D4} $[Fe(ASC)_2]^+$ (2-coord.)

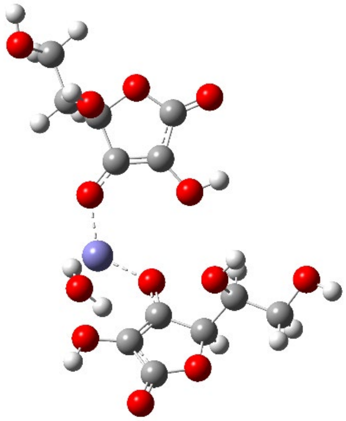

{D5} $[Fe(ASC)_2(H_2O)]^+$ (3-coord.)

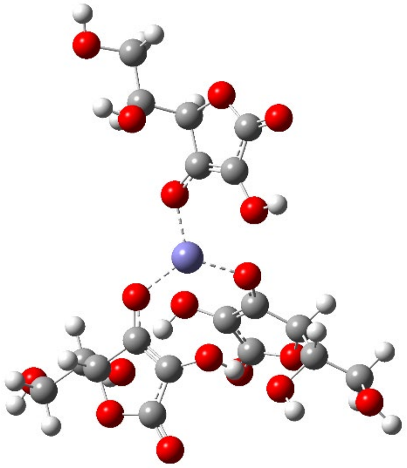

{D6} $[Fe(ASC)_3]$ (3-coord.)

**Figure S1.** Optimized geometries of the calculated complexes of Cu(II) and Fe(III) with $ASC^-$ with unusually low coordination numbers in aqueous solution (bond distances in Å).

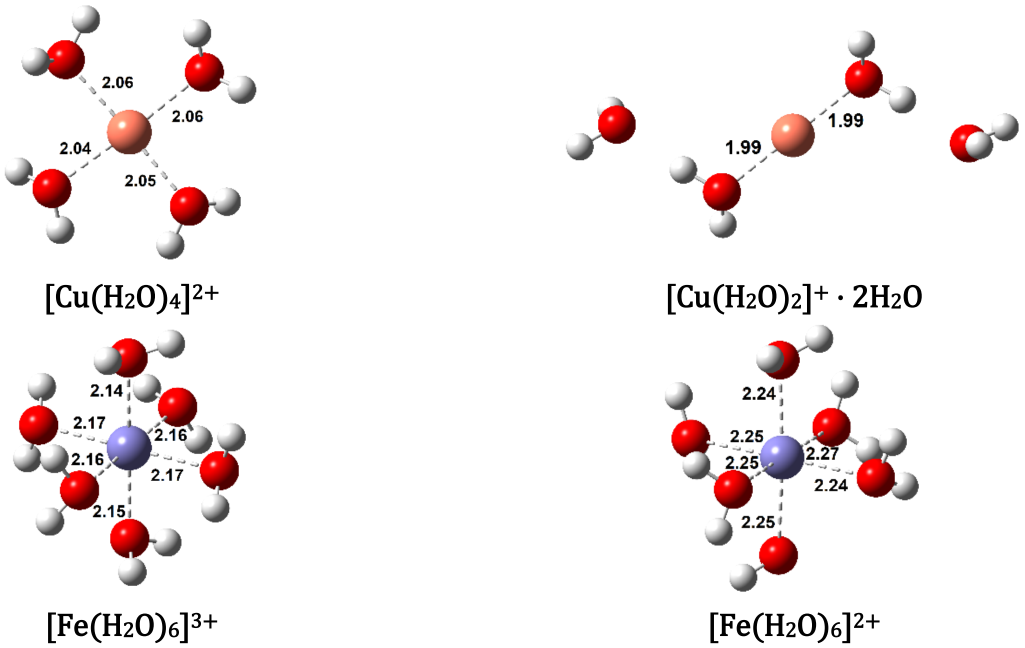


**Figure S2.** Optimized geometries of the most stable hydrated Cu(II), Cu(I), Fe(III) and Fe(II) complexes in aqueous solution (bond distances in Å); both iron complexes are high spin.

**Appendix 1**. Additional details regarding the calculation of the rate constants.

Using eq S1, the rate constant ($k$) was calculated following conventional transition state theory. The standard Gibbs free energy of activation ($\Delta G^{\neq}$) was estimated applying Marcus theory [34,35]. $\Delta G^{\neq}$ is also known as the single electron-transfer activation barrier ($\Delta G^{\neq}_{SET}$), which is calculated using eq S2. In this equation, $\Delta G^{0}_{SET}$ is the standard Gibbs free energy of the reaction and λ is the reorganization energy, which can be calculated with eq S3. In this formula, $\Delta E_{SET}$ is the nonadiabatic difference between the single-point energy calculations of reactants and vertical products.

$$k = \frac{k_B T}{h} e^{-\frac{\Delta G^{\neq}}{RT}} \tag{S1}$$

$$\Delta G^{\neq}_{SET} = \frac{\lambda}{4}(1 + \frac{\Delta G^{0}_{SET}}{\lambda})^2 \tag{S2}$$

$$\lambda = \Delta E_{SET} - \Delta G^{0}_{SET} \tag{S3}$$

When the value of $k$ was above $10^8$ $M^{-1}$ $s^{-1}$ (in the diffuse-limited regime), eq S4 was employed to calculate $k_{app}$ (the apparent rate constant), following the Kimball-Collins theory [36].

$$k_{app} = \frac{k_D\, k}{k_D + k} \tag{S4}$$

$k_D$ is the steady-state Smoluchowski rate constant for an irreversible diffusion-controlled bimolecular reaction, which can be calculated using eq S5 [37].

$$k_D = 4\pi R D_{AB} N_A = \frac{2 N_A k_B T}{3\eta} \cdot \frac{(a_A + a_B)^2}{a_A\, a_B} \tag{S5}$$

In eq S5, $R$ is the reaction distance (taken as the sum of the radii of the two reactants assuming a spherical shape), $N_A$ is Avogadro's constant, and $D_{AB}$ is the mutual diffusion coefficient of the reactants, computed as the sum of $D_A$ and $D_B$. The Stokes-Einstein approach can be employed to determine these values, as shown in eq S6 [38,39]. In this equation, $k_B$ is the Boltzmann constant; $T$ is the absolute temperature; $\eta$, the viscosity of the solvent (8.91 x $10^{-4}$ Pa s for water); and $\alpha_{A\ or\ B}$ is the radius of the solute (A or B), assuming it is spherical.

$$D_{A\ or\ B} = \frac{k_B T}{6\pi\eta a_{A\ or\ B}} \tag{S6}$$

**Appendix 2.** Additional explanation on the $pK_a$ calculation for the neutral (zwitterion) model Amadori compound.

In a previous publication [46], Brown and Mora-Diez showed that the $pK_a$ of a chemical species can be found with greater accuracy relative to a reference acid of similar structure whose $pK_a$ value is known. They used this methodology to determine the $pK_a$ of various protonated benzimidazoles ($HBz^+$), employing eq S7, in which $HBz_1^+$ is the reference acid of known $pK_a$:

$$HBz^+ + Bz_1 \leftrightarrows Bz + HBz_1^+ \qquad (S7)$$

Using this approach, L-alanine ($pK_a$ (HAla) = 9.87) was selected as reference acid, given its structural similarity to the model Amadori compound studied, following the equilibrium shown in eq S8.

$$HAMD + Ala^- \leftrightarrows AMD^- + HAla \qquad (S8)$$

HAMD and $AMD^-$ refer to the neutral and deprotonated Amadori compound respectively. HAMD, the most stable form at physiological pH, is a zwitterion, and therefore the proton is present in the amine group, and not the carboxylic group. Optimizing the structures of both the zwitterion and the neutral molecule in aqueous solution showed that the zwitterion is much more stable, thus confirming this. $AMD^-$ is the species that forms the most stable complexes with Cu(II) and Fe(III). Finally, HAla is the zwitterionic L-alanine, whereas $Ala^-$ is the deprotonated molecule. Consequently, the proton is migrating from one amine group to the other.

According to this approach, the unknown $pK_a$ can be obtained using eq S9, where $pK$ (calculated using eq S10) refers to the equilibrium constant of the system shown in eq S8.

$$pK_a\,(HAMD) = pK + pK_a\,(HAla) \qquad (S9)$$

$$pK = \frac{\Delta G^{\circ}_{aq}}{RTln10} \qquad (S10)$$

$\Delta G^{\circ}_{aq}$ can be calculated from the difference in $G^{\circ}_{aq}$ values of the products and reactants, whose structures have been optimized including solvent effects. Our calculated $G^{\circ}_{aq}$ values (in au) are shown below.

| | |
|---|---|
| HAMD | -590.623503 |
| $Ala^-$ | -323.145266 |
| $AMD^-$ | -590.172764 |
| HAla | -323.597817 |

$\Delta G^{\circ}_{aq}$ = -1.812 x $10^{-3}$ hartree = -4757.4 J/mol

$pK$ = -0.83346

$pK_a$ (HAMD) = 9.04

This value is closely related to the experimental aqueous $pK_a$ value of L-alanine, which is structurally very similar to the model used to study the Amadori compound.

**Appendix 3**. Additional explanation on the pK calculation for the equilibrium between protonated pyridoxamine $H_2PM^+$ and the anionic form $PM^-$.

At physiological pH, the most stable form of PM is the protonated zwitterion (labelled $H_2PM(\pm)^+$ in Scheme S11, which was taken from the paper by Casasnovas *et al.* [9]), but the species that forms the most stable complexes with Cu(II) and Fe(III) is the anionic form (labelled $PM(-)^-$ in Scheme S11, and $PM^-$ in our paper (see Figure 1)). To calculate the pK for the equilibrium between $H_2PM(\pm)^+$ and $PM(-)^-$, needed to estimate the Gibbs free energy cost in forming $PM(-)^-$ at physiological pH, the information of Scheme S11 was used. The structures of the tautomers $H_2PM^+$ and HPM were optimized considering all the possible conformations. It was found that $H_2PM(\pm)^+$ and HPM(0) are the most stable tautomers at the M05(SMD)/6-311+G(d,p) level of theory. Thus, we followed the path that goes from $H_2PM(\pm)^+$ to $PM(-)^-$ through HPM(0). In order to be consistent, we made use of the experimental $pK_a$ values reported by Vilanova *et al* (indicated as the superscript a in Scheme S11, being b calculated $pK_a$ values taken from another reference) [52]. Note that the value shown to go from $H_3PM(+)^{2+}$ to $H_2PM(\pm)^+$, 3.51, is the reverse of what is needed (-3.51). The resulting path, which includes the aqueous $pK_a$ values used for each acid dissociation, is represented in Scheme S12.

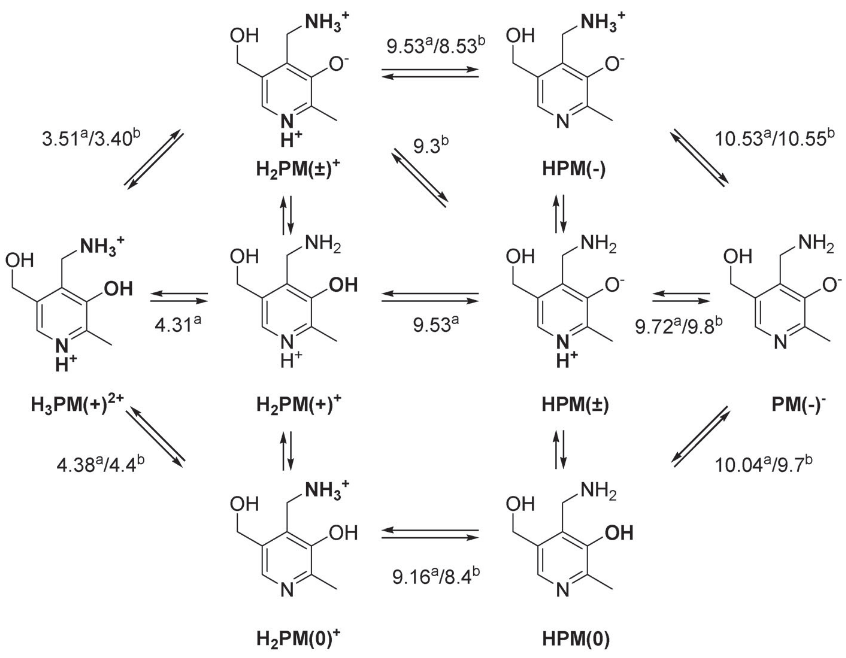


(S11)

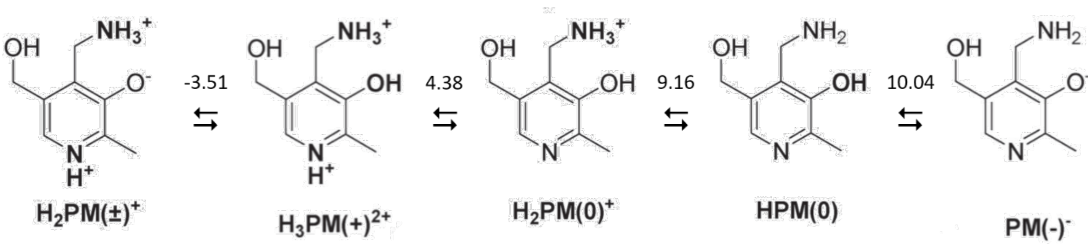


(S12)

The pK for the equilibrium between $H_2PM(\pm)^+$ and $PM(-)^-$ was calculated to be 20.07, which leads to a calculated deprotonation energy (using eq 21) of 7.2 kcal/mol.

M05(SMD)/6-311+G(d,p) Cartesian coordinates of the optimized geometries in water of the species calculated in this study.

## {A1} $[Cu(AMD)(H_2O)_2]^+$ (k, OH)

```
Charge = 1 Multiplicity = 2
N,0,-3.1537218233,-0.9817207848,3.0721358057
H,0,-4.0810813358,-0.6865779866,2.787342383
C,0,-3.2376641184,-1.4996493688,4.4395023714
H,0,-2.2254440892,-1.7836892432,4.7379659374
C,0,-4.1557629948,-2.7097643538,4.6175138503
H,0,-3.8088248379,-3.5681420755,4.0371262011
H,0,-4.1857879644,-3.0159708472,5.6665931069
H,0,-5.1761699739,-2.4711020512,4.3008738086
C,0,-3.7048089498,-0.3915408344,5.3936019699
O,0,-3.1946331745,-0.3813189418,6.544804613
O,0,-4.590199301,0.4015602233,4.9835103283
C,0,-2.63750476,-1.9351944701,2.1232797379
H,0,-3.2676357621,-2.8302413733,1.9668047257
H,0,-1.6700329251,-2.3188633223,2.4597138728
C,0,-2.4685572297,-1.3871300787,0.7526623919
O,0,-3.0942751029,-0.4021436081,0.3606124846
C,0,-1.5185761744,-2.0845057496,-0.1658443766
H,0,-0.5188349987,-2.038814823,0.2763763668
H,0,-1.7978266114,-3.1366758581,-0.2561908269
O,0,-1.5028088528,-1.4396357605,-1.4299505717
H,0,-1.719442953,-2.0715284407,-2.124324259
Cu,0,-2.8443237949,0.1860930118,-1.5519521732
O,0,-2.6011718646,0.4820759149,-3.530842207
H,0,-1.686365225,0.3034199676,-3.7804457223
H,0,-2.7504463345,1.4088495606,-3.7550629388
O,0,-3.9863977225,1.8766653165,-1.3020431284
H,0,-3.5893624238,2.609906689,-1.7865460151
H,0,-3.9546013618,2.1365470582,-0.3736307262
```

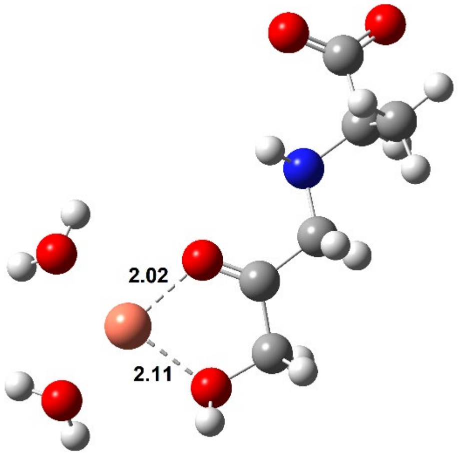


## {A2} $[Cu(AMD)(H_2O)_2]^+$ (N, k)

```
Charge = 1 Multiplicity = 2
Cu,0,-1.2502127787,0.3597371804,0.8652561809
N,0,0.7882288991,0.4918596419,0.9762261778
H,0,1.0292285129,0.9419422234,1.8544036381
C,0,1.3379710986,1.3712719741,-0.0903745486
C,0,2.8112321307,1.7504262807,0.2153394049
O,0,3.5006776938,2.1615726246,-0.7456234919
O,0,3.1771470624,1.6687627291,1.4129570077
C,0,1.3620894024,-0.8635949716,1.0040238485
H,0,2.3152413462,-0.9299735132,0.4764728861
H,0,1.5666547478,-1.1604513372,2.0380526785
C,0,0.395928523,-1.8594400546,0.4626839234
O,0,-0.7792800955,-1.5465836738,0.2765124004
C,0,0.8228298393,-3.257119199,0.1625434458
H,0,0.1490365701,-3.9250217515,0.7128073538
H,0,0.6369651256,-3.4143500178,-0.9082249053
O,0,2.1720983772,-3.4673707467,0.501920437
H,0,2.4011754809,-4.3676843737,0.2558479707
H,0,0.7926463569,2.3166609581,0.0050197759
C,0,1.0933285212,0.8186198257,-1.479080075
H,0,1.4220434153,1.5442855953,-2.2221642773
H,0,1.6410876343,-0.1099626283,-1.6607285202
H,0,0.0290159963,0.6322166395,-1.6419388695
O,0,-3.2740009713,0.0374769508,0.7516991947
H,0,-3.4613826396,-0.6486717697,0.1006865983
H,0,-3.7373342448,0.8215998635,0.4349443379
O,0,-1.5668616181,2.2354338044,1.6729469174
H,0,-0.8959957272,2.8927477203,1.4569000542
```

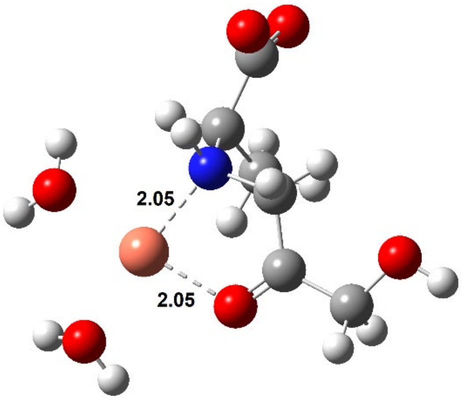

```
H,0,-2.3974281089,2.6060343154,1.3531289857
```

## {A3} [Cu(AMD)($H_2O$)$_2$]$^+$ (CO, N)

```
Charge = 1 Multiplicity = 2
N,0,-1.9845512246,-1.7642995559,-1.3748159981
H,0,-2.6265133299,-2.5094054269,-1.646503934
C,0,-1.6443783001,-1.9541489097,0.0568414959
H,0,-0.692755731,-1.4402847326,0.2275260351
C,0,-1.5093941468,-3.4197507392,0.4243724826
H,0,-0.7691275263,-3.9136874405,-0.2086835654
H,0,-1.1868247997,-3.5210203967,1.4609228626
H,0,-2.4670216467,-3.9354313386,0.3078846165
C,0,-2.6487780356,-1.2130687178,0.9384361631
O,0,-2.6747374021,-1.4320870655,2.1517950354
O,0,-3.4033547198,-0.3494795836,0.3619560613
C,0,-0.8384842336,-1.8333939624,-2.2822766304
H,0,-0.0651764485,-2.5104216115,-1.8960801619
H,0,-0.3711713293,-0.8524437266,-2.3903650231
C,0,-1.2220425655,-2.3903784046,-3.6235813247
O,0,-2.2308336203,-3.0491321017,-3.7877173401
C,0,-0.2901291171,-2.096856179,-4.7616399282
H,0,-0.3664630114,-1.0154125837,-4.9515393562
H,0,0.740866661,-2.2959056051,-4.4557074569
O,0,-0.5854973884,-2.8460023813,-5.9133328027
H,0,-1.5241486791,-3.0685588009,-5.8825557733
Cu,0,-3.1634586035,-0.0793168588,-1.5516602659
O,0,-2.8063792262,0.2372319647,-3.5566641091
H,0,-1.9220893803,0.5932925593,-3.7041127983
H,0,-3.4068440987,0.9063011702,-3.9049589003
O,0,-4.5278912885,1.4969300229,-1.455144493
H,0,-4.3305816186,2.1791681977,-2.1063687449
H,0,-4.4619335094,1.9344961177,-0.5990549962
```

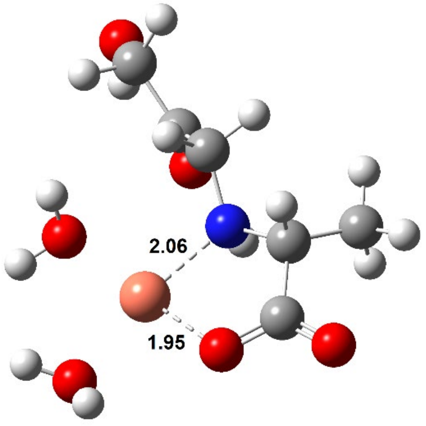


## {A4} [Cu(AMD)($H_2O$)]$^+$ (CO, N, k)

```
Charge = 1 Multiplicity = 2
Cu,0,-1.1719668924,0.2367813273,0.5333186425
N,0,0.790913262,0.3442220546,0.9256336938
H,0,0.8305574303,0.5718605517,1.9168342965
C,0,1.334288369,1.5004494295,0.1754771514
C,0,0.243500298,2.5757869196,0.0935459957
O,0,0.5478711942,3.7454752298,-0.1480276522
O,0,-0.9682473092,2.1725268705,0.2369838242
C,0,1.4490375733,-0.9483710733,0.7214866117
H,0,1.9482090033,-0.9750211606,-0.2525802374
H,0,2.2191759723,-1.14328541,1.470712678
C,0,0.4425488014,-2.0559466133,0.7208864488
O,0,-0.7629060429,-1.8166291059,0.7196170627
C,0,0.902859237,-3.476655374,0.705573941
H,0,0.5498804727,-3.9277526042,1.6423244302
H,0,0.3806636611,-3.9790938477,-0.1170969331
O,0,2.3020207839,-3.5610123832,0.5749822235
H,0,2.5486310135,-4.4883174262,0.6223082937
H,0,1.4704433127,1.162677742,-0.8575332574
C,0,2.6528018708,1.9970393287,0.7292510036
H,0,3.0275796567,2.8212965669,0.1214197347
H,0,2.5376515198,2.3504227773,1.7578293546
H,0,3.3995341579,1.2003427213,0.7148853889
O,0,-3.1968684455,0.19494632,0.2782897795
H,0,-3.4986582247,-0.6303344578,-0.1174879438
H,0,-3.4749816753,0.8889986168,-0.329819531
```

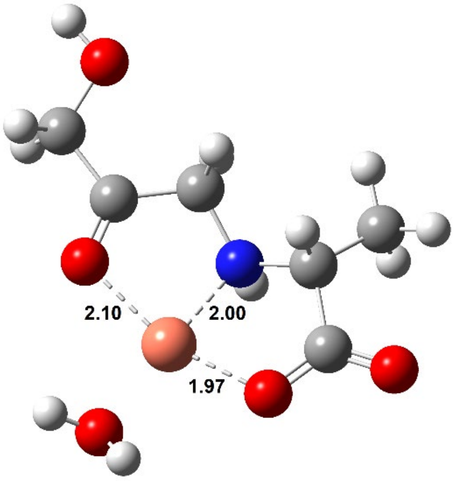

## {A5} [Cu(AMD)2] (CO, N; mirror)

```
Charge = 0 Multiplicity = 2
N,0,-3.5113316303,-0.3878426462,0.3074821017
H,0,-2.8451910195,0.3004259651,0.6566820911
C,0,-3.0107038615,-1.735004518,0.6782944943
H,0,-3.8825797038,-2.3972364551,0.7077421534
C,0,-2.3263936671,-1.7390508436,2.033017001
H,0,-2.9879332776,-1.3375123828,2.8040135917
H,0,-2.0556336162,-2.755580154,2.3193353211
H,0,-1.4158363995,-1.1332886103,2.0088273703
C,0,-2.1376810746,-2.314790808,-0.4332781582
O,0,-1.4522661177,-3.3179711813,-0.2009479697
O,0,-2.2030061338,-1.7490064983,-1.5777276559
C,0,-4.8042398218,-0.0775510074,0.9243547873
H,0,-4.913701858,-0.5787262293,1.8953579984
H,0,-5.6238138303,-0.4377684191,0.2991285435
C,0,-4.9733632015,1.3908349738,1.2076247052
O,0,-4.0419521666,2.1685071462,1.142060802
C,0,-6.3681696368,1.8237795851,1.5890469244
H,0,-7.0609846728,1.4635327216,0.8174375131
H,0,-6.6388097569,1.3111052797,2.5173081112
O,0,-6.4942515543,3.2062236463,1.8021148774
H,0,-6.3748050182,3.6510171034,0.9543587278
N,0,-4.2206565745,1.6797342099,-2.1643809646
H,0,-4.0887949435,2.320693566,-1.3862962627
C,0,-3.480392512,2.2275950328,-3.3314909031
C,0,-3.1749243562,1.0756159643,-4.2949188717
O,0,-3.0034813034,1.2953616216,-5.4996122845
O,0,-3.0625547971,-0.0776645143,-3.7525349588
C,0,-5.6645178179,1.5049943616,-2.354352356
H,0,-6.0122071889,0.650003761,-1.7629982291
H,0,-5.8999865929,1.2623314615,-3.3921899729
C,0,-6.4949423564,2.6701078005,-1.8861891617
O,0,-6.0347286708,3.5775459301,-1.2193358604
C,0,-7.9526578376,2.6394822405,-2.2416367934
H,0,-8.0148081961,2.819579063,-3.3252002273
H,0,-8.3561576679,1.639638368,-2.0595102441
O,0,-8.7030257424,3.5829777185,-1.5174215856
H,0,-8.0970350444,4.2797883308,-1.2354251387
Cu,0,-3.3224144779,-0.1406456616,-1.7631300128
C,0,-4.0980979661,3.4507483419,-3.9778374554
H,0,-5.0668879343,3.2365375744,-4.4362500998
H,0,-4.2237263923,4.2491469674,-3.2439964658
H,0,-3.4348328555,3.8177507841,-4.7620170977
H,0,-2.5001517432,2.5092904401,-2.9317363954
```

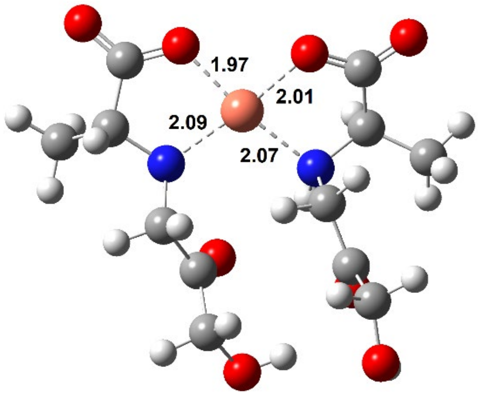


## {A6} [Cu(AMD)2] (CO, N)

```
Charge = 0 Multiplicity = 2
N,0,-2.0104500716,-1.7464995369,-1.4023994751
H,0,-2.6528423028,-2.5085569343,-1.6147489918
C,0,-1.7071975156,-1.801689717,0.0450958248
H,0,-0.8452931246,-1.1459144295,0.2122203483
C,0,-1.3863092233,-3.2023779235,0.5314643471
H,0,-0.5431209346,-3.6260749287,-0.0177974557
H,0,-1.1183665446,-3.1824933594,1.5881533437
H,0,-2.2493643984,-3.8625815672,0.4065968716
C,0,-2.8567897091,-1.148597266,0.8236892627
O,0,-2.9721446238,-1.3630909619,2.0349453456
O,0,-3.6292059574,-0.3665222467,0.1645313181
C,0,-0.8563853748,-1.886035614,-2.2857019308
H,0,-0.1470501197,-2.6392611453,-1.9165328724
H,0,-0.3110966895,-0.9430020018,-2.354073976
C,0,-1.2637260003,-2.3600442248,-3.6508279514
O,0,-2.3149653301,-2.9387358451,-3.8522887868
```

```
C,0,-0.2990429477,-2.1073563938,-4.7718033368
H,0,-0.2818222784,-1.0198338806,-4.9306652744
H,0,0.7078890637,-2.4049657281,-4.4639458691
O,0,-0.6522198552,-2.7932297657,-5.9477957418
H,0,-1.592269467,-3.0060041749,-5.883525858
N,0,-4.2434916189,1.6811409423,-1.9800533458
H,0,-4.2307555707,2.220722412,-1.1190143641
C,0,-3.5545199437,2.459992156,-3.0415229881
C,0,-2.9164157135,1.4867739767,-4.0418109857
O,0,-2.6664945148,1.8516250289,-5.19560091
O,0,-2.6111207697,0.3304212339,-3.5830310443
C,0,-5.6298699556,1.2956279274,-2.2460269179
H,0,-5.8753030062,0.4041851161,-1.6564050104
H,0,-5.7596243255,1.0149981159,-3.2924327035
C,0,-6.6494701074,2.3265312034,-1.8343091998
O,0,-6.4005011316,3.208544722,-1.0351530986
C,0,-8.0194935656,2.1985324988,-2.4318961634
H,0,-7.9156960923,2.4022036677,-3.5071792388
H,0,-8.3634734132,1.1638948392,-2.3327391193
O,0,-8.9466434302,3.071968229,-1.8359811229
H,0,-8.4390182537,3.7189446526,-1.3282285386
Cu,0,-3.1223680208,-0.0324505346,-1.7123812678
C,0,-4.3639856883,3.5705497857,-3.682515732
H,0,-5.2273674888,3.190608821,-4.2350197272
H,0,-4.7093423491,4.2796089295,-2.9282582749
H,0,-3.7331917561,4.1128836395,-4.387459688
H,0,-2.6942889693,2.9128306021,-2.5364575808
```

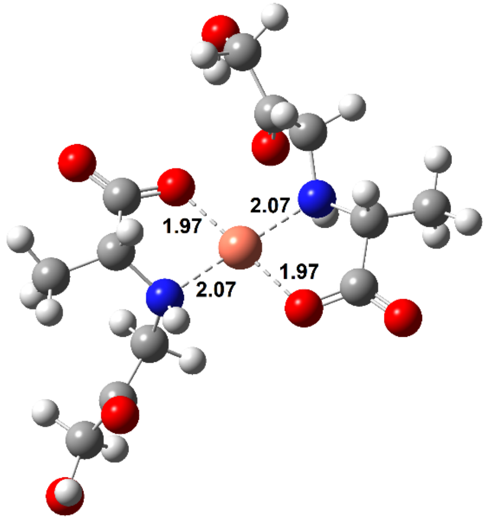


## {A7} $[Cu(ASC)(H_2O)_3]^+$

```
Charge = 1 Multiplicity = 2
C,0,-2.4756221753,1.7486004343,-1.7082674735
O,0,-1.5548026128,2.4323348215,-2.4397366281
C,0,-0.2873524954,2.3933365464,-1.7646135392
C,0,-0.534576524,1.5631882593,-0.5234090839
C,0,-1.8530693611,1.2162163841,-0.5376265027
O,0,-2.5079963158,0.4875464356,0.4108641972
H,0,-3.4489257139,0.478671815,0.2001597827
O,0,0.3391642184,1.3355033094,0.3911435867
O,0,-3.6372170984,1.6611239515,-2.077581429
H,0,-0.0448712245,3.4176427137,-1.4649797823
C,0,0.8007615249,1.8985540553,-2.6988012625
H,0,1.743763784,1.9593665183,-2.1384994808
C,0,0.9424944996,2.7808189486,-3.9223134194
H,0,0.0382446312,2.7301089621,-4.5359182311
H,0,1.0971485163,3.8185143896,-3.6047082846
O,0,2.0633490235,2.3104695639,-4.6593380953
H,0,2.0078133179,2.660417659,-5.5516290246
O,0,0.5354569312,0.5523150759,-3.0507471032
H,0,1.1858495439,0.292915567,-3.7107584349
Cu,0,2.2002793326,0.8235081003,0.087428197
O,0,2.7411691499,2.8432526331,0.3231918179
H,0,3.5977650971,2.9300151459,0.7569933536
H,0,2.1104217488,3.2555047145,0.9243005497
O,0,4.1541171349,0.2784920581,-0.2395863265
H,0,4.7600501968,0.973029748,0.042723049
H,0,4.3912970705,-0.4948310995,0.2851992373
O,0,1.6290983898,-1.1930888173,-0.1437363777
H,0,2.3398341586,-1.7845618942,0.128589121
H,0,0.9009845411,-1.3796323394,0.4597864872
```

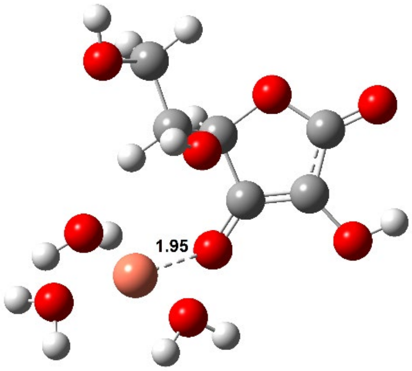

## {A8} $[Cu(ASC)_2(H_2O)_2]$

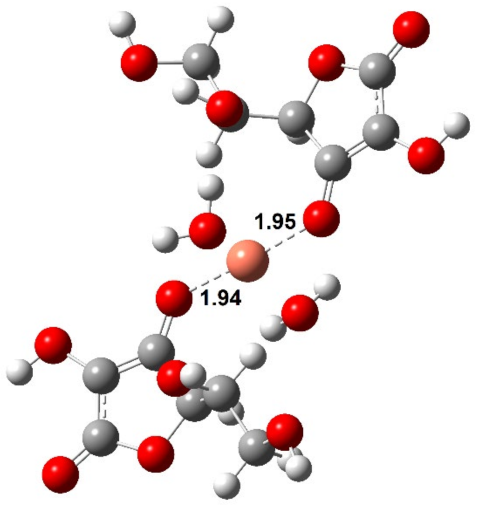


```
Charge = 0 Multiplicity = 2
C,0,-2.2623692803,1.3085326253,-1.3015491602
O,0,-1.4264772902,2.1893824013,-1.9226472521
C,0,-0.0957128379,2.0192375761,-1.4123043923
C,0,-0.2106489981,0.9070331759,-0.3936176316
C,0,-1.5221176051,0.528314734,-0.370142657
O,0,-2.0622584195,-0.4426534952,0.4293454871
H,0,-3.0003257134,-0.5282269202,0.2245678205
O,0,0.750974186,0.4568976305,0.3234355486
O,0,-3.454885415,1.2781942531,-1.5768059926
H,0,0.184373533,2.949366871,-0.9086466079
C,0,0.8815618133,1.7736407805,-2.5477395405
H,0,1.8833240342,1.7285165293,-2.100126358
C,0,0.881381636,2.9122568811,-3.5470950484
H,0,-0.0869312557,2.9756972481,-4.0524268901
H,0,1.0673285046,3.8554136346,-3.0201583437
O,0,1.911640846,2.6539113911,-4.4910860144
H,0,1.7804882216,3.2316825493,-5.2467518767
O,0,0.576485318,0.5348281576,-3.1637124608
H,0,1.161916862,0.4382518131,-3.921156696
Cu,0,2.6584698152,0.7759736768,0.1352639545
O,0,2.4230617074,2.8352159803,0.8134601319
H,0,3.0887811756,3.0271020538,1.4827846369
H,0,1.5785485741,2.9051601175,1.2720307914
O,0,2.9171969042,-0.9291160996,-1.1497795948
H,0,3.542827362,-1.5063096951,-0.6707222151
H,0,2.0858027267,-1.4139909128,-1.1747972406
C,0,7.4736648791,-0.2165787302,1.6698705736
O,0,6.5256925462,-0.8148289197,2.4484622365
C,0,5.2236991089,-0.3905244707,2.0200991706
C,0,5.4810993805,0.4970818076,0.8208968932
C,0,6.8370102027,0.5820353272,0.6806798275
O,0,7.4985657251,1.3162433237,-0.2659071626
H,0,8.4434785267,1.2836082409,-0.0788843424
O,0,4.5835551767,1.0869761356,0.1241306058
O,0,8.6648747382,-0.4038586168,1.8812268201
H,0,4.7982032022,0.2142967925,2.8275571371
C,0,4.3055598941,-1.5778754681,1.7970955868
H,0,3.2917931171,-1.1712298415,1.6828136081
C,0,4.2920481527,-2.5163869517,2.9850439961
H,0,5.2826642822,-2.9532980583,3.1365497057
H,0,4.010256916,-1.9559113053,3.8835965624
O,0,3.3442953752,-3.5382552048,2.7081018877
H,0,3.530318281,-4.2867720789,3.2801785162
O,0,4.6745338454,-2.2589430527,0.6038385325
H,0,4.3117284349,-3.1509066664,0.6556099073
```

## {A9} $[Cu(PM)(H_2O)_2]^+$

```
Charge = 1 Multiplicity = 2
C,0,-1.4593384046,-0.9033852423,-0.2300585128
C,0,-0.0869310807,-0.8819568577,-0.0200821177
C,0,0.5077402319,0.3394379172,0.3277230159
C,0,-0.3047565762,1.4858842134,0.4371749801
C,0,-1.701336242,1.336962857,0.2113269307
N,0,-2.2483511215,0.1688200104,-0.1151548753
H,0,-1.9458169595,-1.8344306248,-0.5110961677
C,0,-2.5936779214,2.5259218468,0.3618385529
H,0,-2.5442474587,2.9304741198,1.3780304938
H,0,-2.2938096194,3.3353036258,-0.3113871681
H,0,-3.6274456279,2.255688745,0.1455793091
C,0,0.6915588362,-2.1560198861,-0.1586904869
H,0,0.0715149762,-2.9066778131,-0.6584847686
```

H,0,1.5848364873,-1.9997931793,-0.7740973187
O,0,1.0730207062,-2.6268362617,1.1360280041
H,0,1.5777892455,-3.4359553227,1.0142201144
C,0,1.9726146109,0.4638120129,0.6219739288
H,0,2.1171506338,0.7284930503,1.6731518775
H,0,2.491942556,-0.4791861102,0.4530767656
N,0,2.6083549179,1.5231574808,-0.1861579315
H,0,3.6051561061,1.5348537508,0.0089680982
H,0,2.5135955812,1.2996048577,-1.1730472414
O,0,0.1644898282,2.6725588075,0.7652237508
Cu,0,1.8725771733,3.3718123837,0.1765693061
O,0,3.6366165157,4.2597931167,-0.5294915492
H,0,4.4337547933,3.8088708764,-0.2295829927
H,0,3.7208029321,5.1594656636,-0.1937195589
O,0,1.1079593922,5.2991850694,0.5778866276
H,0,0.3907245261,5.2130914792,1.2156645758
H,0,1.7537088317,5.8780514835,0.9971922183

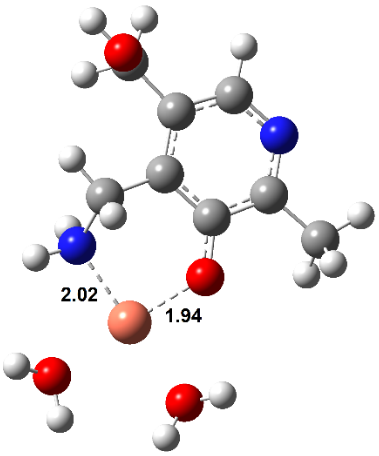


**{A10} [Cu(PM)$_2$]** (mirror image)
Charge = 0 Multiplicity = 2
C,0,-1.3427862929,-0.9141607473,-0.2443799402
C,0,-0.0741525724,-0.7962477044,0.3069616379
C,0,0.4707652863,0.4891704175,0.4521666466
C,0,-0.2910029798,1.6041259796,0.0387675582
C,0,-1.57799338,1.3519373764,-0.5229879047
N,0,-2.0762523554,0.1259945151,-0.654446187
H,0,-1.7867837486,-1.8991785088,-0.3682830667
C,0,-2.4080336375,2.5096544864,-0.975548651
H,0,-2.6205657091,3.1950527908,-0.1484268072
H,0,-1.8863250899,3.0975273219,-1.7378633655
H,0,-3.3541671506,2.1608204511,-1.3903304418
C,0,0.642990432,-2.0395013938,0.7409565989
H,0,0.1231634354,-2.915727528,0.3409884986
H,0,1.6678059882,-2.0564625881,0.3524484479
O,0,0.6680892507,-2.1033451258,2.1697442819
H,0,1.1645702604,-2.88833082,2.4177632447
C,0,1.8221233445,0.7149373927,1.0630866945
H,0,1.7146702034,1.1927810328,2.0417528191
H,0,2.3400076198,-0.2301826343,1.2255448377
N,0,2.6637528328,1.6007171635,0.2312218085
H,0,3.6096099647,1.5885276034,0.5988642868
H,0,2.7237805491,1.2164702182,-0.7073495115
O,0,0.1109007256,2.8448982938,0.1641712561
Cu,0,1.9734407968,3.5219780929,0.1797039541
C,0,3.0152780429,8.864813598,1.0762199587
C,0,3.7857660368,7.9035515081,0.4365490001
C,0,3.1783982844,6.6838650518,0.1042608616
C,0,1.8253602575,6.473929998,0.447271074
C,0,1.1377681423,7.5413485141,1.0971526041
N,0,1.7261470821,8.6973728439,1.39203275
H,0,3.459798727,9.8203403231,1.3450247038
C,0,-0.3026639383,7.3643638364,1.4547101174
H,0,-0.9137305718,7.1805156692,0.5647630704
H,0,-0.4442692616,6.4990038552,2.1103176042
H,0,-0.6811950288,8.252900942,1.9607424167
C,0,5.2244727447,8.2061319382,0.1378039039
H,0,5.4627013271,7.9849937698,-0.9083552783
H,0,5.4120846899,9.2719952267,0.3013011151
O,0,6.0629562625,7.4297332467,0.9981655845
H,0,6.9753150372,7.6036808488,0.7506368936
C,0,3.90331945,5.5950765432,-0.628111261
H,0,3.4252066753,5.418780166,-1.5963336806
H,0,4.9395865142,5.8699671472,-0.8214639989
N,0,3.8563853939,4.3188847714,0.1166664357
H,0,4.4822754248,3.6561945811,-0.3300740565

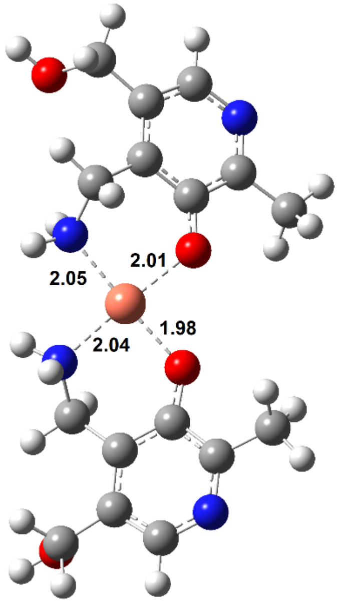

H,0,4.2280170096,4.4651519076,1.0510357202
O,0,1.1816253749,5.365689778,0.1735607158

## {A11} [Cu(PM)$_2$]

Charge = 0 Multiplicity = 2
C,0,-1.073606502,-0.829877173,-0.7213518202
C,0,0.1584460114,-0.7312883823,-0.0878302581
C,0,0.5399419607,0.515608788,0.4300077823
C,0,-0.3356968733,1.6128825667,0.278200555
C,0,-1.5697446891,1.3868365463,-0.3973266775
N,0,-1.9176345335,0.1950897949,-0.8763462667
H,0,-1.3915486586,-1.7850719003,-1.1328964085
C,0,-2.5173688975,2.5295574059,-0.5715991285
H,0,-2.8344836296,2.9344007196,0.3952752393
H,0,-2.0447873957,3.3559587474,-1.1125386808
H,0,-3.4032669244,2.2118490161,-1.1219052006
C,0,1.0111780942,-1.9596354794,0.027326518
H,0,0.6261483515,-2.7301254481,-0.6482167809
H,0,2.0445539003,-1.7467287989,-0.2689133999
O,0,0.9826030265,-2.4390760187,1.3738393562
H,0,1.5645179773,-3.2025657867,1.4242101365
C,0,1.8285897263,0.7162670598,1.1735502403
H,0,1.6138570499,0.9633114851,2.217681261
H,0,2.4271489998,-0.1947996508,1.1777955707
N,0,2.6175102748,1.8312518557,0.6146434734
H,0,3.5036232318,1.8981580922,1.1043196116
H,0,2.8448420483,1.6272462659,-0.3541216651
O,0,-0.07692437,2.8113079362,0.747761574
Cu,0,1.7222904806,3.6520639499,0.7765282909
C,0,4.5054369409,8.2972339824,-0.0012695818
C,0,3.2919272354,8.0960966185,0.6431417439
C,0,2.9266694544,6.7820281227,0.9688909089
C,0,3.7935141598,5.7246666296,0.6195630121
C,0,5.0133172019,6.0581521234,-0.0370556177
N,0,5.3491526104,7.312516076,-0.3281368307
H,0,4.8130937186,9.3061632746,-0.2663319671
C,0,5.9617067222,4.9632561469,-0.4058455911
H,0,6.3167977923,4.4296648214,0.4821908603
H,0,5.4767289405,4.2164257256,-1.0426834822
H,0,6.8242742382,5.3675002748,-0.9361737577
C,0,2.4311549427,9.284199158,0.9553914658
H,0,2.112122927,9.2726061631,2.0032473219
H,0,3.009235047,10.2001914444,0.7972250046
O,0,1.283653489,9.2811091571,0.1015322631
H,0,0.7249061486,10.0199150905,0.3593851058
C,0,1.6603176059,6.4493513147,1.7021059486
H,0,1.9063155707,5.9824882911,2.6605686469
H,0,1.077841385,7.3451074239,1.9155116756
N,0,0.8391051784,5.48170211,0.9471784133
H,0,-0.0425733526,5.3368065448,1.4280814991
H,0,0.6066012336,5.8769566989,0.0408738841
O,0,3.5330484305,4.4673804462,0.894627772

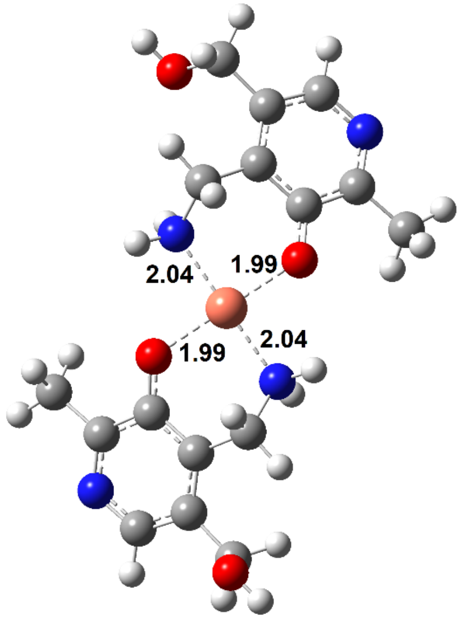


## {B1} [Fe(AMD)($H_2O$)$_4$]$^{2+}$

Charge = 2 Multiplicity = 6
N,0,-1.946932449,-1.9277545843,-1.2795656761
H,0,-2.561788586,-2.7064732599,-1.5145104898
C,0,-1.583857446,-2.023006076,0.147939057
H,0,-0.7245759591,-1.3612341023,0.3025791284
C,0,-1.2303581705,-3.4318243087,0.5834555196
H,0,-0.4011425423,-3.8238785032,-0.00853414
H,0,-0.9229430227,-3.4325219146,1.6294826245
H,0,-2.0881443198,-4.1001227369,0.471271465
C,0,-2.7018835028,-1.4026853184,0.9798821471

```
O,0,-2.7961016074,-1.6178406404,2.1812372634
O,0,-3.5012812227,-0.6117947134,0.3369704306
C,0,-0.8019011151,-1.957311597,-2.1904740473
H,0,-0.0500725427,-2.6890497744,-1.8650039216
H,0,-0.304400196,-0.9864663882,-2.203368171
C,0,-1.186804404,-2.378447169,-3.5782521301
O,0,-2.1999420931,-3.0077746977,-3.8137273388
C,0,-0.2489554708,-1.9719025727,-4.6773694423
H,0,-0.3447427492,-0.8778561148,-4.7703099638
H,0,0.7840367385,-2.1750277841,-4.3816769584
O,0,-0.5199590098,-2.620752874,-5.8934155312
H,0,-1.4560537936,-2.8558663911,-5.8951319498
O,0,-3.1837071256,0.1124888161,-3.7459013731
H,0,-2.2896908529,0.328445616,-4.0378052456
H,0,-3.7197773975,0.8771045568,-3.9906049412
O,0,-4.5422578751,1.6315351856,-1.3632585797
H,0,-4.2984656854,2.2764649922,-2.0380179046
H,0,-4.3987617909,2.0754065988,-0.5189601787
Fe,0,-3.2716979836,-0.1331469615,-1.5514897964
O,0,-1.51077117,1.0375440661,-1.2675348201
H,0,-1.3246780689,1.5372131225,-2.0727159803
H,0,-1.688354522,1.701575334,-0.5890685743
O,0,-4.9195890608,-1.5017989263,-1.8867649733
H,0,-5.3525656793,-1.371577168,-2.7387526287
H,0,-5.6042481141,-1.371844371,-1.2197293293
```

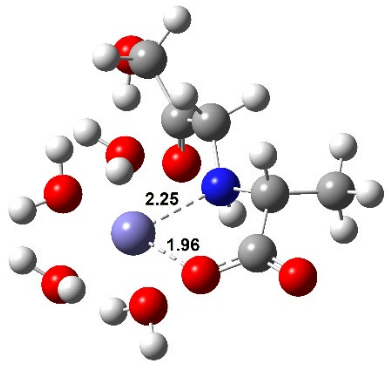


## {B2} $[Fe(AMD)(H_2O)_3]^{2+}$

```
Charge = 2 Multiplicity = 6
N,0,1.0038089496,0.3234568406,0.9282178306
H,0,1.0528031487,0.5460869421,1.9202553569
C,0,1.5110608793,1.4673596234,0.1600087769
C,0,0.4165861201,2.5323487088,0.1252348423
O,0,0.6650982603,3.6958164677,-0.1559742937
O,0,-0.7864660175,2.107230816,0.3726207151
C,0,1.6367248711,-0.9653012665,0.6963310709
H,0,2.0724267731,-1.0125785579,-0.3078350029
H,0,2.4500181686,-1.1729313277,1.3961641117
C,0,0.6160803432,-2.0510556514,0.7711976222
O,0,-0.5806007097,-1.7778857202,0.8361673378
C,0,1.0261073082,-3.4844120288,0.7544861875
H,0,0.6639907592,-3.9242560094,1.6930218904
H,0,0.4759738878,-3.96418536,-0.0641336246
O,0,2.4185767278,-3.617236042,0.6088121647
H,0,2.6273118784,-4.5553045993,0.6049251542
H,0,1.6048304629,1.1336423598,-0.8799331178
C,0,2.8479138434,1.9871147516,0.6491726417
H,0,3.1836438418,2.8141922664,0.0225741238
H,0,2.7748760934,2.339914002,1.6815488589
H,0,3.6008737982,1.1979224761,0.6010393475
O,0,-3.3666758205,0.2374510872,0.0169042585
H,0,-3.6026592259,-0.6069095253,-0.3857904384
H,0,-3.4933377475,0.892175279,-0.680685369
Fe,0,-1.2767560417,0.2451498775,0.6096677497
O,0,-1.889870825,0.2656725925,2.6754365395
H,0,-1.1073365942,0.214648137,3.2385848998
H,0,-2.3016295851,1.1124951512,2.8875813958
O,0,-0.9041804423,-0.1724894994,-1.7074108786
H,0,-0.4076739559,0.582151113,-2.0438319973
H,0,-1.7789476297,-0.080107244,-2.1005791641
```

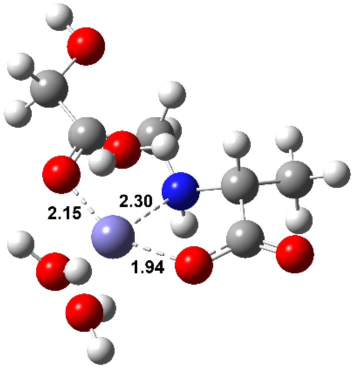

## {B3} $[Fe(AMD)_2(H_2O)_2]^+$

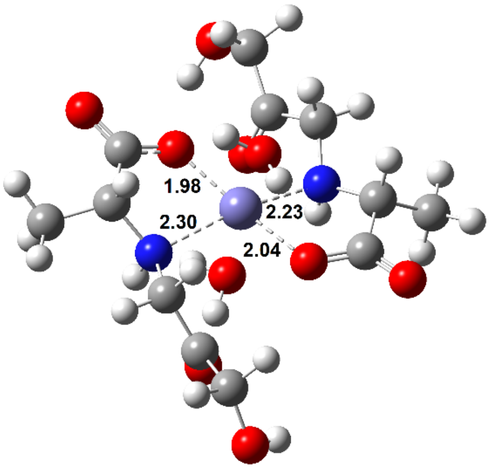


```
Charge = 1 Multiplicity = 6
N,0,-1.8454941428,-1.8993051269,-1.1049427572
H,0,-2.4591436817,-2.7093794935,-1.0492563273
C,0,-1.2578926975,-1.6516538385,0.225573444
H,0,-0.3797565265,-1.014325487,0.0743011377
C,0,-0.8428404637,-2.9301269654,0.9304755692
H,0,-0.128558156,-3.4933455534,0.3266381665
H,0,-0.3670598591,-2.698638196,1.8836811459
H,0,-1.7129522954,-3.5642984804,1.1227486697
C,0,-2.2195682353,-0.7968244566,1.0565677042
O,0,-2.0621611324,-0.7025987994,2.2741391256
O,0,-3.1383326141,-0.1753271691,0.4085986461
C,0,-0.8713847209,-2.1239589948,-2.1710874192
H,0,-0.0830142849,-2.8249744743,-1.8640172719
H,0,-0.373192431,-1.1848997154,-2.4253496306
C,0,-1.4968074216,-2.7267169186,-3.3970846248
O,0,-2.5810300949,-3.2761915818,-3.3773525534
C,0,-0.6998516732,-2.6163380485,-4.6662926188
H,0,-0.7316996289,-1.5551029767,-4.9561877461
H,0,0.346636839,-2.8642956798,-4.4724405498
O,0,-1.1773213522,-3.4519356614,-5.690787713
H,0,-2.1381056259,-3.4883697529,-5.6207099575
Fe,0,-3.2485542088,-0.2428869704,-1.6295721732
O,0,-1.843033844,1.4811941351,-1.4156123849
H,0,-2.0202998943,2.1415941686,-2.0965879257
H,0,-2.0047526826,1.9256869762,-0.5753909093
O,0,-4.7366575305,-1.9033418001,-1.4835005062
H,0,-5.0679133981,-2.1320812577,-2.3595365752
H,0,-5.491420975,-1.5195928103,-1.0042464832
N,0,-5.1192682109,0.9982615536,-2.1087431714
H,0,-5.8543346909,0.3289377522,-2.3250181069
C,0,-4.8081290745,1.7512908417,-3.3376639206
H,0,-4.2143396313,2.6226808843,-3.0362630491
C,0,-6.043746483,2.2104228524,-4.0883848227
H,0,-6.6732099499,2.8389573418,-3.4554881314
H,0,-5.7582170653,2.7964385997,-4.962386984
H,0,-6.632208525,1.3520302827,-4.4247043769
C,0,-3.8562878429,0.9191061828,-4.2040603209
O,0,-3.7307656763,1.1561099103,-5.4013750956
O,0,-3.1846147865,0.0026804152,-3.5886653447
C,0,-5.5462644341,1.8130793069,-0.9764536878
H,0,-6.35134485,2.5094208979,-1.2485754184
H,0,-4.7107096262,2.4233583037,-0.6231259751
C,0,-6.0856532246,0.9856369607,0.1580229492
O,0,-6.413923521,-0.1796395237,0.0288661551
C,0,-6.1983231812,1.694473966,1.4804897848
H,0,-5.1694924379,1.8977490503,1.8117731105
H,0,-6.6835254044,2.6627006033,1.3347636645
O,0,-6.9294958328,0.980996053,2.4441291146
H,0,-6.5305177991,0.1111127243,2.5466351452
```

## {B4} $[Fe(AMD)_2]^+$

```
Charge = 1 Multiplicity = 6
N,0,-1.3592267864,-1.9495358724,2.5300842497
H,0,-0.343463565,-1.9387825626,2.4605739108
C,0,-1.815355277,-3.3123708659,2.2124235544
C,0,-1.7445772855,-3.4893315313,0.6950785312
O,0,-1.753503903,-4.6097793251,0.1916539799
O,0,-1.7197720655,-2.3990199234,0.0052968612
C,0,-1.7275749526,-1.4342772156,3.8450682414
H,0,-2.6253891064,-1.934049189,4.223958567
H,0,-0.9481696064,-1.592878529,4.5941724244
C,0,-2.0515869452,0.0191446663,3.7535555302
```

O,0,-2.1448674897,0.5681830275,2.658968576
C,0,-2.2675367667,0.831597184,4.9863367688
H,0,-1.4696078855,1.586404416,4.9946929639
H,0,-3.2187129009,1.363523655,4.8646998025
O,0,-2.2482899571,0.0298404639,6.1420897258
H,0,-2.300173482,0.6113724622,6.9050341503
H,0,-2.8845333311,-3.3511674271,2.4509201373
C,0,-1.0813458929,-4.3880664209,2.9877426553
H,0,-1.4731519655,-5.3732200843,2.7328119975
H,0,-0.0116018194,-4.3696365082,2.7605933421
H,0,-1.2125702501,-4.2404213724,4.0617561502
Fe,0,-1.8975735739,-0.5806038391,0.842853362
N,0,-2.3825048763,0.1440847394,-1.2299736291
H,0,-2.1316354186,-0.7005534251,-1.7381379586
C,0,-1.4504720381,1.2082585035,-1.6356418913
C,0,-0.1819827918,1.1048348813,-0.7890103394
O,0,0.8355462576,1.7102995927,-1.1120976218
O,0,-0.2712136052,0.3936832822,0.2868052203
C,0,-3.7980438173,0.4046450962,-1.4606028436
H,0,-4.0013753949,1.4803846265,-1.4765154739
H,0,-4.1512131294,0.0122450878,-2.4178203983
C,0,-4.6157134277,-0.1750722638,-0.3544316239
O,0,-4.0746397131,-0.6556812854,0.6372560177
C,0,-6.1068417327,-0.1681876225,-0.4303744839
H,0,-6.4287607753,-1.2157008674,-0.3689180824
H,0,-6.4688733624,0.339604257,0.4720311471
O,0,-6.5612985359,0.4571538571,-1.6063265889
H,0,-7.5217825393,0.4301414062,-1.6013936961
H,0,-1.9025065242,2.1604096148,-1.3380464592
C,0,-1.1763368238,1.2165814094,-3.1277437547
H,0,-0.5151502637,2.0424218812,-3.3916256312
H,0,-0.7020235008,0.280635383,-3.4373015269
H,0,-2.1080887694,1.3353842272,-3.6845902835

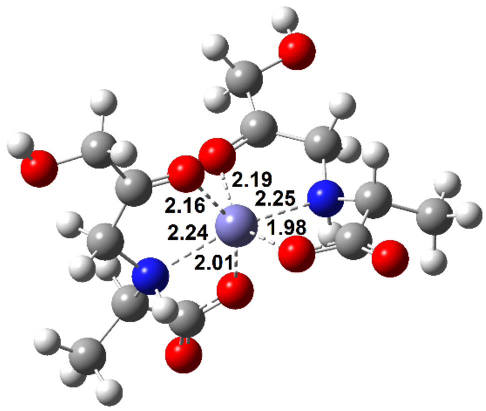


## {B5} [Fe(AMD)$_3$]

Charge = 0 Multiplicity = 6
N,0,0.4549685702,-1.2014797229,-1.1342793324
H,0,1.3150668227,-0.7377832322,-1.4272030353
C,0,-0.3929550193,-1.3433756771,-2.3301998289
H,0,-1.1206479742,-2.1354575568,-2.1238333234
C,0,0.4036381914,-1.6985039225,-3.5760838869
H,0,0.9760002858,-2.6156794304,-3.4202484054
H,0,-0.260746334,-1.8563474452,-4.4260940269
H,0,1.1028880514,-0.8947095393,-3.8270620694
C,0,-1.231584616,-0.0836095128,-2.5309454949
O,0,-1.8836705526,0.0601066233,-3.5661447062
O,0,-1.2349599103,0.7716334823,-1.5714169267
C,0,0.8542700326,-2.4684602018,-0.5288818968
H,0,0.8718377703,-3.2820516503,-1.268028742
H,0,0.1535310356,-2.7787716852,0.2476191414
C,0,2.2557046195,-2.4264924645,0.0168364907
O,0,3.0705594781,-1.6089290193,-0.3634483603
C,0,2.5899030788,-3.4758760494,1.0416429378
H,0,1.9794337924,-3.2606901635,1.9303113687
H,0,2.2722535663,-4.4527657469,0.6672456413
O,0,3.958576969,-3.5530789646,1.3503640408
H,0,4.2527262276,-2.6857454053,1.6464951244
Fe,0,-0.4007313019,0.5416169342,0.2322794253
N,0,-1.8373880923,-0.7394212432,1.4191133625
H,0,-2.4157156956,0.0003493842,1.8103511774
C,0,-1.1269333502,-1.407421337,2.5315313637
H,0,-0.9985008159,-2.4587740058,2.2527921862
C,0,-1.9062540227,-1.3353947697,3.8324195423
H,0,-2.9019059615,-1.7652439149,3.6996562963
H,0,-1.3966991146,-1.8970316389,4.6157334479

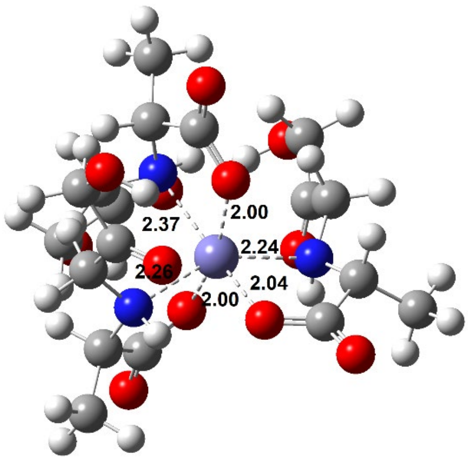

H,0,-2.0162526922,-0.2985944265,4.1628362226
C,0,0.3050703091,-0.8831261706,2.6629963082
O,0,0.9766129119,-1.1932000196,3.6472521801
O,0,0.7610106187,-0.1751828392,1.6908645338
C,0,-2.7099132272,-1.6413344686,0.6695151482
H,0,-3.2954107915,-2.2808912166,1.3463327076
H,0,-2.1172991528,-2.3123115715,0.0451951448
C,0,-3.7228774531,-0.9183707556,-0.1692471687
O,0,-4.0542366702,0.2330325666,0.0380984846
C,0,-4.338526487,-1.7042503117,-1.2915344798
H,0,-3.5361548348,-1.9196235845,-2.0113628829
H,0,-4.6910818949,-2.6675771804,-0.9082820833
O,0,-5.4007163623,-1.0165938239,-1.9041043303
H,0,-5.3377606983,-0.0932904034,-1.6252866529
N,0,0.9347295205,2.3383256543,0.0803641608
H,0,1.5154746874,2.2629696673,0.9152489839
C,0,0.1173836404,3.5616429618,0.1929858464
H,0,-0.2658073758,3.7853928562,-0.8086604973
C,0,0.9042430397,4.7484649347,0.7187832821
H,0,1.76889662,4.956171033,0.0848592733
H,0,0.2785364273,5.6413479433,0.7341775711
H,0,1.2567653898,4.555867736,1.7361988661
C,0,-1.1255371538,3.2641774447,1.0326827441
O,0,-1.8043585121,4.1916112947,1.4780592785
O,0,-1.4161185796,2.0256934704,1.2017494464
C,0,1.824566103,2.2940262441,-1.0791939788
H,0,2.1269600449,3.3003614627,-1.3983446901
H,0,1.3215458681,1.8344535745,-1.9331876695
C,0,3.1047260927,1.5673868682,-0.7599046502
O,0,3.4862492134,1.4233831312,0.3839995414
C,0,3.8720044766,1.0490109185,-1.9464463146
H,0,3.2038634063,0.3829775179,-2.508016948
H,0,4.0999986746,1.8938805702,-2.6038922827
O,0,5.0803539175,0.4175260784,-1.609528404
H,0,4.868501193,-0.3519992813,-1.0695802018

## {B6} $[Fe(ASC)(H_2O)_5]^{2+}$

Charge = 2 Multiplicity = 6

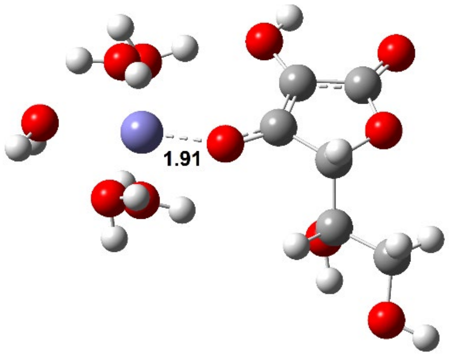


C,0,-2.2956906531,1.9415673265,-1.1141552705
O,0,-1.939866641,3.0415249242,-1.8117838832
C,0,-0.5371887575,3.2958780229,-1.6321653305
C,0,-0.0529760133,2.176440231,-0.7467251472
C,0,-1.1330507385,1.4045243958,-0.4456498995
O,0,-1.1879399154,0.3166716555,0.3489796805
H,0,-2.0690829423,-0.0794846588,0.3173633836
O,0,1.1867261789,2.0999361383,-0.4079707566
O,0,-3.4372413289,1.5256850173,-1.0949241619
H,0,-0.4421895782,4.2517028794,-1.1095656619
C,0,0.1804997921,3.3954997813,-2.9641788488
H,0,1.2224932293,3.6511377782,-2.7272715191
C,0,-0.3938771571,4.497532812,-3.8294453405
H,0,-1.4325394162,4.2720765904,-4.0895219504
H,0,-0.3657108617,5.4425699163,-3.2747071799
O,0,0.4005694438,4.5785096197,-5.0037982831
H,0,-0.0820628991,5.0871436886,-5.6598793257
O,0,0.131700566,2.1369831477,-3.6115582034
H,0,0.4908584576,2.2547266541,-4.4966411291
O,0,4.1661296923,2.2119442381,0.0707261949
H,0,3.8718896674,3.1270614446,0.1525875089
H,0,4.4554462409,2.1238558595,-0.8454157192
O,0,4.0358899287,-0.4992586245,1.3012323089
H,0,4.1578228569,-1.2295893993,0.6844797829
H,0,4.8774766322,-0.0299782011,1.2919015625
Fe,0,2.439282455,0.8993912659,0.3929859681
O,0,2.3680725623,1.7438131957,2.4613634041

H,0,3.1586131346,1.445800253,2.9264166602
H,0,2.4563963957,2.7027759862,2.4115557753
O,0,2.8396545385,-0.1848848472,-1.4676441123
H,0,2.7728826973,-1.1355666489,-1.3186157082
H,0,2.1545096089,0.0221621798,-2.114833058
O,0,1.2173576133,-0.7189885251,1.0695380135
H,0,0.2876578674,-0.470869042,0.9071706455
H,0,1.3068256733,-0.8085000349,2.0251945202

## {B7} $[Fe(ASC)_2(H_2O)_4]^+$

Charge = 1 Multiplicity = 6
C,0,-2.9280588451,0.5890497573,0.5670950384
O,0,-2.796104907,1.2932236624,-0.5861004437
C,0,-1.5092017022,1.9264963127,-0.6112530612
C,0,-0.8472815016,1.515907284,0.681738522
C,0,-1.7309995097,0.7260151126,1.3464807919
O,0,-1.5570459536,0.1578850094,2.5729346117
H,0,-2.2891584084,-0.4433754408,2.7607033173
O,0,0.3299423651,1.9285469696,0.999108569
O,0,-3.9450393694,-0.0332261497,0.82020308
H,0,-1.6718716265,3.0081855187,-0.6223467476
C,0,-0.7316688212,1.5449848793,-1.8577592407
H,0,0.2177035328,2.0952870549,-1.8092791874
C,0,-1.452090081,1.9394793592,-3.1291666201
H,0,-2.4175575231,1.4294131989,-3.1917640454
H,0,-1.6228060698,3.0216613599,-3.1256597651
O,0,-0.6268996633,1.5595419684,-4.2211958358
H,0,-1.1785977526,1.4692814104,-5.0018249936
O,0,-0.465758483,0.1473534728,-1.8375433726
H,0,-0.2132213629,-0.1062591244,-2.7334853773
Fe,0,2.0264857573,0.9743742394,1.3950021515
O,0,2.2287478107,2.2629246496,3.2063654408
H,0,3.1371022822,2.2199907285,3.5258389168
H,0,2.0957689697,3.1842712908,2.9559123991
O,0,1.8510078927,-0.2965886434,-0.3901222107
H,0,1.9320641813,-1.2205394687,-0.1312442053
H,0,0.9997199105,-0.2155642394,-0.869160862
C,0,7.0634170164,-0.0007811759,0.6828758561
O,0,6.9906321578,-1.2050693585,1.3043033129
C,0,5.6812223784,-1.3663343054,1.8679149261
C,0,4.9230050617,-0.1273986516,1.4608280759
C,0,5.7934314105,0.6624209296,0.7738611239
O,0,5.5685931703,1.9083804927,0.2748554805
H,0,6.2608361695,2.1434943222,-0.3559557083
O,0,3.6970649766,0.0206148757,1.8007176551
O,0,8.0915800313,0.3802585741,0.150551776
H,0,5.7917305426,-1.3874663987,2.9561250712
C,0,5.0342401828,-2.663387343,1.4240840941
H,0,4.0644191764,-2.7054488736,1.93940818
C,0,5.8428198649,-3.8727255785,1.8422284299
H,0,6.811735294,-3.8745159723,1.3335439912
H,0,6.0124134837,-3.8373735561,2.924662444
O,0,5.0987300874,-5.0298894764,1.4872959501
H,0,5.6891883274,-5.7866066286,1.5094247624
O,0,4.8303213945,-2.6247219874,0.0228014123
H,0,4.5335288694,-3.4985137212,-0.2498059454
O,0,1.0916858977,-0.6323094471,2.5376455153
H,0,1.4960903427,-0.6772454979,3.4110185665
H,0,0.1493697117,-0.4304043826,2.6917008607
O,0,2.9132829166,2.5732724486,0.2197664841
H,0,3.8828966439,2.4643369655,0.1994890941
H,0,2.7453183996,3.4186730938,0.6496542413

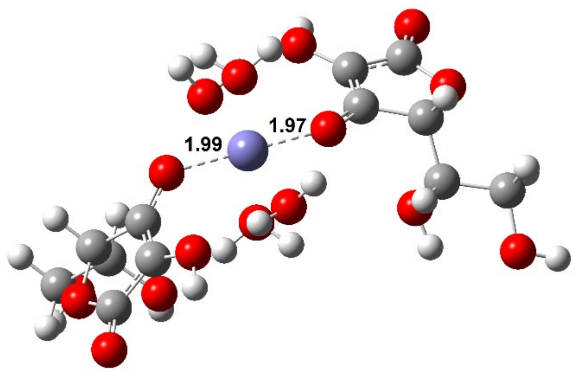

## {B8} [$Fe(ASC)_3(H_2O)_3$]

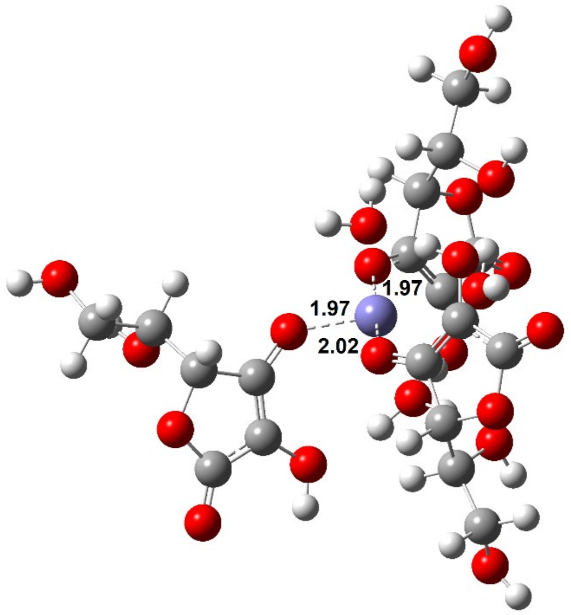


```
Charge = 0 Multiplicity = 6
C,0,-2.5996350988,3.0171983623,0.2450432833
O,0,-2.5261476715,4.0762144769,-0.6076980031
C,0,-1.150285258,4.3813664366,-0.86823515
C,0,-0.3728740245,3.3820216925,-0.0529429166
C,0,-1.2713829071,2.5819778029,0.5839205727
O,0,-1.0915868275,1.5403004906,1.4319671313
H,0,-0.1834056537,1.1883215807,1.3412577979
O,0,0.9168800465,3.3808829682,-0.0489045526
O,0,-3.6789330086,2.586036937,0.6116813823
H,0,-0.9613034356,5.3969632902,-0.5084729993
C,0,-0.8373521396,4.334446907,-2.3535545951
H,0,0.2106826761,4.6479724156,-2.4540569812
C,0,-1.6932834761,5.290164014,-3.1550497155
H,0,-2.7458403994,4.9978360442,-3.0964111946
H,0,-1.5830213576,6.3010774308,-2.7462947978
O,0,-1.2429219084,5.2395456347,-4.501912369
H,0,-1.92225073,5.6211679464,-5.0633345251
O,0,-0.9796580531,2.9988053306,-2.8201771926
H,0,-0.9185472376,3.0184299664,-3.7815361631
Fe,0,2.2923182813,1.998089871,-0.2898295061
C,0,3.5900648515,-1.6319239648,-3.4422348555
O,0,3.9257841067,-2.4524707554,-2.4056702551
C,0,4.1085837054,-1.6694854471,-1.2185916599
C,0,3.7590643555,-0.262276941,-1.6301491724
C,0,3.4921267112,-0.2829597969,-2.9657009212
O,0,3.1511697213,0.7271304181,-3.8103623041
H,0,3.1360826117,1.5715186073,-3.3193273904
O,0,3.7659460222,0.7111419497,-0.7901384264
O,0,3.4215422035,-2.0833880238,-4.5625877007
H,0,5.1690178149,-1.7245281343,-0.9520024962
C,0,3.3089629548,-2.2012794255,-0.0432395318
H,0,3.637476608,-1.6219158607,0.8308405337
C,0,3.584868892,-3.6642094823,0.2259016795
H,0,3.28440319,-4.2729407148,-0.6317759526
H,0,4.657883567,-3.8024940221,0.399074399
O,0,2.8340380358,-4.0344198798,1.373828347
H,0,2.7584389938,-4.9916026565,1.3892476563
O,0,1.9188299772,-1.9707849672,-0.2508883293
H,0,1.4404855466,-2.5936889776,0.3092402521
C,0,5.7110321754,1.5619257702,3.3429972521
O,0,6.5194967311,2.5126279976,2.8072549628
C,0,5.8155747123,3.1973181007,1.7613796285
C,0,4.455199828,2.5491159818,1.7179443893
C,0,4.4446502707,1.5774152082,2.6693766271
O,0,3.4095582918,0.7574249134,3.004297575
H,0,3.7203730198,0.0712315434,3.6080976793
O,0,3.5535994824,2.9498693657,0.8922046956
O,0,6.0914923482,0.8423452549,4.2511277374
H,0,6.3437809572,3.0005534879,0.8237045829
C,0,5.7906656598,4.6954898253,1.999218971
H,0,5.2489887379,5.1283263235,1.146826073
C,0,7.1838192576,5.2882025358,2.0247278236
H,0,7.7490650532,4.8889345009,2.8723790049
H,0,7.7054966186,5.0255721272,1.0971606001
O,0,7.0502045878,6.6981313472,2.1438075429
H,0,7.8910309317,7.0583737315,2.4357364016
O,0,5.0865094098,4.9588278562,3.1993519845
H,0,5.1735391862,5.8987282405,3.3854057675
O,0,0.7122692743,0.9866152272,-1.7557239891
H,0,1.1233759541,0.5242197594,-2.4921148603
H,0,0.1575325197,1.6737010499,-2.1710972897
O,0,1.4344198152,0.4803822343,1.0780959756
H,0,1.5065152634,-0.3767410168,0.6085578251
```

H,0,2.07825698,0.438844547,1.8105126374
O,0,2.9909284505,3.1059433505,-2.3139198348
H,0,3.8161640398,3.5710543272,-2.1361405992
H,0,2.3559469074,3.7952293055,-2.5383465618

## {B9} [Fe(PM)($H_2O$)$_4$]$^{2+}$

Charge = 2 Multiplicity = 6
C,0,-1.4892603739,-1.0233073097,-0.1514523257
C,0,-0.1108933489,-1.032760876,0.0296899822
C,0,0.5252067748,0.1900121127,0.2819466572
C,0,-0.2777875865,1.3388909543,0.3674730372
C,0,-1.6724510746,1.2406113521,0.1461378457
N,0,-2.2526634499,0.0720293511,-0.1122944022
H,0,-2.0055041223,-1.9594487118,-0.3479917545
C,0,-2.5133959398,2.4714596961,0.2064655995
H,0,-2.4320190255,2.9559903109,1.1840884375
H,0,-2.1845029915,3.2051825016,-0.5362486345
H,0,-3.5590171952,2.2286687761,0.0200132255
C,0,0.6243214123,-2.3383730644,-0.0178456782
H,0,-0.0419345653,-3.1145850762,-0.406627024
H,0,1.4876767482,-2.2742844053,-0.6894564678
O,0,1.0549399216,-2.6784056994,1.2999643906
H,0,1.5541088147,-3.4979400978,1.2435005362
C,0,2.0062148367,0.3047871467,0.499752762
H,0,2.2082175479,0.4635166533,1.5634674036
H,0,2.511457571,-0.6171485708,0.211807778
N,0,2.5963165107,1.4408051464,-0.2378919101
H,0,3.6068396529,1.4047578085,-0.1438501814
H,0,2.3948989965,1.3566932269,-1.2310591146
O,0,0.2313108371,2.521743435,0.6748077134
O,0,3.5811464175,4.1184009985,-0.8042268703
H,0,4.4290826267,3.6828436031,-0.6590850542
H,0,3.7443561454,5.0575661487,-0.6598132583
O,0,1.1246969698,5.4429002701,0.2752665494
H,0,0.2808821054,5.5253718941,0.7346551155
H,0,1.7183790283,6.0650241823,0.711669628
Fe,0,1.8592992793,3.396960376,0.4567995335
O,0,2.5808230341,3.6012770724,2.4311685499
H,0,3.401863056,4.1071960512,2.4609534746
H,0,1.9582845195,4.0786747369,2.9927597497
O,0,1.2019347317,3.4921544523,-2.5293111194
H,0,2.1003262162,3.6598420571,-2.2200913873
H,0,0.7562967792,4.334690917,-2.4056831766

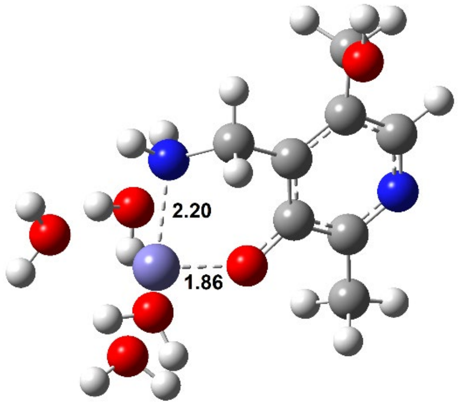


## {B10} [Fe(PM)$_2$($H_2O$)$_2$]$^+$ *trans*

Charge = 1 Multiplicity = 6
C,0,-0.8830003031,-0.8057698408,-0.9450683007
C,0,0.2671686002,-0.7869195178,-0.1656411018
C,0,0.6240768331,0.4215395709,0.4479542051
C,0,-0.1982387266,1.5437733189,0.2417058985
C,0,-1.336521528,1.4149441298,-0.5911981737
N,0,-1.6619945187,0.2569462532,-1.1637161829
H,0,-1.1839254183,-1.7319310746,-1.4287894872
C,0,-2.1987610866,2.608444842,-0.8434512557
H,0,-2.6079061492,3.0074310522,0.0900165759
H,0,-1.622972748,3.4187706691,-1.304113141
H,0,-3.024955314,2.348092681,-1.5050056066
C,0,1.0608121912,-2.0486355111,-0.0050691343
H,0,0.7270719343,-2.7822125952,-0.7458209719
H,0,2.1259849484,-1.8610095504,-0.1814173254
O,0,0.8715924412,-2.5670781669,1.3127101885

H,0,1.4262367985,-3.3473135393,1.4017929538
C,0,1.8245234348,0.5583896996,1.3399293584
H,0,1.4928393795,0.7736410441,2.3592607968
H,0,2.3941545748,-0.3698933882,1.3791394787
N,0,2.7022344279,1.6681742433,0.9224037942
H,0,3.4862058205,1.7285642585,1.5646454599
H,0,3.0951558742,1.4628600655,0.0078381085
O,0,0.0651769277,2.7092933237,0.8123313251
C,0,4.3706799828,8.2758415079,-0.3658798515
C,0,3.3129932114,8.1280247308,0.522255651
C,0,2.9895314087,6.8319717093,0.9471647031
C,0,3.7448415296,5.7553952311,0.4458856905
C,0,4.8022494082,6.0246799376,-0.458538827
N,0,5.1013331565,7.2642120136,-0.8429116415
H,0,4.6436398087,9.2706991961,-0.7097109747
C,0,5.6079045547,4.8909392002,-1.0027387895
H,0,6.0780115641,4.316948946,-0.1980894173
H,0,4.9748389971,4.1909731087,-1.5580999605
H,0,6.3863645726,5.2626938663,-1.6688535659
C,0,2.5621374929,9.3476103436,0.9686525797
H,0,2.4659574897,9.3731818926,2.0596038185
H,0,3.115485489,10.2414520903,0.6647173262
O,0,1.2661783423,9.348963818,0.3661154965
H,0,0.802525854,10.1340114479,0.6712858243
C,0,1.9038845977,6.5550795371,1.9493209172
H,0,2.3515485956,6.1167806677,2.8457145017
H,0,1.4077496952,7.4746844729,2.2557137488
N,0,0.9033530085,5.5933165959,1.4423558802
H,0,0.1965113789,5.4428398157,2.1557436297
H,0,0.4257325177,5.9991594312,0.6419150756
O,0,3.493076615,4.5036316684,0.8010127923
Fe,0,1.7531384966,3.6499263502,0.8806582606
O,0,1.8557392046,3.327011233,3.207497562
H,0,2.3801590615,4.0166798617,3.6277635887
H,0,0.9719123103,3.4286747489,3.5773128912
O,0,1.5595840267,3.9283777894,-1.4246158325
H,0,2.0487897814,4.7126634143,-1.6969321132
H,0,0.6385168144,4.1223218463,-1.6321926566

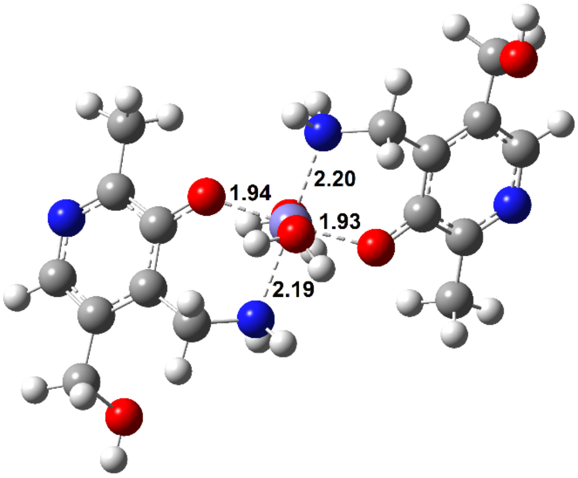


## {B11} $[Fe(PM)_2(H_2O)_2]^+$ *cis*

Charge = 1 Multiplicity = 6
C,0,4.4400246848,-1.639576807,-0.0043129677
C,0,3.8707009138,-0.8112220768,-0.9638239755
C,0,2.7932577354,-0.003144082,-0.5758312878
C,0,2.3535523131,-0.0717024282,0.7567758286
C,0,3.0034134696,-0.959211587,1.6497480824
N,0,4.0239824083,-1.7219772315,1.2628637797
H,0,5.2758782625,-2.2784611332,-0.2789643541
C,0,2.5351829413,-1.0420200541,3.0660369777
H,0,2.6220372457,-0.0730432335,3.5681196262
H,0,1.4783220301,-1.3243626515,3.1156158952
H,0,3.1204226188,-1.7757928036,3.6201977383
C,0,4.4430675443,-0.8003542552,-2.3489787759
H,0,5.1001501739,-1.6667006844,-2.4742834435
H,0,3.6482758016,-0.8738702339,-3.099708299
O,0,5.1848394917,0.405426089,-2.5449182234
H,0,5.5324842554,0.3926099332,-3.4411737198
C,0,2.1050620317,0.9559950261,-1.5032195145
H,0,2.2869975755,1.9813512209,-1.1665845537
H,0,2.4981447466,0.8753112498,-2.5168012161
N,0,0.6402589578,0.7561988794,-1.5191428615
H,0,0.2360669651,1.3898154374,-2.2024783915
H,0,0.4302410783,-0.1815960613,-1.850992608
O,0,1.3655553458,0.6843720883,1.1975839635
C,0,-5.1212669373,-1.810891658,0.39801842

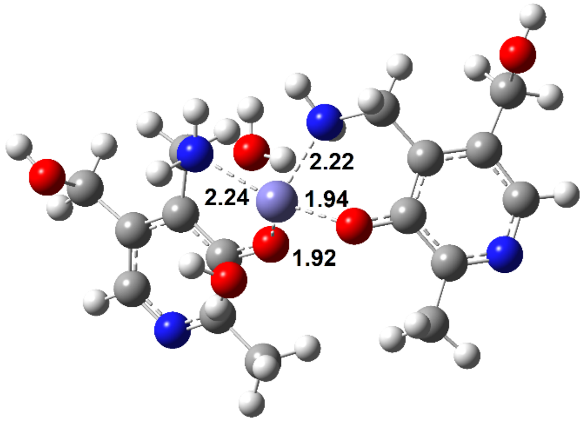

C,0,-4.7109078395,-0.88920605,-0.5566232515
C,0,-3.3773993444,-0.4588969246,-0.5203880524
C,0,-2.5288899851,-0.9936055397,0.464173592
C,0,-3.0590700836,-1.9190869217,1.3971808181
N,0,-4.3302064804,-2.3119956634,1.3512309369
H,0,-6.1498037079,-2.1636103216,0.391939243
C,0,-2.1731302489,-2.4741833913,2.4636732118
H,0,-1.3212090267,-3.0096625436,2.0330291097
H,0,-1.7567568025,-1.6735882875,3.0846662236
H,0,-2.7310421453,-3.15887723,3.1022987581
C,0,-5.7002369931,-0.3994459166,-1.5727204532
H,0,-5.2816668656,-0.4608219446,-2.583048063
H,0,-6.5906369,-1.035490688,-1.5451658425
O,0,-6.0579159975,0.9518893424,-1.2772199317
H,0,-6.6190203167,1.2677515315,-1.991003684
C,0,-2.8116743824,0.5264338843,-1.5015336807
H,0,-2.062381987,0.0288007661,-2.1249853539
H,0,-3.5847272586,0.9058692061,-2.1685852886
N,0,-2.141827614,1.6478293533,-0.8140068267
H,0,-1.847149967,2.3292845821,-1.5069230613
H,0,-2.8104823881,2.1277307826,-0.2168788787
O,0,-1.2467931174,-0.6723027189,0.5345779764
Fe,0,-0.3806588252,1.042749417,0.4262434359
O,0,-1.2880266457,1.6028294456,2.4968646895
H,0,-1.862292758,0.8699136522,2.7458721232
H,0,-1.8842031775,2.3503023305,2.3791387584
O,0,-0.0614381326,3.2751339712,0.2993911776
H,0,0.7851995878,3.4566963332,0.7233069572
H,0,0.0390241791,3.6084931707,-0.599560063

## {B12} [Fe(PM)3]

Charge = 0 Multiplicity = 6

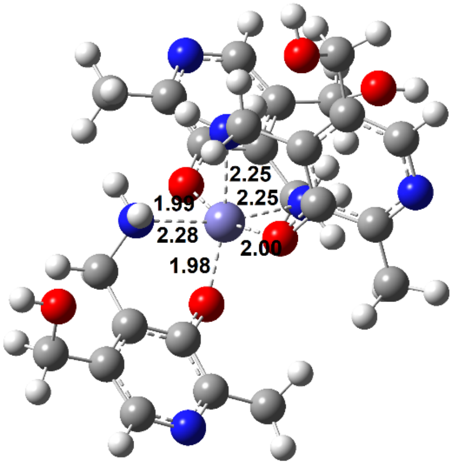


C,0,-4.5277073712,0.9419470062,-1.9608810416
C,0,-4.2247749464,-0.0823538163,-1.0726211539
C,0,-3.0768161742,0.0534902451,-0.2793775864
C,0,-2.2871048202,1.2113007968,-0.423468133
C,0,-2.6890232241,2.1850159399,-1.3764184093
N,0,-3.7866261273,2.0430151169,-2.1176728935
H,0,-5.4133779968,0.863204299,-2.5870150042
C,0,-1.8588330489,3.4146220117,-1.5571544971
H,0,-1.7854924106,3.9857386574,-0.6259337321
H,0,-0.833872834,3.1589910469,-1.847043834
H,0,-2.2906167096,4.0550684356,-2.3266211107
C,0,-5.1331239967,-1.2727047529,-0.9951546767
H,0,-5.8095396617,-1.2694652512,-1.8560291994
H,0,-4.5551065585,-2.2029692161,-1.0317187304
O,0,-5.8903321397,-1.2216695271,0.2162787754
H,0,-6.4089584837,-2.0293575699,0.2703223542
C,0,-2.6581983312,-0.9759276895,0.7295008142
H,0,-2.708355386,-0.5412265398,1.7330888181
H,0,-3.3322025359,-1.8332341765,0.7220619496
N,0,-1.268473892,-1.4129697465,0.5041869154
H,0,-1.04406106,-2.1590425816,1.1553993875
H,0,-1.1994726918,-1.8317284968,-0.4192882107
O,0,-1.2052524769,1.416361188,0.2987579252
C,0,2.2007333914,-4.1894887612,-2.1888883151
C,0,2.1203085754,-2.8745065324,-2.6301148396
C,0,1.9065048839,-1.8746384515,-1.6721240588
C,0,1.7707903074,-2.2482067043,-0.3222413563
C,0,1.8578091279,-3.6252424728,0.0103734554
N,0,2.0778940606,-4.5617009337,-0.910633641
H,0,2.3730287437,-4.9867031454,-2.9083513786
C,0,1.7021836431,-4.0416181099,1.4373967278
H,0,2.4524354349,-3.5626825652,2.074767899
H,0,0.7234651659,-3.7428845959,1.8295376339

H,0,1.8005597384,-5.1230899391,1.5333465678
C,0,2.2487334847,-2.5844824457,-4.0959408804
H,0,2.9804673132,-1.7896033154,-4.2760976648
H,0,2.6000151009,-3.4824731859,-4.6140315282
O,0,0.9740583271,-2.1926109465,-4.6128037974
H,0,1.0944166943,-1.9474452083,-5.5345771414
C,0,1.8330306801,-0.412475561,-2.0006302805
H,0,2.6424086923,0.1124151288,-1.4831164079
H,0,1.9623583184,-0.2364291103,-3.0687188018
N,0,0.5674131738,0.1768547677,-1.5267110804
H,0,0.5315030521,1.1541428053,-1.8017636137
H,0,-0.2149403111,-0.2711258538,-1.9953035615
O,0,1.5820121412,-1.3546918539,0.6254657818
Fe,0,0.3147818711,0.1756191201,0.7046875377
C,0,0.6684133986,4.0195693496,4.5347464975
C,0,0.667357264,2.6497703677,4.7701620584
C,0,0.9632862481,1.8035078257,3.6942946067
C,0,1.2221512035,2.3737071075,2.4347450073
C,0,1.1972987317,3.7855353437,2.3112698284
N,0,0.9320818083,4.5780511807,3.3495370665
H,0,0.4440926468,4.7032824866,5.350348989
C,0,1.4761022341,4.4115824596,0.9832705226
H,0,2.4610702996,4.1192331009,0.6056024308
H,0,0.7445474234,4.0868333897,0.2353815189
H,0,1.4400363272,5.4985695308,1.0587234645
C,0,0.3354810429,2.1389946197,6.1407451064
H,0,1.0928863622,1.4276205912,6.4880580552
H,0,0.3187835811,2.975897927,6.8460108866
O,0,-0.9456520105,1.5048081477,6.110063037
H,0,-1.1289426859,1.1733075038,6.9935752994
C,0,1.0341567084,0.3085345437,3.8025422099
H,0,2.0263236969,-0.0250251344,3.4837353051
H,0,0.8929079763,-0.0235568096,4.8316270814
N,0,0.0565741266,-0.3412857102,2.9097766948
H,0,0.1650012439,-1.3476222917,2.9861763741
H,0,-0.8838386993,-0.1275253125,3.2297988907
O,0,1.4930974493,1.6120460035,1.3903138262

### {C1} [Fe(AMD)($H_2O$)$_2$]$^{2+}$ (4-coord.)

Charge = 2 Multiplicity = 6
N,0,-1.9582805263,-1.8994394831,-1.2848911728
H,0,-2.5571882378,-2.6878980749,-1.5361004512
C,0,-1.5970013523,-2.0139016584,0.1425253285
H,0,-0.7392530847,-1.3527777134,0.3081066166
C,0,-1.2480348743,-3.4274558851,0.560870967
H,0,-0.4154581138,-3.8106845862,-0.0319187975
H,0,-0.9470321941,-3.439184845,1.6087418531
H,0,-2.1058441941,-4.0930450503,0.4353270832
C,0,-2.7198772408,-1.4007395649,0.9669715065
O,0,-2.8473446817,-1.626182062,2.1588842949
O,0,-3.4943132122,-0.586595241,0.3098222313
C,0,-0.8144390286,-1.8915231414,-2.2026113052
H,0,-0.0050697627,-2.5350082709,-1.8341207161
H,0,-0.3994009985,-0.8841820468,-2.2823426897
C,0,-1.1754880361,-2.4281598426,-3.5602972008
O,0,-2.1615514753,-3.1138006182,-3.740989033
C,0,-0.2403473955,-2.0464000017,-4.6734956078
H,0,-0.3972552831,-0.9695088626,-4.8464584195
H,0,0.794042603,-2.1658799019,-4.3424569019
O,0,-0.4144614849,-2.7976744635,-5.8466120191
H,0,-1.3424308876,-2.7598146309,-6.099309231
O,0,-3.2533728782,0.1288132219,-3.6595744968
H,0,-2.3747230202,0.2263756076,-4.0477759944

H,0,-3.7446737658,0.9211752836,-3.9125849634
O,0,-4.4562856312,1.6288838248,-1.2982495803
H,0,-4.2731011618,2.2960555107,-1.9707692566
H,0,-4.3518814071,2.0664360117,-0.4450084463
Fe,0,-3.2520946742,-0.1291765158,-1.5602435977

## {C2} $[Fe(AMD)(H_2O)_3]^{2+}$ (5-coord.)

Charge = 2 Multiplicity = 6
N,0,-1.9039002586,-1.8829313078,-1.2887197737
H,0,-2.565017249,-2.622099792,-1.5277350495
C,0,-1.5621271395,-2.0024531631,0.1417192074
H,0,-0.6503073731,-1.4162796583,0.3013175322
C,0,-1.3344081738,-3.4340464575,0.5844557512
H,0,-0.5254436339,-3.8929297604,0.012984311
H,0,-1.0546720852,-3.4572958653,1.6379880873
H,0,-2.2402723948,-4.0313188963,0.4508712395
C,0,-2.6290328152,-1.2820795,0.9610737294
O,0,-2.7702610553,-1.4936030502,2.1560540669
O,0,-3.3290579536,-0.4019219305,0.3096226298
C,0,-0.7613760842,-1.9844461923,-2.1980391117
H,0,-0.0540445316,-2.7571131902,-1.8671855041
H,0,-0.2104832349,-1.0429512694,-2.2177624601
C,0,-1.1755896402,-2.395011129,-3.5816597098
O,0,-2.208639408,-2.9971915103,-3.8011248585
C,0,-0.2478727324,-2.0093324285,-4.6955587886
H,0,-0.3309102991,-0.9140609741,-4.7893395145
H,0,0.7867122348,-2.2257588286,-4.4153961331
O,0,-0.5475593525,-2.6570286911,-5.9052459985
H,0,-1.4878374195,-2.8745246103,-5.8931703223
O,0,-3.1813968541,0.1701894498,-3.7035753328
H,0,-2.3159587185,0.277346591,-4.1177766106
H,0,-3.687540897,0.9521366912,-3.9584182852
O,0,-4.7264089194,1.3659494731,-1.4137904492
H,0,-4.615564431,2.1908084505,-1.901072374
H,0,-4.9218655336,1.6152594399,-0.5026589658
Fe,0,-3.0440958478,0.051044084,-1.5615132299
O,0,-1.2664505144,1.1640079866,-1.3449500006
H,0,-1.2054608206,1.8702014809,-2.0011671434
H,0,-1.2491228638,1.6024735582,-0.4841069389

## {C3} $[Fe(AMD)(H_2O)]^{2+}$ (4-coord.)

Charge = 2 Multiplicity = 6
N,0,0.9895568167,0.3581916118,0.9450163946
H,0,1.0931893918,0.5980869211,1.930409227
C,0,1.5074616411,1.4854444381,0.1486337572
C,0,0.418506307,2.5524903355,0.0862582595
O,0,0.6623327825,3.7044250145,-0.2282800077
O,0,-0.7897771569,2.1298961183,0.3476709477
C,0,1.609681872,-0.9451727683,0.7245466056
H,0,2.0826281995,-0.9954136682,-0.2613668754
H,0,2.390904337,-1.1666323572,1.4556708298
C,0,0.5680941887,-2.0088337729,0.7659243786
O,0,-0.623093378,-1.7047414234,0.8452340673
C,0,0.9343724152,-3.4496887792,0.7018234862
H,0,0.5475355172,-3.9101203008,1.6204294633
H,0,0.3748964106,-3.8814258308,-0.1376436277
O,0,2.322280933,-3.6203353316,0.5638961845
H,0,2.5046910205,-4.5641381092,0.5615672038
H,0,1.606498676,1.1283567831,-0.8821501778
C,0,2.8416090971,2.004184751,0.6438802645
H,0,3.1885242713,2.819227104,0.0080493515
H,0,2.7588635162,2.3726342287,1.670001653
H,0,3.5897822493,1.2096089141,0.6160108462
O,0,-3.1506241952,0.047349269,0.0450017872

```
H,0,-3.2853991205,-0.8058529051,-0.3863598164
H,0,-3.3721618497,0.7177896551,-0.6138635545
Fe,0,-1.2281439424,0.3124741024,0.8036383519
```

### {C4} $[Fe(AMD)(H_2O)_2]^{2+}$ (5-coord.)

```
Charge = 2 Multiplicity = 6
N,0,0.9766763226,0.3429367104,0.7246827112
H,0,0.7513361333,0.5204586053,1.7027254841
C,0,1.6813013961,1.5161536793,0.1876885264
C,0,0.6314145284,2.5702966121,-0.1606491386
O,0,0.9243050685,3.7470343272,-0.3041241892
O,0,-0.5747011745,2.1126744038,-0.3365652752
C,0,1.6560514292,-0.9419081744,0.6323953389
H,0,2.2439582794,-1.0024970861,-0.2899491697
H,0,2.345771802,-1.1225035895,1.460988188
C,0,0.6312815401,-2.023845259,0.5735465393
O,0,-0.557059324,-1.7303552312,0.4420545967
C,0,1.0216572922,-3.4590817513,0.6418406999
H,0,0.4676907349,-3.9025186069,1.4793717236
H,0,0.6485713896,-3.9265390945,-0.2781351978
O,0,2.4130238361,-3.6040700665,0.7813582776
H,0,2.6211517562,-4.5399750512,0.7145388145
H,0,2.1043443767,1.2178912353,-0.7777753909
C,0,2.7823878795,2.0229241148,1.0959337082
H,0,3.2779408874,2.8830678335,0.6446143056
H,0,2.3776778904,2.3232303087,2.0663695101
H,0,3.5311382829,1.2441075685,1.2542681155
O,0,-3.0021917244,0.4361781934,0.6122693905
H,0,-3.4827699608,-0.4008005186,0.6281911812
H,0,-3.54920305,1.0514777223,0.1085514856
Fe,0,-1.0811615032,0.2526866577,-0.173078733
O,0,-1.1563784345,-0.3053109697,-2.1989613052
H,0,-1.427829544,0.4384250636,-2.751818811
H,0,-1.80497811,-1.0027806368,-2.3581563862
```

### {C5} $[Fe(AMD)_2]^{+}$ (4-coord.)

```
Charge = 1 Multiplicity = 6
N,0,-1.7048459185,-1.648823232,-1.091138125
H,0,-2.3715614792,-2.2695725598,-0.6332476309
C,0,-0.7497445401,-1.1760791743,-0.0655334623
H,0,0.1508783503,-0.8483429894,-0.5954459699
C,0,-0.3875059664,-2.2494534835,0.943719063
H,0,0.0441221498,-3.1186581168,0.4445252611
H,0,0.3471433414,-1.8682535399,1.6532464101
H,0,-1.2728596486,-2.5685250635,1.5012276778
C,0,-1.2886510207,0.092281547,0.5943727265
O,0,-0.7952006607,0.5169758754,1.6319062865
O,0,-2.2501337061,0.6978635467,-0.0279485258
C,0,-1.0889874837,-2.3852234419,-2.192698363
H,0,-0.5210096707,-3.2508104418,-1.8356325339
H,0,-0.38122972,-1.7313789354,-2.7129640928
C,0,-2.1063034165,-2.8407786503,-3.2037378058
O,0,-3.2405034243,-2.4020981552,-3.2299767945
C,0,-1.6171509123,-3.8707455361,-4.1862418348
H,0,-0.6803091224,-3.5073765264,-4.6285443017
H,0,-1.3671881288,-4.7723679834,-3.6188335746
O,0,-2.5633313974,-4.2274106715,-5.1603350399
H,0,-2.7523107003,-3.456865083,-5.704364433
Fe,0,-3.0425784352,0.0273833174,-1.6934981052
N,0,-4.8942271144,1.1974850377,-2.1074592824
H,0,-5.4067834205,0.5179967882,-2.6696683614
C,0,-4.5508050448,2.3318650268,-2.9904328713
H,0,-4.287506458,3.1747358575,-2.342322933
C,0,-5.6836938868,2.7282115983,-3.9183534749
```

H,0,-6.5711642678,3.0081121065,-3.3483324718
H,0,-5.390655353,3.5845484946,-4.5262370972
H,0,-5.9417309881,1.9023883862,-4.5874613252
C,0,-3.2630638222,2.0042165132,-3.7473740658
O,0,-2.9201305461,2.6604621845,-4.7203614997
O,0,-2.5532579663,1.0246815731,-3.2711641945
C,0,-5.7273953572,1.5307453732,-0.9557798909
H,0,-6.6805611664,1.9780270765,-1.2569858178
H,0,-5.2119809734,2.2700003028,-0.3338370939
C,0,-5.9927203036,0.3269179445,-0.0892389136
O,0,-5.347019448,-0.7001459007,-0.1792876435
C,0,-7.1144210601,0.4853562198,0.9021760034
H,0,-6.9604691677,1.422023227,1.4535807952
H,0,-8.0387397539,0.605964948,0.3290409133
O,0,-7.2829477042,-0.6158381044,1.7566666644
H,0,-6.5039216873,-0.6951843555,2.3153897287

## {C6} $[Fe(AMD)_2(H_2O)]^+$ (5-coord.)

Charge = 1 Multiplicity = 6
N,0,-1.7910281987,-1.9805617703,-1.1688819393
H,0,-2.2898713418,-2.8281960141,-0.9034261297
C,0,-1.1500917947,-1.4174503896,0.0364907069
H,0,-0.2771908806,-0.8479230071,-0.2989798127
C,0,-0.7111371841,-2.4891025806,1.0160057727
H,0,-0.0311491235,-3.191578223,0.5295747843
H,0,-0.188770198,-2.0406320438,1.8612749106
H,0,-1.5742173025,-3.0430386493,1.3959016062
C,0,-2.0695959215,-0.3702068528,0.6635444188
O,0,-1.8499020484,0.0577214222,1.7910331572
O,0,-3.0410182382,0.0568323265,-0.0776248949
C,0,-0.8605286214,-2.3206202007,-2.2438016189
H,0,-0.1021059531,-3.0381317843,-1.9066429672
H,0,-0.3283422449,-1.4212271065,-2.5647568765
C,0,-1.5685795731,-2.9205341267,-3.4274503511
O,0,-2.7524430406,-3.1944569019,-3.4027332614
C,0,-0.7120877606,-3.1382375349,-4.6470035408
H,0,-0.4026759441,-2.1448515258,-5.0012796934
H,0,0.1978093384,-3.6677055829,-4.3537688665
O,0,-1.3390293317,-3.8860786474,-5.6576787932
H,0,-2.120278861,-3.4103409611,-5.956086033
Fe,0,-3.4416245422,-0.7025247379,-1.8434911575
O,0,-5.1116129456,-1.9797483822,-1.5849629448
H,0,-5.4656607692,-2.2849380488,-2.428386053
H,0,-5.8315822447,-1.4926041537,-1.1357322
N,0,-5.1145265962,0.9761477674,-2.0469761545
H,0,-5.8990751496,0.4279774099,-2.3918287799
C,0,-4.6965790665,1.8892606879,-3.1277074558
H,0,-4.1514472384,2.7153658692,-2.6580802442
C,0,-5.8644180334,2.4437509572,-3.9267621878
H,0,-6.5736064497,2.9533546139,-3.2716204513
H,0,-5.5131577128,3.1638660703,-4.6660250776
H,0,-6.3895321366,1.6388566306,-4.4491419823
C,0,-3.6590308556,1.2138854063,-4.0193760754
O,0,-3.3402662034,1.7114484796,-5.0931123058
O,0,-3.1211541078,0.1265507226,-3.5645080742
C,0,-5.5425257516,1.6807099436,-0.8433499515
H,0,-6.2720073676,2.4706721282,-1.0736117541
H,0,-4.6881571067,2.1804516158,-0.3821371755
C,0,-6.2167138601,0.8127771618,0.1760555192
O,0,-6.6141325471,-0.3165941454,-0.0544216918
C,0,-6.3906858765,1.432231113,1.5368437195
H,0,-5.3834734984,1.5029790236,1.9731632558
H,0,-6.764930967,2.4528876826,1.4313273215
O,0,-7.2771641131,0.7294702898,2.369519809
H,0,-6.9731496364,-0.1806189513,2.444741514

## {C7} $[Fe(ASC)(H_2O)_4]^{2+}$ (5-coord.)

```
Charge = 2 Multiplicity = 6
C,0,-2.1836717622,0.071935952,0.4139490032
O,0,-2.6203704191,1.1190659154,-0.3246297604
C,0,-1.5230616146,1.9944359829,-0.6400875469
C,0,-0.335613893,1.3471194971,0.0124406017
C,0,-0.7608967757,0.2078342543,0.6236554767
O,0,0.007886393,-0.6589026145,1.3038369115
H,0,-0.541921227,-1.359483043,1.6787513759
O,0,0.8544200024,1.8378999983,-0.0604671581
O,0,-2.9339224951,-0.8006689238,0.8035433698
H,0,-1.7362335307,2.9638542099,-0.182048389
C,0,-1.3749515426,2.1802127626,-2.1391543317
H,0,-0.5635061623,2.9093946277,-2.2713522611
C,0,-2.6311292918,2.7466506526,-2.7673012965
H,0,-3.4555835584,2.0339583434,-2.6703619169
H,0,-2.9053387485,3.675606257,-2.2538026805
O,0,-2.3530696325,2.9922539354,-4.1383483371
H,0,-3.1875325225,3.0645229696,-4.6076214824
O,0,-1.0057115022,0.942368528,-2.7202918201
H,0,-1.0072846641,1.057827467,-3.6756845883
O,0,4.0626710468,1.97230664,-0.5920713052
H,0,4.1619697328,2.861664844,-0.2307851522
H,0,3.8657604352,2.0921247377,-1.529227758
O,0,3.4383351402,-0.6491248471,1.5053056208
H,0,3.915783341,-1.2499354254,0.9209761641
H,0,4.0920953879,-0.3079484287,2.1272515181
Fe,0,2.490383276,0.9362696653,0.4119587522
O,0,2.7697722787,2.1330248832,2.199217832
H,0,3.6882724503,2.1123723504,2.4927424704
H,0,2.5790130996,3.0592468858,2.01013934
O,0,2.3110517161,-0.4329227014,-1.2966854266
H,0,2.4969177971,-1.3401734397,-1.0278226121
H,0,1.3988915352,-0.4416432061,-1.6119952332
```

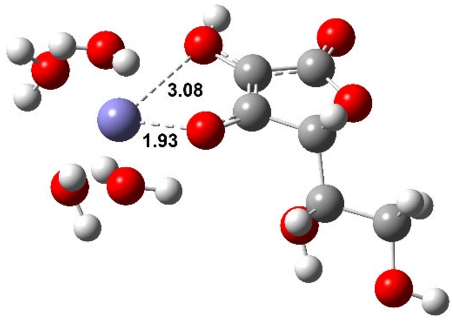


## {C8} $[Fe(ASC)(H_2O)_3]^{2+}$ (4-coord.)

```
Charge = 2 Multiplicity = 6
C,0,-2.0420028916,0.1179102298,0.7342730471
O,0,-2.4401676912,1.0871302233,-0.1213554428
C,0,-1.3239651056,1.9194872391,-0.4719229971
C,0,-0.1658683102,1.3281229327,0.2820360256
C,0,-0.6253743627,0.2660694181,0.9880040484
O,0,0.0830598962,-0.5391036477,1.8086000634
H,0,-0.4718764629,-1.264147242,2.1257516313
O,0,1.0379838409,1.8134655029,0.1909528139
O,0,-2.8110396831,-0.7112703635,1.1752609131
H,0,-1.5406465358,2.9291037819,-0.1120635053
C,0,-1.1192980379,1.9810708099,-1.9750833743
H,0,-0.3025971358,2.6952645919,-2.1423563549
C,0,-2.351568007,2.4785895616,-2.698477784
H,0,-3.189496246,1.7946451963,-2.5354254893
H,0,-2.6205565178,3.467467069,-2.3113550037
O,0,-2.0310718672,2.5412230954,-4.0801178922
H,0,-2.8501995858,2.5560629233,-4.5808632187
O,0,-0.7284695232,0.7010704411,-2.460537382
H,0,-0.892122163,0.6960265165,-3.4116279053
O,0,4.25349595,2.0944007807,-0.5834597242
H,0,4.2620784873,2.801470131,0.0747315004
H,0,4.2437567618,2.5394318535,-1.4405905003
O,0,2.7081374571,-0.7713151917,0.9366086494
H,0,3.3780170571,-0.656110974,1.6219263315
H,0,1.8447409441,-0.8168480143,1.395350206
Fe,0,2.5898211137,0.8451459499,-0.3503634801
```

O,0,1.9645563315,0.2260819179,-2.253658623
H,0,2.1266349586,-0.7131599086,-2.4005088155
H,0,0.9904003283,0.359221176,-2.3417967375

## {C9} $[Fe(ASC)_2(H_2O)_2]^+$ (4-coord.)

Charge = 1 Multiplicity = 6
C,0,-2.7556135462,0.7132363987,0.5434558807
O,0,-2.7428647698,1.4672240526,-0.5848900717
C,0,-1.4428190463,2.0507520945,-0.7564544362
C,0,-0.6522286366,1.5703063083,0.4265540299
C,0,-1.4606098508,0.7742502869,1.1718390221
O,0,-1.1304025738,0.1133020283,2.3062600018
H,0,-1.9048671383,-0.3579906227,2.6384931564
O,0,0.5947824812,1.8834196109,0.5761993579
O,0,-3.7508150068,0.1082988877,0.8964320861
H,0,-1.5620626968,3.1372207036,-0.7334021645
C,0,-0.8170510526,1.6585244775,-2.0834120375
H,0,0.1299738535,2.2106760929,-2.1447435015
C,0,-1.6773010519,2.0501472626,-3.2635399606
H,0,-2.624971567,1.5040783256,-3.2402785709
H,0,-1.8870304881,3.1247050461,-3.2128858255
O,0,-0.9543707171,1.7312661112,-4.4437081501
H,0,-1.5708458687,1.7133000732,-5.1795711336
O,0,-0.5468237925,0.2607111269,-2.0796689021
H,0,-0.3255566515,0.0078226483,-2.9831414155
Fe,0,1.9785121687,0.6738298954,1.2092797206
O,0,2.1357909156,1.9031833212,3.0336419986
H,0,3.0575753037,2.0540978529,3.2716045785
H,0,1.7585705311,2.7763351491,2.8785081365
O,0,1.6724043929,-0.5496853694,-0.5299868114
H,0,1.6584776809,-1.4993175513,-0.3712873987
H,0,0.8683250405,-0.3348587379,-1.0471885012
C,0,6.2550851434,1.6732722004,-0.6504673331
O,0,6.9769098802,0.6020628193,-0.2276195057
C,0,6.1669385454,-0.2215819318,0.6274590886
C,0,4.8439719534,0.4888299628,0.6855846623
C,0,4.9351945895,1.599319645,-0.0916171783
O,0,3.9433989758,2.4992982031,-0.3021526591
H,0,4.2616402016,3.1974020976,-0.887564162
O,0,3.8425053086,0.0515539133,1.3732846966
O,0,6.7388480557,2.5167213951,-1.3848355714
H,0,6.6384697925,-0.2356742452,1.6138473869
C,0,6.0819672686,-1.6448780869,0.1067880129
H,0,5.4896066175,-2.2007430507,0.8470611167
C,0,7.4446057619,-2.2971747674,0.0031118874
H,0,8.0555997902,-1.7797167183,-0.7424473497
H,0,7.9496747637,-2.2411050786,0.9742269296
O,0,7.2419781584,-3.6499898577,-0.381620565
H,0,8.0617426555,-3.9863287362,-0.7507174022
O,0,5.409532045,-1.6408037162,-1.1396199327
H,0,5.4719110196,-2.5298863208,-1.5025141599

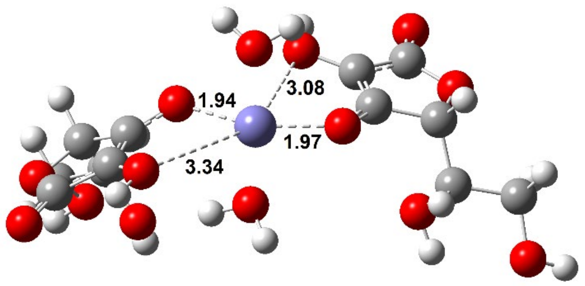


## {C10} $[Fe(PM)(H_2O)_3]^{2+}$ (5-coord.)

Charge = 2 Multiplicity = 6
C,0,-1.4934805087,-1.0220554913,-0.1242989361
C,0,-0.1108776201,-1.0064972268,0.0251118489
C,0,0.5063595012,0.2271258273,0.2670653137
C,0,-0.3137628665,1.3642966717,0.349688885
C,0,-1.7132247149,1.2385146219,0.1780300741
N,0,-2.2772366338,0.0573203299,-0.0584580096

H,0,-1.9959117401,-1.9675960525,-0.3117036683
C,0,-2.576639001,2.4518716401,0.2636866712
H,0,-2.4679363684,2.943109152,1.2353036819
H,0,-2.291930606,3.1880365854,-0.4943787431
H,0,-3.6231125238,2.1859025709,0.118845653
C,0,0.6477250028,-2.2973376432,-0.0521017152
H,0,-0.0059786409,-3.0762644964,-0.4566295959
H,0,1.5068162772,-2.2020951248,-0.7254125806
O,0,1.0899581413,-2.6602347879,1.2553746274
H,0,1.6031032201,-3.4692443512,1.1763673945
C,0,1.9832100572,0.3701683641,0.4890248543
H,0,2.1692114096,0.5756928424,1.5472685403
H,0,2.5042032199,-0.5555652087,0.2460326498
N,0,2.5687588181,1.482378635,-0.2870782466
H,0,3.5744232787,1.4824140824,-0.1441456876
H,0,2.4235822298,1.3201956937,-1.2809277416
O,0,0.1896730658,2.5626958757,0.6021660895
O,0,3.7089085492,4.0920627615,-0.6210225684
H,0,4.4632161427,3.7094006534,-0.1580813915
H,0,3.8126015626,5.0468800965,-0.53160202
O,0,1.1374713384,5.4655052572,0.4124158133
H,0,0.4575748766,5.5772781527,1.087236274
H,0,1.8473317029,6.0763043139,0.6426977273
Fe,0,1.7552162427,3.4650630433,0.1460410127
O,0,1.1476710333,3.2637653275,-2.5872253622
H,0,2.0906820454,3.4297155585,-2.6838200086
H,0,0.7408765087,4.115840326,-2.7708898354

**{C11} $[Fe(PM)(H_2O)_2]^{2+}$ (4-coord.)**

Charge = 2 Multiplicity = 6
C,0,-1.5000915279,-1.0548176228,-0.0844331729
C,0,-0.1153046244,-1.0340971271,0.0458168715
C,0,0.5041019975,0.2069385386,0.2381603925
C,0,-0.3169802038,1.3451321661,0.2959988378
C,0,-1.7185380143,1.2144189907,0.1441921592
N,0,-2.2839045864,0.0257903425,-0.0459351507
H,0,-2.0042599391,-2.0063990687,-0.2320954463
C,0,-2.5810791063,2.4300191456,0.194995631
H,0,-2.4448246668,2.9698175851,1.1366623636
H,0,-2.3200994922,3.1266368123,-0.6078600657
H,0,-3.6306839188,2.1565852345,0.0938175757
C,0,0.6424260911,-2.327005317,0.0041033283
H,0,-0.0162699029,-3.1192740027,-0.3646751198
H,0,1.4931744555,-2.2561328609,-0.6826958613
O,0,1.0996791837,-2.6432587324,1.3184204441
H,0,1.6005579539,-3.4620803191,1.2653629788
C,0,1.9838704552,0.3602706438,0.4309830753
H,0,2.1873157804,0.5945783048,1.4800266504
H,0,2.5039054116,-0.5701389503,0.2041748558
N,0,2.5528437732,1.4539238832,-0.3854183628
H,0,3.5612876636,1.4541635527,-0.262742006
H,0,2.3915521675,1.2595629863,-1.3708069955
O,0,0.1875823095,2.5505881386,0.505387117
O,0,3.7094248633,4.0768585325,-0.6705623124
H,0,4.4640324433,3.6576003754,-0.2413123934
H,0,3.8300830984,5.0239579484,-0.5335385822
O,0,1.1435317935,5.4489017944,0.3477799932
H,0,0.4611131321,5.5602454455,1.0200422909
H,0,1.8534597833,6.0580941751,0.582346605
Fe,0,1.7620186264,3.4470804049,0.0834702989

**{C12} $[Fe(PM)_2(H_2O)]^{+}$ (5-coord.)** non-planar

Charge = 1 Multiplicity = 6
C,0,-4.7233495065,1.0832836372,-1.2716048066

C,0,-4.2064027088,-0.2053471927,-1.2208232368
C,0,-3.1376271119,-0.4477635077,-0.3475211111
C,0,-2.6553196906,0.6183041401,0.4300958211
C,0,-3.2470703796,1.8967163209,0.2856835217
N,0,-4.2604254307,2.1091621334,-0.5517398997
H,0,-5.5530142511,1.2995275055,-1.9402504962
C,0,-2.724212522,3.041101837,1.0908641277
H,0,-2.8151245243,2.8446087902,2.1639145453
H,0,-1.6602462997,3.2076680135,0.892565957
H,0,-3.2712094225,3.9540731,0.8556850262
C,0,-4.8207765878,-1.2736955313,-2.0737227223
H,0,-5.4710857676,-0.8093431224,-2.8216212491
H,0,-4.04733937,-1.8363684388,-2.6086136917
O,0,-5.5813135334,-2.1585279101,-1.2487843345
H,0,-5.9515315435,-2.8414508779,-1.8152332086
C,0,-2.4984296306,-1.7957734685,-0.1831500331
H,0,-2.6815942165,-2.1664417391,0.8299398619
H,0,-2.9240931383,-2.5225607336,-0.8749111695
N,0,-1.0330171711,-1.7349990285,-0.3680746895
H,0,-0.6555206941,-2.6766708155,-0.3089647467
H,0,-0.8179161659,-1.400773845,-1.3036559633
O,0,-1.6764163746,0.445592578,1.3016154261
C,0,4.7616022801,1.151134098,-1.3422919006
C,0,4.3246052715,-0.1493236836,-1.127921598
C,0,3.0046895874,-0.3349512796,-0.6924292787
C,0,2.199973495,0.8013569155,-0.5007385946
C,0,2.7497313876,2.0826072123,-0.7556829604
N,0,4.0057072018,2.2388271405,-1.1673795585
H,0,5.7807852939,1.322604867,-1.6801116341
C,0,1.901946596,3.29707185,-0.5656911577
H,0,1.0081925777,3.2583813407,-1.1962954447
H,0,1.5504140801,3.3753360982,0.468106444
H,0,2.4668646802,4.1955571488,-0.8138402845
C,0,5.2737687551,-1.2861302321,-1.3694601627
H,0,4.8080808163,-2.0563049121,-1.9936156533
H,0,6.1526802415,-0.913938623,-1.9051701503
O,0,5.6695733224,-1.8494433833,-0.1177018415
H,0,6.2004942303,-2.6290785261,-0.3032111092
C,0,2.4137475951,-1.6978110433,-0.4724792984
H,0,1.655992114,-1.887764852,-1.239022305
H,0,3.1708082853,-2.474109565,-0.5718943128
N,0,1.7530456468,-1.8102877533,0.8427204222
H,0,1.435078677,-2.7674103272,0.9651815964
H,0,2.4366515294,-1.6499626774,1.57818562
O,0,0.9340499679,0.7165177792,-0.1166653639
Fe,0,0.0210029507,-0.4504813998,1.099770451
O,0,-0.2375543995,-1.703713538,2.929840112
H,0,-1.0695554242,-1.4407509828,3.3400464829
H,0,-0.3553627182,-2.6344635167,2.7071185522

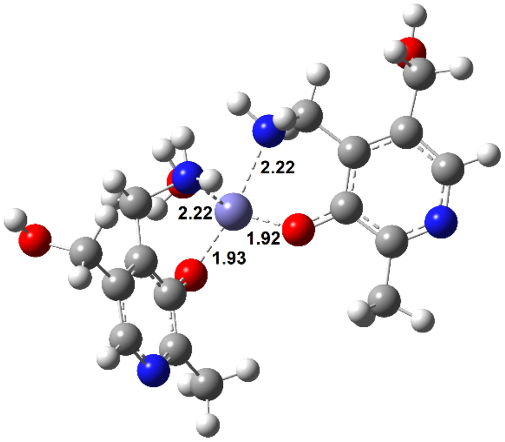


**{C13} $[Fe(PM)_2(H_2O)]^+$ (5-coord.)** planar

Charge = 1 Multiplicity = 6
C,0,-5.2446394405,1.0288090968,0.0910655895
C,0,-4.5726765803,-0.183474768,0.1817297042
C,0,-3.1761202549,-0.1695726102,0.05970776
C,0,-2.5354869138,1.0631328369,-0.1479232494
C,0,-3.3206936302,2.2394903727,-0.2231465768
N,0,-4.6469381035,2.2071279793,-0.1058915749
H,0,-6.3283048077,1.0469954558,0.177735988
C,0,-2.6469875081,3.5556303215,-0.4353666912
H,0,-1.9365992959,3.7682784353,0.3698637618
H,0,-2.0724738142,3.5590997754,-1.3671909637
H,0,-3.3811974374,4.3600006402,-0.4746256233
C,0,-5.3567002481,-1.4396058305,0.4177277263
H,0,-6.4177027543,-1.2450022477,0.2324545918

H,0,-5.0385423637,-2.2301584193,-0.2711417844
O,0,-5.1692622736,-1.8681096904,1.7679446279
H,0,-5.656505604,-2.6885799931,1.8851904469
C,0,-2.3376115071,-1.4099411178,0.1536850806
H,0,-1.6854469578,-1.3460280055,1.0298006143
H,0,-2.9586780525,-2.2963692588,0.2801836604
N,0,-1.4684022,-1.5637394613,-1.0292163119
H,0,-0.9473265557,-2.4305219578,-0.9427001527
H,0,-2.0419712918,-1.6510568176,-1.8631987274
O,0,-1.2172421395,1.1619463595,-0.2577382328
C,0,5.270291784,-1.0764109844,0.3676832748
C,0,4.5259012686,0.0642269868,0.6374353938
C,0,3.1555853515,0.0377616017,0.3427462869
C,0,2.6161549044,-1.1252098855,-0.2314073008
C,0,3.4656063546,-2.2394581276,-0.4446776892
N,0,4.7620569384,-2.200926794,-0.1449328598
H,0,6.335654441,-1.0838885322,0.5849467016
C,0,2.8958385999,-3.4973898116,-1.0132979326
H,0,2.0835201824,-3.8794131089,-0.3870979143
H,0,2.4703015161,-3.3254197824,-2.0071385582
H,0,3.6665961268,-4.2640014992,-1.0907449847
C,0,5.2121638731,1.2694304344,1.2094167132
H,0,4.6738434229,1.6473087218,2.0852047248
H,0,6.2195576116,0.9917575262,1.5349968835
O,0,5.286970395,2.2869946076,0.2087351808
H,0,5.664981096,3.0709899586,0.6167398705
C,0,2.2374051679,1.1884360622,0.6311234684
H,0,1.4840961323,0.8763603733,1.3612749233
H,0,2.7793774326,2.0287398855,1.0624791539
N,0,1.5169095548,1.6131919012,-0.5826809391
H,0,0.9626017212,2.4358648653,-0.3690807042
H,0,2.1858143645,1.8828190891,-1.2977418588
O,0,1.335030632,-1.2251224489,-0.5563979235
Fe,0,0.0449882861,0.0551552354,-1.2003180874
O,0,0.120119792,0.2277839931,-3.3751826706
H,0,1.0143770203,0.0484249045,-3.6887782338
H,0,-0.0731482352,1.1287727328,-3.6595675817

### {C14} $[Fe(PM)_2]^+$ tetrahedral (4-coord.)

Charge = 1 Multiplicity = 6
C,0,-4.7899599062,-0.0367809089,-1.6706334314
C,0,-4.4697544448,-0.6107822401,-0.4468456203
C,0,-3.2645109298,-0.2305901448,0.160873288
C,0,-2.4528822703,0.7046603881,-0.5046224472
C,0,-2.875784001,1.2232388322,-1.7513778984
N,0,-4.0246802872,0.8499302823,-2.311547066
H,0,-5.717299483,-0.3172640994,-2.1639845444
C,0,-2.0247350637,2.2264796117,-2.4574769367
H,0,-1.9139336711,3.1370560491,-1.8599971066
H,0,-1.01632231,1.8379911187,-2.6289016957
H,0,-2.4683034167,2.4913499021,-3.4169555631
C,0,-5.4240516502,-1.587654207,0.1722513583
H,0,-6.1597063284,-1.8961653507,-0.5767341956
H,0,-4.894822894,-2.4865561255,0.5080917077
O,0,-6.0847229431,-0.9715503632,1.2785945262
H,0,-6.6697426157,-1.6259460267,1.6711473115
C,0,-2.8501025254,-0.7487658177,1.508643936
H,0,-2.8732706212,0.0695953901,2.23371567
H,0,-3.541109672,-1.5117210095,1.8632845658
N,0,-1.4744239194,-1.2958497223,1.5134050849
H,0,-1.2846858917,-1.6872130961,2.4316047855
H,0,-1.4133391144,-2.071420281,0.8582384912
O,0,-1.3089638205,1.137816695,0.0197028438
C,0,4.9110649076,-0.0899665173,-1.3090555199
C,0,4.612416521,0.2692717586,-0.0005884328

C,0,3.3318852853,-0.0309955429,0.4828860037
C,0,2.4272114517,-0.6673223442,-0.3828540103
C,0,2.8361582166,-0.9885230982,-1.6983346791
N,0,4.060680896,-0.702706957,-2.1372758956
H,0,5.89668496,0.1324183125,-1.7104721481
C,0,1.876689493,-1.6614678485,-2.6234984861
H,0,1.5604797953,-2.6316760595,-2.2278614325
H,0,0.9686604509,-1.0634051193,-2.7492702381
H,0,2.3336887254,-1.8153153771,-3.6008099238
C,0,5.654536391,0.9646965257,0.8239365878
H,0,5.771645799,0.4756767889,1.79702407
H,0,6.6180373033,0.9096527002,0.3078000582
O,0,5.2766906005,2.3296690513,1.0088445885
H,0,5.9299138637,2.7388727554,1.5835634028
C,0,2.9018828192,0.262464418,1.8920612198
H,0,2.6592572327,-0.6771862794,2.3954428048
H,0,3.7013042912,0.7398228408,2.4558319578
N,0,1.6875570724,1.1107088663,1.9469583213
H,0,1.4736334326,1.3055225797,2.9210552084
H,0,1.8868827985,2.0102137368,1.516664046
O,0,1.1962740743,-0.9803439546,0.0149641535
Fe,0,-0.0008266013,0.1521988874,0.9729072802

## {D1} [Cu(ASC)($H_2O$)$_2$]$^+$ (3-coord.)

Charge = 1 Multiplicity = 2
C,0,-2.1435104345,0.2205532225,0.5788611878
O,0,-2.5529168837,1.258658906,-0.2047420195
C,0,-1.4069032012,2.0060745225,-0.6351525829
C,0,-0.232700598,1.2961198192,-0.0169495618
C,0,-0.7202638194,0.244729095,0.6933852237
O,0,0.0181688814,-0.6538259217,1.4102201945
H,0,-0.5781968663,-1.2702724181,1.8509028545
O,0,0.9743983213,1.7129641344,-0.2000095442
O,0,-2.950563376,-0.5533576261,1.0709311918
H,0,-1.4933839436,3.0145756158,-0.2206411688
C,0,-1.3442904891,2.1082481689,-2.1478757613
H,0,-0.4451428249,2.6985213606,-2.3730591199
C,0,-2.5423452589,2.835487138,-2.7208994503
H,0,-3.4582791394,2.2702309894,-2.5244978829
H,0,-2.627659122,3.8212880798,-2.249355544
O,0,-2.3347420478,2.9651621221,-4.120887922
H,0,-3.1837276711,3.1184497316,-4.5422309174
O,0,-1.2127201819,0.8078700856,-2.6934246405
H,0,-1.2760287001,0.8900861467,-3.6500543988
Cu,0,2.4403960986,0.6755249235,0.4511580254
O,0,3.8370009495,1.9310159125,-0.5488917137
H,0,4.627661358,2.0406309595,-0.0091813228
H,0,3.4596165351,2.8144204852,-0.6266147654
O,0,3.7869895639,-0.6364826103,1.1136026692
H,0,4.6434314202,-0.5007059613,0.6903734299
H,0,3.9519894502,-0.5328775811,2.0584583893

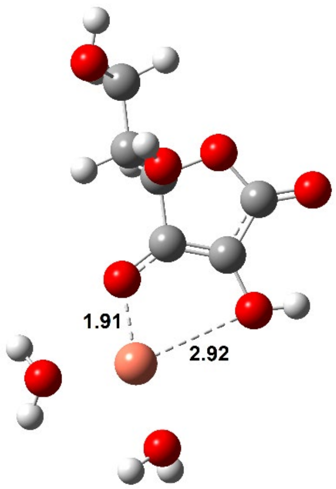


## {D2} [Cu(ASC)$_2$] (2-coord.)

Charge = 0 Multiplicity = 2
C,0,-5.5650251411,2.3558301865,-0.8277569655
O,0,-5.7227824637,3.4392915403,-1.64206865
C,0,-4.4318256657,3.9437650344,-2.0094031028
C,0,-3.4529638346,3.0143765895,-1.3452307064
C,0,-4.1723223681,2.0974625218,-0.6449342453
O,0,-3.6574274106,1.0827948858,0.1119896743
H,0,-4.374805362,0.6480320972,0.5875822422

O,0,-2.1823050479,3.1780034584,-1.4830600082
O,0,-6.5338788479,1.7706437598,-0.3674541998
H,0,-4.3326469373,4.9444841204,-1.5783730001
C,0,-4.2785205054,4.0529254769,-3.5142758655
H,0,-3.2667079699,4.4458860148,-3.6857283974
C,0,-5.2696003655,5.0272879472,-4.1147483315
H,0,-6.2915370281,4.6578273257,-3.983935214
H,0,-5.1753636685,5.9942801239,-3.607185458
O,0,-4.9651944615,5.1551463553,-5.4967126042
H,0,-5.7069885667,5.582984122,-5.9309486901
O,0,-4.3942499793,2.7615849351,-4.0845101227
H,0,-4.356850303,2.8615066821,-5.0405197319
C,0,3.7139422521,2.0370398827,-0.186470797
O,0,3.8949284927,0.9552606551,0.6256561433
C,0,2.6158019693,0.4167614958,0.9868481061
C,0,1.6167461637,1.310074149,0.3029828404
C,0,2.3161226587,2.2536919029,-0.3823903064
O,0,1.7772351217,3.2455868712,-1.1530103345
H,0,2.4630319966,3.8878802449,-1.3687495718
O,0,0.3494898685,1.1127980898,0.426820464
O,0,4.6694117644,2.6664362896,-0.6148673809
H,0,2.5116470262,0.5054606825,2.0721575425
C,0,2.5002366886,-1.0483790419,0.607623187
H,0,1.4971606316,-1.3645685436,0.9263481117
C,0,3.5186680397,-1.9060572882,1.3285737233
H,0,4.5327461702,-1.6199038755,1.0335740684
H,0,3.4115249209,-1.7622170441,2.409929192
O,0,3.2688428319,-3.2596711141,0.9754478956
H,0,4.0599893481,-3.7706996569,1.1615935684
O,0,2.6159201912,-1.1717669154,-0.7984887222
H,0,2.6256535676,-2.1119121772,-1.0029495344
Cu,0,-0.9202134967,2.1468703662,-0.5235652088

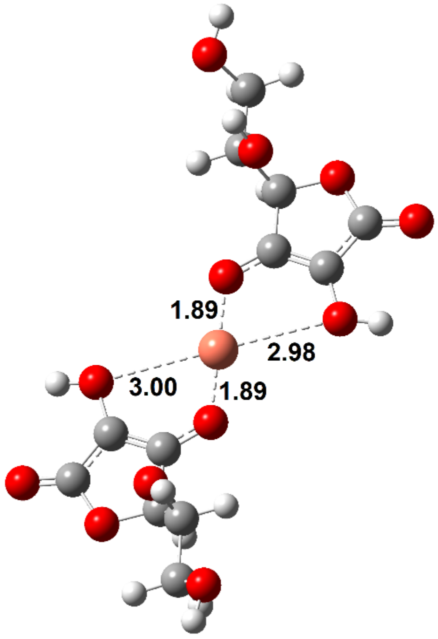


## {D3} $[Fe(ASC)(H_2O)_2]^{2+}$ (3-coord.)

Charge = 2 Multiplicity = 6
C,0,-2.2166623864,0.1114227872,0.4537051506
O,0,-2.6240188022,1.149210277,-0.3073966236
C,0,-1.4990592301,1.9577221885,-0.6989412381
C,0,-0.3183857861,1.2690427053,-0.0763637346
C,0,-0.7746572266,0.1786330765,0.6052290257
O,0,-0.0341119545,-0.6866795993,1.3011925654
H,0,-0.6013133475,-1.3606656685,1.7008589513
O,0,0.8853413061,1.7033353887,-0.2081739779
O,0,-2.9877567483,-0.7051824897,0.9103610025
H,0,-1.6385899611,2.9472553994,-0.2558791312
C,0,-1.418249139,2.1013407338,-2.2070060834
H,0,-0.5551783963,2.7543702031,-2.3984460113
C,0,-2.6530464628,2.7659116352,-2.7791414485
H,0,-3.5313460395,2.1346399946,-2.6151168583
H,0,-2.8106566565,3.7284532889,-2.2789210408
O,0,-2.4339861241,2.952630691,-4.1698613748
H,0,-3.284770849,3.0729999699,-4.5983866764
O,0,-1.1927701012,0.8253880025,-2.7758725244
H,0,-1.2259192139,0.9234361524,-3.7325025454
O,0,4.1008944977,2.010408569,-0.586815702
H,0,4.7448022224,2.3814325421,0.0290702811
H,0,3.7729035472,2.7581348435,-1.1018114059
O,0,3.4661793037,-0.6019227229,1.4063440996
H,0,4.0722748738,-1.0774668272,0.825317146
H,0,3.9992110344,-0.3067455635,2.1546383018
Fe,0,2.5283726395,0.9941394225,0.4023538528

### {D4} [Fe(ASC)$_2$]$^+$ (2-coord.)

```
Charge = 1 Multiplicity = 6
C,0,-2.6778320611,0.9495731485,0.6745344568
O,0,-2.6646755245,1.9045005565,-0.2890092794
C,0,-1.3405550413,2.0313557438,-0.8325842705
C,0,-0.5332451495,1.0132046446,-0.0809556932
C,0,-1.3538770948,0.3963045573,0.8048916532
O,0,-0.9922519416,-0.5731624266,1.6740351887
H,0,-1.7491734687,-0.8181703975,2.2208636011
O,0,0.731321732,0.8387408636,-0.3011230777
O,0,-3.6904772742,0.6732390123,1.2890559255
H,0,-0.9844347776,3.0390272493,-0.600273599
C,0,-1.3386096819,1.8502419304,-2.3389207099
H,0,-0.2922634841,1.967838057,-2.6539556045
C,0,-2.1643459361,2.9092350463,-3.0387747206
H,0,-3.2157227866,2.8263129128,-2.7464769762
H,0,-1.7953500781,3.901149505,-2.7532740204
O,0,-2.0244587714,2.7045487039,-4.4374119744
H,0,-2.7216901458,3.1899014796,-4.8845356303
O,0,-1.7894048439,0.544014143,-2.6454798309
H,0,-1.8625389193,0.4838345343,-3.6028495978
Fe,0,1.8819063829,-0.3854118052,0.5979702502
C,0,6.1555150215,1.4068956602,-0.8177569335
O,0,6.9643688975,0.6142564829,-0.0746179684
C,0,6.1702817119,-0.3105014171,0.6853659487
C,0,4.7529834191,0.0081439705,0.3001835877
C,0,4.781231366,1.034597615,-0.5865526366
O,0,3.7206728728,1.6306773967,-1.1691416192
H,0,4.0298647262,2.3221393387,-1.7683176201
O,0,3.7571082005,-0.6514345917,0.7994906589
O,0,6.5977327424,2.2801319867,-1.5399287901
H,0,6.3291225844,-0.0873292875,1.7442534258
C,0,6.5876229405,-1.7454392937,0.4238979682
H,0,5.9406227417,-2.3670943981,1.0584103154
C,0,8.0261252952,-1.9977567903,0.8239696111
H,0,8.7016829188,-1.3935483324,0.2105996523
H,0,8.1623428562,-1.7228409979,1.8762129628
O,0,8.2878651236,-3.3800259638,0.6275085855
H,0,9.2391725807,-3.5107489914,0.6244312011
O,0,6.3600287382,-2.0515347411,-0.9393044218
H,0,6.7199471285,-2.9292091046,-1.1014250188
```

### {D5} [Fe(ASC)$_2$($H_2O$)]$^+$ (3-coord.)

```
Charge = 1 Multiplicity = 6
C,0,-2.6116215679,0.5202249686,0.4580992488
O,0,-2.5705801144,1.4497843944,-0.5296647162
C,0,-1.2313190146,1.9499721576,-0.6665701102
C,0,-0.4422906005,1.1907535513,0.3616866902
C,0,-1.2937240527,0.3634024932,1.0163185857
O,0,-0.9801174072,-0.4845606624,2.0234448889
H,0,-1.7789450385,-0.9396847496,2.3177333321
O,0,0.8300196475,1.3765164682,0.5052145739
O,0,-3.6464727623,-0.0429977057,0.7634731359
H,0,-1.250108728,3.0141431527,-0.4143096357
C,0,-0.7196134828,1.7976831554,-2.0867517618
H,0,0.3004972832,2.2076394735,-2.0830611259
C,0,-1.5464448013,2.5929398414,-3.0751592835
H,0,-2.5702181223,2.2075778757,-3.1092243002
H,0,-1.5740217373,3.6421606909,-2.7592902323
O,0,-0.9301237385,2.4679566127,-4.3488763131
H,0,-1.5623085676,2.7379753666,-5.0191986875
O,0,-0.6820563815,0.4213186655,-2.4163822752
H,0,-0.43381477,0.3529779199,-3.343343039
```

Fe,0,2.0439218425,0.5625190508,1.7266215586
O,0,2.0431423867,1.7824126133,3.4168464519
H,0,2.9363296781,2.0160497336,3.6978199538
H,0,1.5835985338,2.6182018579,3.2701912431
C,0,5.7822028565,1.3391299825,-1.4151541283
O,0,6.6749491363,0.5170625043,-0.8134110856
C,0,6.0866226642,-0.044968655,0.369151453
C,0,4.6991693063,0.5343692089,0.4157884815
C,0,4.5539459933,1.3493698451,-0.6598790854
O,0,3.469577239,2.0822192244,-0.9950831134
H,0,3.6599867126,2.5868145702,-1.7960467778
O,0,3.8948612133,0.2391044258,1.3855028661
O,0,6.0552645992,1.947875721,-2.4330764018
H,0,6.6674588406,0.3083241824,1.2258924582
C,0,6.1337756598,-1.5617511998,0.343466803
H,0,5.6740789682,-1.8929235952,1.2849545896
C,0,7.5556309749,-2.0812293907,0.3031477353
H,0,8.0391416289,-1.7876707356,-0.633679611
H,0,8.1184800136,-1.6530432248,1.1406299954
O,0,7.5009609135,-3.4968797861,0.4070601231
H,0,8.3474428238,-3.8533299531,0.1273887479
O,0,5.3682829379,-2.0258147902,-0.7535870469
H,0,5.4629890335,-2.9823532594,-0.7919241854

## {D6} [Fe(ASC)$_3$] (3-coord.)

Charge = 0 Multiplicity = 6
C,0,-1.6941566523,1.2181153546,-0.4298902165
O,0,-2.0409413756,2.3532756195,-1.0990599442
C,0,-0.9438048659,3.2750975401,-1.0828541667
C,0,0.1384018268,2.5611568046,-0.322547988
C,0,-0.33742616,1.3411823376,0.0422759972
O,0,0.284806559,0.4133467074,0.8126321491
H,0,0.4087627673,-0.4187564621,0.3059513743
O,0,1.2823268786,3.1191743742,-0.0916151149
O,0,-2.4865053198,0.3018797957,-0.3059861589
H,0,-1.2616766363,4.167414134,-0.5350484162
C,0,-0.5540669542,3.6921853975,-2.4901172542
H,0,0.2973582423,4.3776051242,-2.3866860243
C,0,-1.6763326833,4.4211229623,-3.1973655924
H,0,-2.5522262823,3.7722231477,-3.2899085559
H,0,-1.952197336,5.3078960137,-2.6161701473
O,0,-1.1983154751,4.7863357154,-4.4840964554
H,0,-1.9537805345,4.9599735961,-5.050661515
O,0,-0.1507731353,2.5459640643,-3.2299461147
H,0,-0.1852338023,2.7829775821,-4.1644716299
Fe,0,2.9860502102,2.232743125,0.1366468015
C,0,2.1691471533,-0.6916333866,-3.4853792398
O,0,2.4099814523,-1.8948054364,-2.8884028484
C,0,2.9100497557,-1.6843842961,-1.5613898773
C,0,2.9456363856,-0.1904954683,-1.4078300408
C,0,2.4881097906,0.3637674396,-2.5611906484
O,0,2.4388597366,1.6910389693,-2.8548334815
H,0,1.5095900056,1.9746383409,-2.9952016227
O,0,3.3683378155,0.3810379343,-0.3274925025
O,0,1.749957393,-0.6380541089,-4.627988523
H,0,3.9243459915,-2.0920388393,-1.5151486482
C,0,2.057183811,-2.4026164005,-0.529532554
H,0,2.4904025605,-2.1654684127,0.4511991642
C,0,2.0794383626,-3.9049892305,-0.7169597789
H,0,1.6921863207,-4.1705896108,-1.7050023699
H,0,3.1110771539,-4.2632551596,-0.630917763
O,0,1.2598859128,-4.4683507554,0.2973835117
H,0,0.9886530573,-5.3457198762,0.0172150812

O,0,0.7246010134,-1.9091447567,-0.5880549648
H,0,0.1620887518,-2.5630293878,-0.1556449728
C,0,6.5052796996,2.243930971,3.4419774439
O,0,7.1660445966,3.3642315901,3.0482525846
C,0,6.4653028464,3.9716885895,1.9526626113
C,0,5.2706502798,3.0865298306,1.7316670997
C,0,5.3355053225,2.0696754825,2.6259824105
O,0,4.4534921364,1.0462553564,2.754032719
H,0,4.7050869892,0.5030909957,3.5106828546
O,0,4.3906004291,3.3602548955,0.8220685821
O,0,6.916829083,1.5511677434,4.3560395991
H,0,7.1238751367,3.9361036255,1.0797911731
C,0,6.1250800856,5.4219226857,2.2420052171
H,0,5.6032618752,5.7911810489,1.3481924232
C,0,7.3643279913,6.2668022216,2.4484153798
H,0,7.9026662891,5.9365868022,3.3421020256
H,0,8.0235817219,6.1615726724,1.5792138756
O,0,6.942858947,7.6154246566,2.5979021967
H,0,7.6615509074,8.1151407507,2.9922376863
O,0,5.2586093173,5.478968129,3.3605940958
H,0,5.1317586506,6.4071184605,3.5803250734

## {E1} $[Cu(H_2O)_2]^+ \cdot 2H_2O$

Charge = 1 Multiplicity = 1
Cu,0,-0.0132023766,0.0390416648,0.1187538989
O,0,1.6237839754,1.1690451124,0.0684467062
H,0,2.4331140325,0.6197893607,0.145866631
H,0,1.6602343574,1.791023754,0.8009138957
O,0,-1.6507503775,-1.0917297672,0.1586077716
H,0,-1.722276289,-1.6405293264,-0.6275897492
H,0,-2.4553347856,-0.5299878067,0.1630250234
O,0,-3.8242884985,0.573493956,0.2984795494
H,0,-4.6588723871,0.1190147586,0.4472946494
O,0,3.810180471,-0.4798001348,0.1544823027
H,0,4.6037869629,-0.104042406,-0.2377611046
H,0,4.0799224595,-0.737508436,1.040930654
H,0,-3.9662759944,1.0765675106,-0.5089523486

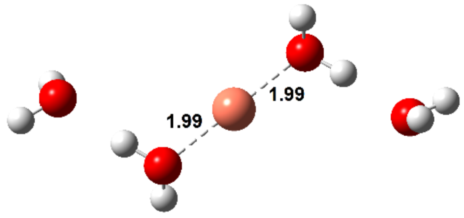


## {E2} $[Cu(PM)(H_2O)] \cdot H_2O$

Charge = 0 Multiplicity = 1
C,0,-1.5497004193,-0.2292725453,-0.2213849943
C,0,-0.2123080803,-0.5579555937,-0.0462039381
C,0,0.6389687552,0.3879817757,0.5456283163
C,0,0.1102283798,1.6471553706,0.9459197675
C,0,-1.2905610145,1.8553023925,0.710086227
N,0,-2.0781266573,0.94502147,0.1449056303
H,0,-2.227628536,-0.9460458172,-0.6799220688
C,0,-1.9045764538,3.1565967474,1.1203548428
H,0,-1.7325111166,3.3594076274,2.1828758542
H,0,-1.4667354242,3.997305754,0.5717271075
H,0,-2.9793684424,3.1455180451,0.935293828
C,0,0.2495560974,-1.9219923516,-0.4717576364
H,0,-0.4521624357,-2.3295329613,-1.2069600767
H,0,1.2354516221,-1.8809377173,-0.9462129208
O,0,0.3009031074,-2.7850347097,0.6698508893
H,0,0.6250070345,-3.6403695255,0.3740508075
C,0,2.0949874218,0.101526134,0.775955653
H,0,2.4384505207,0.6507579187,1.6521563278
H,0,2.253489217,-0.9611865127,0.9707051728
N,0,2.9548857689,0.5198419492,-0.3683407966
H,0,3.9040635575,0.2257367593,-0.1554762622

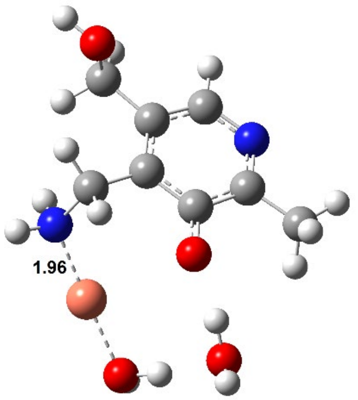

```
H,0,2.6877678351,-0.0327228669,-1.1778603209
O,0,0.8351249207,2.5648954208,1.5039186244
Cu,0,2.9823761244,2.424617085,-0.8369612106
O,0,1.152004968,5.1246620298,0.6981443048
H,0,1.5408290072,5.5802158414,1.449007779
H,0,0.9625601611,4.2103560175,1.0224756459
O,0,2.9992530794,4.3855660447,-1.1672788822
H,0,2.67756132,4.6091169253,-2.0456330804
H,0,2.3404086918,4.7604733029,-0.5384845901
```

## {E3} $[Cu(PM)_2]^-$

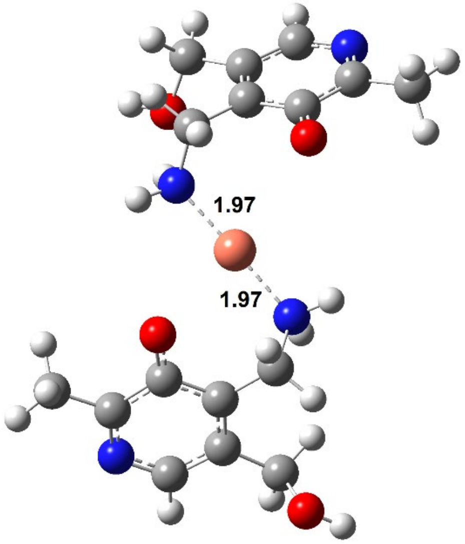


```
Charge = -1 Multiplicity = 1
C,0,-1.3705125616,-0.2893883863,-1.0100316366
C,0,-0.0466807214,-0.2239343345,-0.5947190233
C,0,0.280667859,0.6193185736,0.4777676994
C,0,-0.7464686047,1.3791126942,1.1110644487
C,0,-2.0736609052,1.2137906108,0.5805795661
N,0,-2.3595818221,0.412831469,-0.4409114595
H,0,-1.6469828384,-0.9334392939,-1.8422338757
C,0,-3.1905213692,1.9912405255,1.2026675565
H,0,-3.2893689717,1.7646612684,2.2699494971
H,0,-3.0114694304,3.0701624812,1.1349496092
H,0,-4.137090031,1.765757851,0.7098146523
C,0,0.9659824188,-1.0825767712,-1.296839713
H,0,0.581024229,-1.3662012968,-2.2818762785
H,0,1.9079908849,-0.5459869973,-1.4536146969
O,0,1.2090809425,-2.262385321,-0.5216193024
H,0,1.8636264709,-2.7882457879,-0.9895061458
C,0,1.6901594527,0.7480119179,0.9765675213
H,0,1.6820772906,0.9612653833,2.0454771142
H,0,2.2401553435,-0.1842931655,0.8327771295
N,0,2.4307153075,1.8584861126,0.3114540847
H,0,3.3893302001,1.8284108419,0.6475691838
H,0,2.4935964881,1.6386094778,-0.6782683593
O,0,-0.5251002993,2.1749864658,2.1018380397
Cu,0,1.7526472853,3.6913365147,0.5584439098
C,0,4.6699709814,7.8795923098,-0.4504005338
C,0,3.3793182578,7.6743772284,0.0194910082
C,0,3.1774890339,6.7371532771,1.0456024574
C,0,4.2927322678,6.017275884,1.5663028787
C,0,5.5749199961,6.3420203842,0.9987176781
N,0,5.7426190366,7.2365894145,0.0304226031
H,0,4.8507636621,8.599442981,-1.2456988593
C,0,6.7837563961,5.6318582258,1.5204190161
H,0,6.9109743608,5.7985635625,2.5958143484
H,0,6.698829534,4.5476940817,1.387020025
H,0,7.6833170239,5.9747113234,1.0078715093
C,0,2.2585900272,8.4606401442,-0.5945647032
H,0,1.6534102629,8.9492565014,0.1774021959
H,0,2.6737706123,9.2432325514,-1.237398072
O,0,1.4234501201,7.5928176332,-1.3731662357
H,0,0.6947893703,8.1181387667,-1.7158398141
C,0,1.8151489575,6.4740647484,1.6150623908
H,0,1.915004728,6.0574566651,2.6163970717
H,0,1.2495226878,7.4069642729,1.7001783486
N,0,1.0299128931,5.5040899066,0.8034677627
H,0,0.1145802646,5.4129299565,1.2352084738
H,0,0.8636794238,5.9461897138,-0.0984020503
O,0,4.1883664839,5.1275486343,2.4949039791
```

## {E4} $[Fe(H_2O)_6]^{2+}$

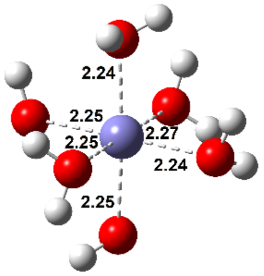


```
Charge = 2 Multiplicity = 5
Fe,0,0.5480206471,-0.5020279532,0.0855502379
O,0,0.3470246467,1.7255976515,0.0327313423
H,0,0.9289275093,2.1507977846,-0.6052707506
O,0,-1.6993790635,-0.6609487218,0.1206184435
H,0,-2.0173083273,-1.1437621679,-0.6495485265
O,0,2.779923116,-0.4407520038,-0.0773033764
H,0,3.0363704432,-0.8733213297,-0.8987709475
O,0,0.6353152455,-0.3947256572,2.3288518721
H,0,0.5508113424,-1.2686381341,2.7236287017
O,0,0.6494501538,-2.7422039122,0.2151951916
H,0,0.1392016886,-3.1721793388,-0.4784918149
O,0,0.463562215,-0.6595754576,-2.1730760917
H,0,0.4925555661,0.1997561645,-2.6055694359
H,0,0.569146741,2.1290921701,0.8779428366
H,0,1.2426326207,-1.1218813624,-2.4985356582
H,0,3.0992751985,0.4631715396,-0.1679978709
H,0,0.2678192548,-3.0633992114,1.0385487477
H,0,-0.0877769747,0.1170199871,2.7048077024
H,0,-2.0940630233,0.2136789527,0.0400683968
```

## {E5} $[Fe(PM)(H_2O)_4]^{+}$

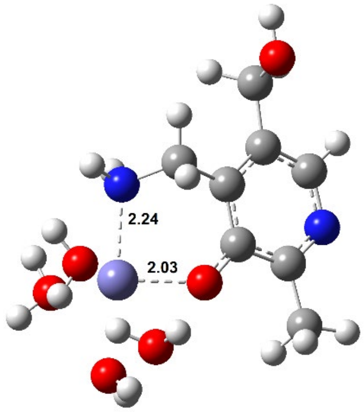


```
Charge = 1 Multiplicity = 5
C,0,-1.2663783192,-0.9367754799,-0.6246512139
C,0,0.0249286431,-0.9315454509,-0.1117411156
C,0,0.5369332691,0.2763118682,0.387362648
C,0,-0.2646995575,1.4363110612,0.3019699346
C,0,-1.5954071664,1.2909128882,-0.1820504844
N,0,-2.0659803629,0.1327206688,-0.6412157308
H,0,-1.6802694699,-1.8573318528,-1.0300701117
C,0,-2.5188453078,2.4679378995,-0.1560956996
H,0,-2.5309845915,2.9377049774,0.8319945774
H,0,-2.2111629321,3.2428926432,-0.8661712184
H,0,-3.5336701088,2.1605021155,-0.4091486742
C,0,0.7942098102,-2.2191175613,-0.0946137605
H,0,0.3458522776,-2.9162440914,-0.8098291045
H,0,1.8347170304,-2.0590061203,-0.3969653011
O,0,0.7515188739,-2.7838889389,1.2188013263
H,0,1.2550496401,-3.6026746792,1.2016588131
C,0,1.877354363,0.3598087236,1.0642987957
H,0,1.7287020783,0.7297876987,2.0840738136
H,0,2.3265765647,-0.6300567786,1.1528055335
N,0,2.806736943,1.2951935835,0.3997460925
H,0,3.6981313838,1.2434996168,0.8830451395
H,0,2.9836029366,0.970224473,-0.5455185404
O,0,0.1616155513,2.623207469,0.6784364838
O,0,3.051592905,4.037012207,-1.4311108567
H,0,3.685449088,3.4094769317,-1.7929176394
H,0,3.5188414899,4.8775635892,-1.3852286796
O,0,1.3740013275,5.5335441539,0.6896867522
H,0,0.7072220229,5.6736377769,0.0084912064
H,0,0.9304304397,5.7410026317,1.5179686058
Fe,0,2.0392135029,3.3909604293,0.5527770367
O,0,3.2780407817,3.6502318429,2.3452382668
H,0,4.1334152488,3.2085085722,2.3583331208
H,0,3.4587541349,4.5660473802,2.5797350904
O,0,0.204158775,4.0974504987,-2.0011532118
H,0,1.1713093855,4.1191954534,-1.9855059837
H,0,-0.0115066507,3.6867897997,-1.1529729108
```

## {E6} $[Fe(PM)_2(H_2O)_4]$

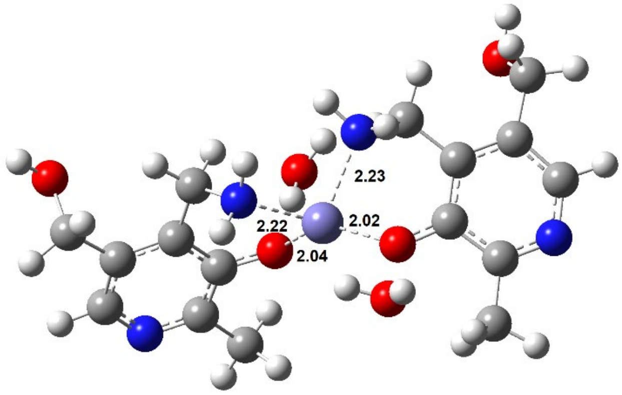


```
Charge =  0 Multiplicity = 5
C,0,-5.3843448554,0.4287175381,0.3202866416
C,0,-4.693235354,-0.556002357,-0.3740877178
C,0,-3.2951474226,-0.5907698815,-0.2586989888
C,0,-2.6604728606,0.388940731,0.5336580087
C,0,-3.4743901063,1.3335771757,1.2132667553
N,0,-4.8016630039,1.3445293188,1.0986218664
H,0,-6.4681449962,0.4783142402,0.2440372451
C,0,-2.8278108412,2.3571883229,2.0906535407
H,0,-2.1663040643,1.8897026637,2.8267955332
H,0,-2.2081844373,3.044692142,1.5051182867
H,0,-3.5834910056,2.939215161,2.6184008768
C,0,-5.4730838619,-1.5416075133,-1.1927661726
H,0,-6.4772386526,-1.1446659838,-1.3725328896
H,0,-5.0003714266,-1.7023943343,-2.1678791201
O,0,-5.5643498651,-2.7822628793,-0.4871910095
H,0,-6.052753865,-3.3978738082,-1.0409643443
C,0,-2.4629009242,-1.6527489146,-0.9227463657
H,0,-1.8925927568,-2.187802205,-0.1562629044
H,0,-3.0969997265,-2.3905043458,-1.4145022971
N,0,-1.4860281483,-1.0888276867,-1.8796507967
H,0,-1.0381228411,-1.8636440943,-2.3581000859
H,0,-1.9909268708,-0.5768867547,-2.5961563549
O,0,-1.3489396936,0.4323292691,0.6792817086
C,0,5.4453287774,0.8219085872,-0.3395775022
C,0,4.7508475633,-0.379782314,-0.3940067757
C,0,3.4275861657,-0.3585061687,-0.8583188835
C,0,2.8542074838,0.8799451978,-1.2208650125
C,0,3.6712553336,2.0421035128,-1.1371545804
N,0,4.9321009784,1.9990780015,-0.7107357665
H,0,6.4730045949,0.8324862792,0.0162708022
C,0,3.1020762324,3.3640612968,-1.5429646593
H,0,2.7379368317,3.340087319,-2.5751259986
H,0,2.2451461457,3.6392080889,-0.9184669392
H,0,3.856201233,4.1468226382,-1.4577651082
C,0,5.4372939126,-1.6355511438,0.0553261907
H,0,5.3648966128,-2.4181326656,-0.7080068964
H,0,6.4999869327,-1.4287559499,0.2173012406
O,0,4.8409078854,-2.0928205518,1.2723380313
H,0,5.266131325,-2.9201791761,1.5145412425
C,0,2.6039160178,-1.6046275501,-1.0167055337
H,0,2.2481583571,-1.6626956755,-2.0503395244
H,0,3.2050027399,-2.4957212295,-0.8334937023
N,0,1.4066583129,-1.5952145299,-0.1510143641
H,0,0.9254833126,-2.4816008999,-0.265858475
H,0,1.7020008774,-1.5628656828,0.8203479727
O,0,1.6113167524,0.9794067582,-1.6424158807
Fe,0,0.0424213867,0.0638950145,-0.763678635
O,0,0.6798319665,2.3116767121,1.3737788501
H,0,-0.126161127,1.7777372576,1.2705522107
H,0,1.1264659188,2.1965095381,0.529466627
O,0,-0.5173923593,-0.9264349929,2.9774063269
H,0,-0.7743180967,-0.4474677875,2.1663375648
H,0,0.2533985122,-1.4383916882,2.7206537625
```

## {E7} $[Fe(PM)_3]^-$

```
Charge = -1 Multiplicity = 5
C,0,-4.3480455906,0.7719920653,-2.2315958512
C,0,-4.0910289696,-0.3011133103,-1.3856819166
C,0,-3.1061057601,-0.1476492526,-0.3998311247
C,0,-2.4189266771,1.0858756101,-0.3071984062
C,0,-2.7644181082,2.106427392,-1.2401632933
```

```
N,0,-3.7052221457,1.9426075649,-2.169136746
H,0,-5.107268441,0.6774905526,-3.0051378264
C,0,-2.0347980087,3.4113627906,-1.1862882794
H,0,-2.1172340107,3.8769092454,-0.1988090279
H,0,-0.9637885937,3.2698162305,-1.3729535243
H,0,-2.4281192501,4.1031137526,-1.932008473
C,0,-4.8888310024,-1.5597700493,-1.5538792014
H,0,-5.3715081713,-1.5558409463,-2.5365247968
H,0,-4.2418228763,-2.4424468016,-1.5045382566
O,0,-5.8851714357,-1.6341872196,-0.5289027539
H,0,-6.3636837015,-2.4597167865,-0.6453440446
C,0,-2.7457028609,-1.2348130629,0.5749457373
H,0,-2.8631887583,-0.8487222936,1.5933196068
H,0,-3.4261349153,-2.0840117411,0.4794199348
N,0,-1.3399715964,-1.6504265621,0.4279519867
H,0,-1.1459033233,-2.392356938,1.0929847295
H,0,-1.2083201523,-2.0603966068,-0.4910964873
O,0,-1.4960032107,1.2983435107,0.5934826329
C,0,1.754097737,-4.160704637,-2.1473129378
C,0,1.8172467353,-2.8278579284,-2.5386703958
C,0,1.8377553224,-1.8453488698,-1.5395860917
C,0,1.7744980066,-2.2437900155,-0.1833082102
C,0,1.7090828369,-3.6411012527,0.0900511862
N,0,1.7059579689,-4.5625850372,-0.8723831395
H,0,1.7391886642,-4.9435130076,-2.9026701655
C,0,1.6251194745,-4.0954458751,1.51274752
H,0,2.5006845466,-3.7746662251,2.0872179302
H,0,0.7514028343,-3.6636510124,2.0150081103
H,0,1.5550984352,-5.1824920345,1.5655237681
C,0,1.842321539,-2.5016243498,-4.0023327708
H,0,2.6737871617,-1.8290095057,-4.2398520187
H,0,1.9858617726,-3.4217441364,-4.5780349606
O,0,0.60558974,-1.8861746427,-4.3768835836
H,0,0.6714175171,-1.640895873,-5.3040532234
C,0,1.9087883893,-0.3718921682,-1.8278692755
H,0,2.7256696574,0.0624914385,-1.2431482164
H,0,2.1322460811,-0.1866334821,-2.8810022116
N,0,0.6724778311,0.3137681222,-1.4109376374
H,0,0.7692160362,1.3064730469,-1.5986413385
H,0,-0.0956513579,-0.0062292804,-1.9925216578
O,0,1.7583812634,-1.3706809281,0.7882259439
Fe,0,0.1891644508,0.0869472894,0.8434114213
C,0,0.8722573819,4.1588525545,4.5250735075
C,0,0.8653211661,2.787630277,4.7510428358
C,0,1.1166424882,1.9332830904,3.6678072534
C,0,1.3421715438,2.4917922828,2.3833180507
C,0,1.3345819749,3.9189855093,2.2875356415
N,0,1.1092872072,4.7142817097,3.3308056757
H,0,0.6820878728,4.8433489726,5.34902326
C,0,1.5925167934,4.5516656801,0.9568317021
H,0,2.5578527962,4.2363792395,0.5464458538
H,0,0.8339042406,4.2557309429,0.2232483879
H,0,1.588911896,5.6389984089,1.0417009357
C,0,0.5849539434,2.2845776919,6.1367003932
H,0,1.3620677565,1.5857552701,6.4656985036
H,0,0.5815841141,3.1273859691,6.8355755922
O,0,-0.68822368,1.6314569149,6.1627477371
H,0,-0.8138960712,1.27304306,7.0456666895
C,0,1.1666309002,0.4391768951,3.8095294113
H,0,2.0901572655,0.0688522888,3.3535169434
H,0,1.1872873852,0.1467591202,4.8615264403
N,0,0.0489248099,-0.2105774863,3.0984400171
H,0,0.0920441693,-1.2103291963,3.2688566932
H,0,-0.8278214966,0.1060832819,3.5002079523
O,0,1.5604134591,1.7596117435,1.3299388594
```

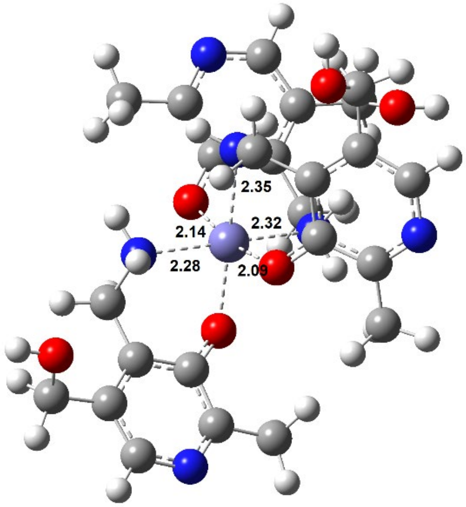

## {F1} $[Cu(H_2O)_4]^{2+}$

```
Charge = 2 Multiplicity = 2
Cu,0,0.0113164963,-0.0062243347,0.0097231411
O,0,1.9140133948,-0.7145563283,0.3020305878
H,0,1.9807349509,-1.636489633,0.0252958416
H,0,2.5433892571,-0.2369565749,-0.2518414035
O,0,-0.7979071443,-1.8702290581,0.1589600187
H,0,-1.5974079542,-1.9260361675,-0.3784848832
H,0,-0.2069465222,-2.5451772268,-0.1950481928
O,0,0.7353863288,1.8926155175,-0.3316553127
H,0,0.1919874746,2.5408451741,0.1320904315
H,0,1.618310247,1.9864520972,0.0456218549
O,0,-1.9435517268,0.6325979124,-0.1147636595
H,0,-2.4500078173,0.291060506,0.6315668071
H,0,-1.9978459848,1.5928211163,-0.039158231
```

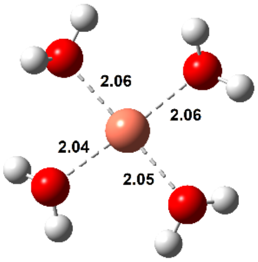


## {F2} $[Fe(H_2O)_6]^{3+}$

```
Charge = 3 Multiplicity = 6
Fe,0,0.5112394738,-0.4336466633,-0.0261871528
O,0,0.5817499339,1.7043221437,0.0661055836
H,0,1.3648090614,2.0368426561,-0.3908720969
O,0,-1.6523268471,-0.3514392415,0.1371132801
H,0,-2.0160528412,-1.240148818,0.0376499341
O,0,2.6679911333,-0.5217215281,-0.1967853412
H,0,3.0349572659,-1.0160951372,0.5467803002
O,0,0.6545197795,-0.5445881548,2.1265779498
H,0,0.1089473262,-1.2689640468,2.4584906012
O,0,0.4397544879,-2.5820025749,-0.1032840185
H,0,0.8543789144,-2.8965253035,-0.9169029103
O,0,0.3486543548,-0.3633628922,-2.1747142003
H,0,-0.5781171275,-0.2387372685,-2.4161515805
H,0,0.6621384966,2.0123225949,0.9778401591
H,0,0.602004061,-1.2121809018,-2.5595397991
H,0,3.0331460984,0.3686484058,-0.1201326225
H,0,0.9645156382,-2.9573666144,0.6156193404
H,0,0.2890083411,0.2556791034,2.5238177079
H,0,-2.0306775506,0.1652962409,-0.5854271343
```

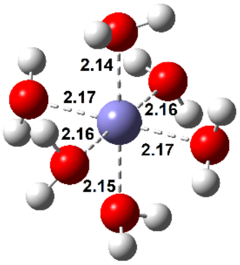


## {F3} AMD $^-$

```
Charge = -1 Multiplicity = 1
N,0,0.5365353737,0.3233878815,0.3988786384
H,0,0.1049248072,0.4832933081,1.3021838848
C,0,1.3372509377,1.5004181577,0.0615651573
C,0,0.4432853262,2.7480054031,0.0291360255
O,0,0.7015877879,3.6144386004,-0.8477782781
O,0,-0.4543049726,2.8478507646,0.9043490144
C,0,1.2748810805,-0.9131477444,0.4350151873
H,0,1.6542013531,-1.1604541386,-0.562250438
H,0,2.168263529,-0.9065413952,1.0834416709
C,0,0.4671273037,-2.0712417974,0.9265209401
O,0,-0.6438949408,-1.9482769595,1.4149506239
C,0,1.0702518844,-3.4507230672,0.8516875033
H,0,1.0841960498,-3.8468539793,1.8741758494
H,0,0.3736255635,-4.0663290233,0.2692210735
O,0,2.3614500948,-3.4524409268,0.2805490221
H,0,2.684683325,-4.3570869393,0.2865194234
H,0,1.7298668022,1.3507939165,-0.9477926399
C,0,2.512669023,1.78479089,1.0004127327
H,0,3.0208853343,2.7068702451,0.7053818339
H,0,2.1648348523,1.8997550714,2.0320104758
H,0,3.2546934851,0.9835087329,0.9763352994
```

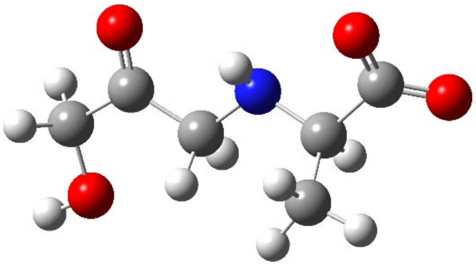

## {F4} $[(AMD)_2]^{2-}$

```
Charge = -2 Multiplicity = 1
N,0,-2.2836726282,-2.2030816173,-1.2666774205
H,0,-2.3811966354,-3.2049196508,-1.149386413
C,0,-2.1482254419,-1.5902416755,0.0509226518
H,0,-2.0739240081,-0.5092013596,-0.0945152352
C,0,-0.937561848,-2.0578512493,0.8621132963
H,0,0.0008217696,-1.8156715796,0.357402588
H,0,-0.9178964673,-1.5688603263,1.8399973477
H,0,-0.9729871239,-3.1404345079,1.0198337657
C,0,-3.41549436,-1.8527915175,0.8807080433
O,0,-3.801574257,-0.933680701,1.6452528679
O,0,-3.9609144358,-2.9859470843,0.779402509
C,0,-1.220896917,-1.9415912415,-2.2071079911
H,0,-0.2006531952,-2.0692904502,-1.8007174279
H,0,-1.2713451737,-0.9099806357,-2.5689193012
C,0,-1.2632252089,-2.8961927067,-3.3698484991
O,0,-1.6809692168,-4.0340215914,-3.2529916188
C,0,-0.7560660549,-2.3628538018,-4.6846911481
H,0,-1.4816945098,-1.5990593406,-5.0033607036
H,0,0.1873371416,-1.836311352,-4.5099219144
O,0,-0.5489674427,-3.3365168453,-5.6719810013
H,0,-1.4107871769,-3.7266456833,-5.9298685542
N,0,-5.4169340767,-5.5337528689,-5.5423965281
H,0,-6.2079080927,-6.1445907937,-5.3724615956
C,0,-4.2261557392,-6.3874886892,-5.6295584672
C,0,-2.9981563639,-5.6933023977,-6.2483658149
O,0,-2.0435928819,-6.4506845373,-6.5585791877
O,0,-2.9925802477,-4.4417916488,-6.4028964283
C,0,-5.41253592,-4.5194115473,-4.5071521285
H,0,-6.1139698522,-3.716731653,-4.77613053
H,0,-4.4361243803,-4.0403505231,-4.4501500894
C,0,-5.8609800356,-4.9385912404,-3.1240290203
O,0,-6.6537819664,-5.8442394556,-2.9354246716
C,0,-5.2675717954,-4.1207847379,-2.0198400155
H,0,-4.1975214151,-4.3701591621,-2.019586737
H,0,-5.3404539808,-3.06360185,-2.3087168687
O,0,-5.8518172911,-4.3624399239,-0.7654957648
H,0,-5.3006114465,-3.8832976864,-0.1211943309
C,0,-3.8185211677,-7.0867160039,-4.3290772403
H,0,-3.4707426263,-6.3754022888,-3.5729387499
H,0,-4.6615908619,-7.6454581795,-3.9155907861
H,0,-3.0058885876,-7.7910764661,-4.515257443
H,0,-4.4933173009,-7.1703194387,-6.3454109834
```

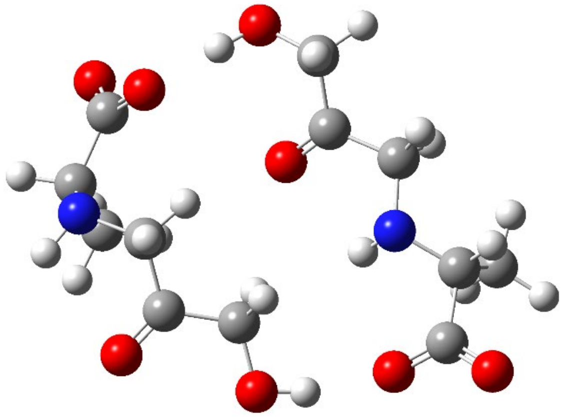

## {F5} $[(AMD)_3]^{3-}$

```
Charge = -3 Multiplicity = 1
N,0,-1.5991419187,-2.9416245953,-0.9969258229
H,0,-2.5274374237,-3.3400672709,-1.0859454167
C,0,-1.2087450356,-3.0142724631,0.4116769608
H,0,-0.2393298545,-2.5188087471,0.5062063919
C,0,-1.0829569812,-4.4296138707,0.9783089947
H,0,-0.2880469386,-4.9894670309,0.4799012444
H,0,-0.8412830627,-4.3946963148,2.0444824149
H,0,-2.0204007958,-4.9816264226,0.8572020154
C,0,-2.2153058891,-2.2365082534,1.2702619709
O,0,-1.7483230234,-1.532832001,2.2114975046
O,0,-3.434804452,-2.3843027384,1.0236096478
C,0,-0.6986204749,-3.6397679078,-1.9012918243
H,0,-0.7178394499,-4.7357483917,-1.8044354358
H,0,0.3242947443,-3.3114970936,-1.7030205779
C,0,-1.0697138069,-3.3193094903,-3.3305849916
O,0,-2.1373223694,-3.6606989616,-3.8095134924
C,0,-0.0517498544,-2.543890241,-4.1182046327
```

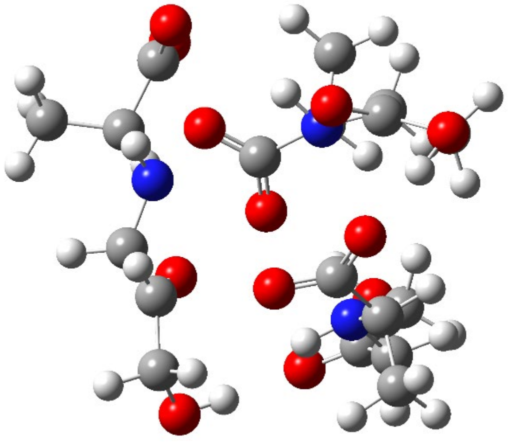

H,0,0.1741049749,-1.6358564516,-3.5383209407
H,0,0.8716249021,-3.1354950922,-4.1209493277
O,0,-0.4075208856,-2.2604709079,-5.4413566106
H,0,-1.1142069809,-1.5760520234,-5.450647103
N,0,-1.663472995,1.0118191551,-2.7297495977
H,0,-2.3805884998,1.6839477721,-2.478517028
C,0,-1.3672509172,1.1523939957,-4.1584630613
H,0,-0.5238081188,0.4913903747,-4.370313572
C,0,-1.0056538144,2.5649637553,-4.6059126659
H,0,-0.0945587699,2.9147428693,-4.1138739878
H,0,-0.8279683008,2.5945727301,-5.685484863
H,0,-1.8131032436,3.2652143862,-4.3732662341
C,0,-2.573590789,0.625790769,-4.9495263925
O,0,-2.3993742833,-0.4013927786,-5.6671554298
O,0,-3.6585853935,1.2436303869,-4.8396534006
C,0,-0.5095852187,1.2104106367,-1.8803157923
H,0,-0.0677920587,2.2212715761,-1.9360801576
H,0,0.2847458768,0.5207403822,-2.1806469747
C,0,-0.8086264947,1.006363287,-0.4201765544
O,0,-1.9046081091,1.2174812207,0.0642725463
C,0,0.3596570425,0.5330686514,0.4112979251
H,0,0.6707183661,-0.4375699627,-0.0005528261
H,0,1.197205643,1.2191321803,0.2461055319
O,0,0.1052354666,0.4590844358,1.7881660014
H,0,-0.5334040974,-0.2682387987,1.9410127856
N,0,-5.3221428462,0.0418553893,-1.4014559055
H,0,-5.2924892771,0.4214506985,-2.3410874455
C,0,-5.9771900568,1.0051865016,-0.5223809595
H,0,-5.9251294246,0.6049923708,0.4938411494
C,0,-7.4427351368,1.2975367951,-0.852694073
H,0,-8.0612236372,0.4017182612,-0.7578208529
H,0,-7.8495714674,2.0482707444,-0.1691295463
H,0,-7.5420460637,1.6749728141,-1.8754979275
C,0,-5.2015439104,2.3318973818,-0.533940535
O,0,-5.0893274601,2.9381110008,0.5652652753
O,0,-4.7696773039,2.7490119098,-1.6386804566
C,0,-5.9179222144,-1.2714924199,-1.4279540548
H,0,-6.9805177842,-1.2925653856,-1.7314574256
H,0,-5.8843477882,-1.7186595992,-0.4302222843
C,0,-5.2351004345,-2.1827158216,-2.4087211855
O,0,-4.6532378885,-1.762579456,-3.391231812
C,0,-5.3257598396,-3.6587742709,-2.1117908862
H,0,-4.8229885198,-3.8227337245,-1.1481084085
H,0,-6.3770922803,-3.9230668872,-1.9626965527
O,0,-4.8023871878,-4.4831282867,-3.1221419767
H,0,-3.8777481936,-4.2295307708,-3.276269358

## {F6} ASC -

Charge = -1 Multiplicity = 1
C,0,-2.0113725502,0.2411115215,0.5585246119
O,0,-2.6529667905,1.1933390879,-0.1957674898
C,0,-1.6691612519,2.0820058343,-0.7429547457
C,0,-0.3252085275,1.5548095183,-0.2661404315
C,0,-0.6184052391,0.456016813,0.5170648657
O,0,0.3076571947,-0.3212725445,1.1803235569
H,0,-0.1497244448,-1.0728175586,1.5727606118
O,0,0.7715858386,2.0942927076,-0.5669270273
O,0,-2.667526667,-0.6237909288,1.1398780469
H,0,-1.8391872506,3.0733156671,-0.311370911
C,0,-1.7957909299,2.188120694,-2.2495130571
H,0,-1.0087030282,2.8839412785,-2.5719356212
C,0,-3.1326084813,2.7580428528,-2.6731837661
H,0,-3.9429244382,2.083637927,-2.3792120803
H,0,-3.2843869098,3.7267472992,-2.1831808379
O,0,-3.1107210044,2.9108909838,-4.0868750808

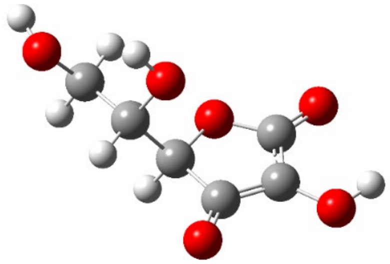

H,0,-4.0142124402,3.0158908515,-4.3935843144
O,0,-1.5694960487,0.9121976054,-2.826763254
H,0,-1.7310730409,0.99214091,-3.7716127662

## {F7} $[(ASC)_2]^{2-}$

Charge = -2 Multiplicity = 1
C,0,-4.1607293986,2.9753790397,-1.5552590561
O,0,-4.5248012968,4.2980393463,-1.4610664638
C,0,-3.3397161878,5.1018508017,-1.4204238229
C,0,-2.1854644747,4.1299389005,-1.5622449281
C,0,-2.7474614772,2.869678514,-1.6160371899
O,0,-2.1113678617,1.6615852484,-1.7585479149
H,0,-1.4736039718,1.5475287219,-1.013494027
O,0,-0.9810732585,4.5120626356,-1.5901246218
O,0,-5.0360134433,2.1132968031,-1.5790088375
H,0,-3.2906968667,5.576361384,-0.4351506583
C,0,-3.3697217491,6.1918357912,-2.4733131594
H,0,-2.4293055905,6.7487797962,-2.3612419163
C,0,-4.518131823,7.1554915688,-2.2635326283
H,0,-5.4746970718,6.6386018586,-2.3876396338
H,0,-4.4666563912,7.5623587584,-1.2469751692
O,0,-4.395017934,8.1965508109,-3.2245265126
H,0,-5.2420052242,8.6423202576,-3.2972152659
O,0,-3.4186867732,5.5941918223,-3.7586992333
H,0,-3.5113093374,6.3022878312,-4.4031122532
C,0,2.6832495753,2.8011674249,0.2914991498
O,0,2.7009528707,2.2589007622,1.5549081662
C,0,1.4427918505,1.6195291357,1.801160783
C,0,0.6525165809,1.7716247201,0.5169053939
C,0,1.4464081933,2.5094086484,-0.3388679222
O,0,1.1757021926,2.8943369666,-1.6283956401
H,0,0.3486703113,3.4336084304,-1.6326853303
O,0,-0.5094117216,1.2937881297,0.3789509953
O,0,3.6665691547,3.4198238179,-0.1088403513
H,0,0.9381316094,2.1707478729,2.6010189303
C,0,1.6265088474,0.1845869497,2.2536798965
H,0,0.6159303478,-0.2145454441,2.4166212857
C,0,2.3880127362,0.0915177628,3.5586564311
H,0,3.4092597868,0.4628914202,3.4293637092
H,0,1.8835176538,0.7036339789,4.3152211346
O,0,2.4093150203,-1.2741403797,3.9548689446
H,0,3.1106372345,-1.3907047415,4.5998029492
O,0,2.2673592049,-0.5487129402,1.2225171947
H,0,2.4367150632,-1.4335022357,1.559714772

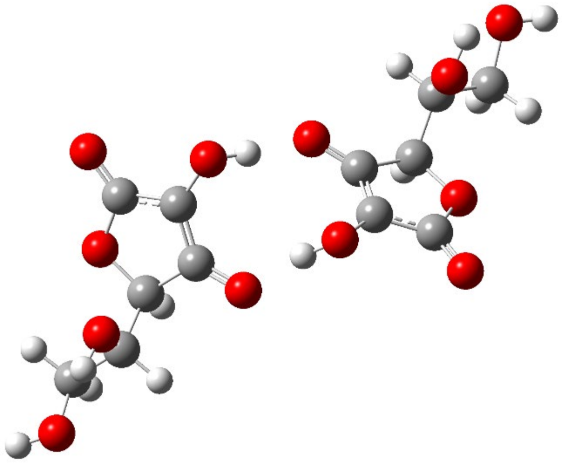

## {F8} $[(ASC)_3]^{3-}$

Charge = -3 Multiplicity = 1
C,0,-1.4003157186,-0.9014145138,0.7522938361
O,0,-2.4458775047,-1.206765207,-0.0907595056
C,0,-2.5602828651,-0.1724077444,-1.0743038298
C,0,-1.4260235768,0.7981820598,-0.787326046
C,0,-0.7722987725,0.2921783695,0.3238423104
O,0,0.269957143,0.8911075688,0.9912548774
H,0,1.0124039562,0.259946275,1.0569070448
O,0,-1.2296409044,1.8360821425,-1.4684735516
O,0,-1.1554888123,-1.6494732115,1.6979234344
H,0,-3.5150807311,0.3389641776,-0.91432945
C,0,-2.560397843,-0.7375771175,-2.48017058
H,0,-2.6231625162,0.1231533539,-3.1592372797
C,0,-3.749716579,-1.6374040717,-2.7360667256
H,0,-3.7237845444,-2.5022201889,-2.0660977872
H,0,-4.6715956456,-1.0744628365,-2.5516582864
O,0,-3.6842799873,-2.0644986695,-4.0906365997
H,0,-4.234174147,-2.8451497804,-4.1897950441

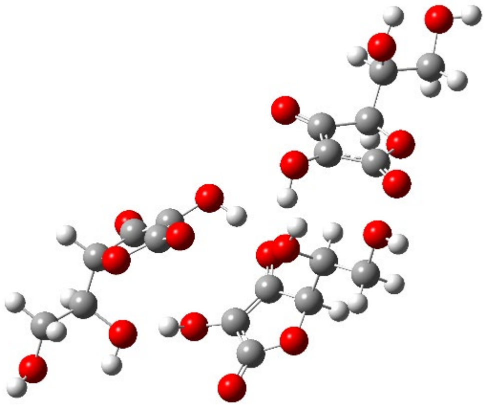

O,0,-1.3409935868,-1.4335881331,-2.7121354266
H,0,-1.4447351136,-1.9264894595,-3.533928039
C,0,2.2739012698,-2.0773280689,-2.0055053019
O,0,3.2463174119,-2.2496549273,-1.0485198028
C,0,3.6889557431,-0.9614858758,-0.6053640496
C,0,2.8362915678,0.044854264,-1.3543240149
C,0,2.014686492,-0.6937924784,-2.1817157807
O,0,1.1306271481,-0.1936699079,-3.1076249094
H,0,0.2446711773,-0.5689621497,-2.939035105
O,0,2.9565409254,1.2915500961,-1.2008923873
O,0,1.7792897521,-3.0643847605,-2.5448404671
H,0,4.7327382033,-0.8409924614,-0.9132197738
C,0,3.6251323606,-0.8303953057,0.9022273261
H,0,3.9412501129,0.195724892,1.1289969262
C,0,4.5609357243,-1.7899783939,1.6041090421
H,0,4.2835660321,-2.8256932806,1.3830177629
H,0,5.5832937201,-1.6148124135,1.2503848327
O,0,4.462589706,-1.5435680137,3.0005937927
H,0,4.8512299037,-2.2875003467,3.4664738753
O,0,2.281833191,-1.018376363,1.3356085174
H,0,2.2957274454,-1.0855902011,2.2968460974
C,0,5.4904100044,3.1370393547,1.3843929163
O,0,6.3965867395,4.1715790504,1.2964933325
C,0,5.7093808824,5.3555450767,0.8735068081
C,0,4.2554428188,4.9474084284,0.7068736867
C,0,4.2039960538,3.5949148363,1.0083725077
O,0,3.0729843207,2.8135437151,1.0512178634
H,0,3.058948402,2.2149645501,0.2722785683
O,0,3.3580411402,5.753857452,0.3561066004
O,0,5.8867357976,2.0298441936,1.7451823537
H,0,6.1130408121,5.6481644551,-0.1008066496
C,0,5.9254303209,6.4998089486,1.8445113015
H,0,5.3664306486,7.3540280242,1.4379400998
C,0,7.3826367431,6.8949453499,1.9492510998
H,0,7.9689441951,6.0719203499,2.3695376163
H,0,7.7665911372,7.1271778313,0.9492244276
O,0,7.4646495812,8.0368689694,2.7938191462
H,0,8.3774905328,8.1421359965,3.0719390652
O,0,5.3923349034,6.1402413176,3.1093988924
H,0,5.6119278287,6.8446417831,3.7262724316

**{F9} PM $^{-}$**

Charge = -1 Multiplicity = 1
C,0,-1.5170950161,-1.081297194,-0.0995240285
C,0,-0.1292448938,-1.0233362342,-0.0261852378
C,0,0.4797970623,0.2156735038,0.2123923305
C,0,-0.3348434551,1.3756604687,0.3599477177
C,0,-1.7531516773,1.1743752522,0.2689319058
N,0,-2.3108118322,-0.0133241977,0.0433072306
H,0,-2.0108204623,-2.0329210359,-0.2865057835
C,0,-2.6537452706,2.3584380247,0.4295311384
H,0,-2.5083376885,2.8427732354,1.4015509009
H,0,-2.4475274972,3.1238918448,-0.3269197045
H,0,-3.6994780385,2.0597529706,0.3443876395
C,0,0.6507277057,-2.2945674447,-0.1890712
H,0,0.019295148,-3.0509141935,-0.6668872393
H,0,1.5264315726,-2.1401125795,-0.828467956
O,0,1.0725186834,-2.7683585577,1.0960092257
H,0,1.5734956262,-3.5770060383,0.9576341874
C,0,1.9689854196,0.3860557385,0.3068059055
H,0,2.1915191874,0.9489540057,1.2240547618
H,0,2.4643689534,-0.5789095607,0.4084151343
N,0,2.5193229227,1.050628181,-0.8848763864
H,0,2.1115563045,1.9782268214,-0.9316425512
H,0,3.5091864928,1.2054808468,-0.726030841

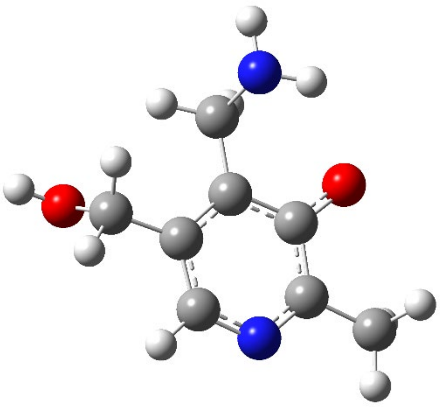

O,0,0.1598134431,2.5532839623,0.5659400901

## {F10} $[(PM)_2]^{2-}$

Charge = -2 Multiplicity = 1
C,0,2.8333239269,4.5034927349,6.8113145468
C,0,2.7477420786,4.7679404915,5.4483456383
C,0,1.9605313262,5.8447775054,5.0197244483
C,0,1.2672350077,6.6290725369,5.9876751673
C,0,1.4454645964,6.250217833,7.3611423756
N,0,2.1994738871,5.2221381959,7.7453870366
H,0,3.4357366811,3.6689618025,7.1648890402
C,0,0.7489777494,7.0493647512,8.4175587837
H,0,1.0643374884,8.0986442437,8.3966120812
H,0,-0.3359812347,7.0532293208,8.2651662883
H,0,0.9576400812,6.6452594916,9.4088992418
C,0,3.5225441231,3.8995515041,4.5011341696
H,0,3.773654624,2.9547076881,4.9949627468
H,0,2.9331982289,3.6620452472,3.6096731828
O,0,4.7287617254,4.5703864297,4.1145869129
H,0,5.1844986272,4.0085397685,3.4817343999
C,0,1.7980586658,6.2056620146,3.5704200465
H,0,1.91821954,7.2942209656,3.4745362479
H,0,2.5895251736,5.7558092718,2.9708913056
N,0,0.5073512399,5.7531464938,3.0283271835
H,0,-0.2225219779,6.2074728326,3.5681149772
H,0,0.4160179246,6.1465199162,2.0928279809
O,0,0.51715341,7.6324115296,5.6654172515
C,0,-0.6208197464,10.23322528,-2.114426329
C,0,-0.1453167365,10.4821678738,-0.8315733347
C,0,0.2213412832,9.394832815,-0.0270323749
C,0,0.0912638878,8.0718571453,-0.5427125022
C,0,-0.4044358973,7.9571349999,-1.8856480502
N,0,-0.7495676615,9.0049541917,-2.6304729011
H,0,-0.9152934969,11.0628727368,-2.7541664549
C,0,-0.5463233526,6.5914071785,-2.4803501249
H,0,0.4107477622,6.0581369895,-2.4887147357
H,0,-1.2361996335,5.9709359281,-1.8974121263
H,0,-0.9180313379,6.6538248495,-3.5039823307
C,0,-0.0223223868,11.9076059717,-0.3792448469
H,0,-0.6582358556,12.544415967,-1.0033685868
H,0,-0.3514646207,12.024892753,0.6581463146
O,0,1.3417037882,12.3306313038,-0.501255603
H,0,1.393773495,13.23947636,-0.1929127069
C,0,0.7297376006,9.558734741,1.3768624079
H,0,1.5921984773,8.891379516,1.5083034842
H,0,1.0923154466,10.573272691,1.541803004
N,0,-0.3206129046,9.2861903938,2.3723607103
H,0,-0.636505081,8.3331052142,2.2277666654
H,0,0.1177837363,9.2659510287,3.2881984078
O,0,0.3995483412,7.0206205025,0.1444609608

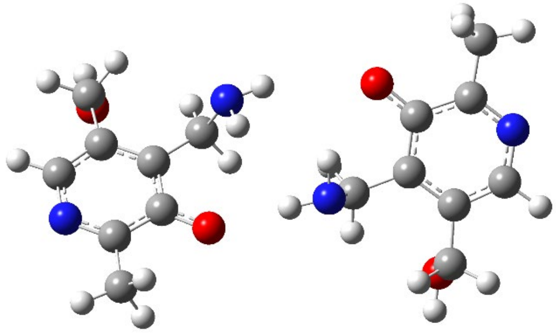

**{F11} [(PM)$_3$]$^{3-}$**

Charge = -3 Multiplicity = 1
C,0,3.7073433003,4.7200916718,7.2620142857
C,0,3.7533042808,5.0356527747,5.908281249
C,0,2.8480075988,5.9790317509,5.4039469307
C,0,1.9176031039,6.5948545553,6.2932242772
C,0,1.9682713644,6.166066794,7.6637954131
N,0,2.8325952403,5.2615800637,8.1188817215
H,0,4.4001095256,3.9878578403,7.6723831933
C,0,0.9901753947,6.7582750708,8.629604209
H,0,1.0872005265,7.848486498,8.680085453
H,0,-0.043516645,6.5574817131,8.324079984
H,0,1.1405690767,6.3505082025,9.6302076385
C,0,4.7856560468,4.3625878742,5.0513550311
H,0,5.1438568168,3.457842594,5.5540942254
H,0,4.3644767172,4.062745192,4.0870724373
O,0,5.8862456299,5.2560876892,4.8349970531
H,0,6.4869509311,4.8343541607,4.2142253861
C,0,2.7821544319,6.3363693048,3.9453314054
H,0,2.5981944523,7.4164644032,3.86292645
H,0,3.7405091003,6.1475832879,3.4596973729
N,0,1.7536382448,5.5521797343,3.2466126807
H,0,0.858313209,5.716531792,3.6974341568
H,0,1.6279977874,5.9378293629,2.3134045304
O,0,1.0641653921,7.4848100275,5.9094891972
C,0,-1.8732542857,8.9680020618,-1.4993883844
C,0,-1.1674326465,9.4088799251,-0.3858727153
C,0,-0.2929593629,8.5207762066,0.2556358815
C,0,-0.1518158308,7.1940276971,-0.2505966953
C,0,-0.9257133793,6.8761162725,-1.4205609514
N,0,-1.7576983455,7.7343403715,-2.0063453653
H,0,-2.5645928387,9.6396776872,-2.0048181841
C,0,-0.800372627,5.5067505521,-2.0115447993
H,0,0.2392359064,5.2776417581,-2.2705795215
H,0,-1.1163619906,4.7338057809,-1.301489435
H,0,-1.4097795477,5.4195848926,-2.9119624775
C,0,-1.348731984,10.8315577726,0.0569297344
H,0,-2.3126822984,11.2072229263,-0.3027748816
H,0,-1.3451817976,10.9132387454,1.1475241308
O,0,-0.2921966837,11.6351649944,-0.4868558602
H,0,-0.4023903186,12.5285371227,-0.1496836917
C,0,0.472135772,8.9058677552,1.4906995446
H,0,1.4395852959,8.3883387845,1.4735310136
H,0,0.6925088561,9.9749668734,1.4836102454
N,0,-0.2863232859,8.6051219559,2.7154587998
H,0,-0.5407553567,7.6205547391,2.7121045468
H,0,0.3436689266,8.7014227638,3.5058556501
O,0,0.6238992666,6.3142351258,0.2912923653
C,0,-5.0279308461,3.9495369072,4.1643262647
C,0,-4.1756864946,4.1946556276,5.2360532656
C,0,-2.9420587298,4.8154153304,4.9895881518
C,0,-2.596398921,5.1675792795,3.6504918835
C,0,-3.5743940505,4.8682815701,2.6400368953
N,0,-4.7399763985,4.2809428556,2.899065929
H,0,-5.9899823939,3.4701700354,4.334476864
C,0,-3.2595748054,5.22790163,1.2215335463
H,0,-3.0649920092,6.3013054605,1.1141727644
H,0,-2.3523587576,4.7190133088,0.8763034121
H,0,-4.0845392155,4.9568464252,0.5609465447
C,0,-4.6160522442,3.7823140495,6.610082216
H,0,-4.4936312992,4.6051183046,7.3232597557
H,0,-5.6784786616,3.5178305989,6.5877723057
O,0,-3.8506255365,2.6540639519,7.052429644
H,0,-4.1227598855,2.4527239301,7.9519930918
C,0,-1.9640456297,5.1179784625,6.0891098852

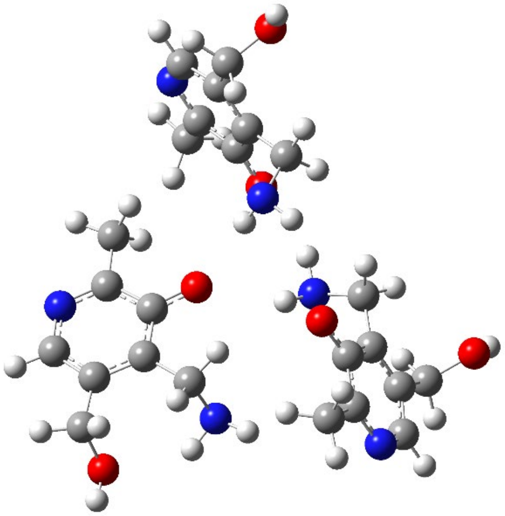

```
H,0,-1.3688998833,5.9821465326,5.7910351211
H,0,-2.5056276396,5.4023936603,7.0003112684
N,0,-1.0302616044,4.005501912,6.3319572487
H,0,-0.3532953302,4.3104435925,7.0238379802
H,0,-1.5545245607,3.254465156,6.7681427077
O,0,-1.4789143638,5.7304112763,3.339778143
```

## {F12} $H_2O$

```
Charge =  0 Multiplicity = 1
O,0,-0.0087407232,0.7147435702,0.
H,0,0.9505415872,0.7604585101,0.
H,0,-0.2858629338,1.6342628398,0.
```

## {F13} $(H_2O)_2$

```
Charge =  0 Multiplicity = 1
O,0,1.486483,-0.080939,-0.054554
H,0,0.515213,-0.030831,-0.035844
H,0,1.77307,0.654038,0.492185
O,0,-1.341083,-0.040904,-0.080411
H,0,-1.715545,-0.484039,0.686209
H,0,-1.735935,0.835568,-0.062829
```

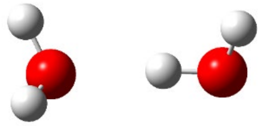

## {F14} $(H_2O)_3$

```
Charge =  0 Multiplicity = 1
O,0,-0.0557751982,-0.2216755326,0.3320773167
H,0,0.5165533099,-0.2477740092,1.1048430438
H,0,0.5230923213,-0.4146886404,-0.4114758407
O,0,-1.9845179186,1.8368964455,-0.0646376145
H,0,-1.2484072258,1.2175441393,0.064283187
H,0,-1.5696097005,2.6475905394,-0.368041244
O,0,-2.6210439805,-1.46510096,0.2197393633
H,0,-1.7244267025,-1.1002278895,0.2892089428
H,0,-3.164398765,-0.6982306224,0.0218053656
```

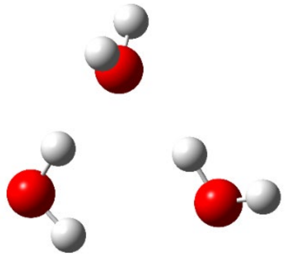

## {F15} $(H_2O)_4$

```
Charge =  0 Multiplicity = 1
O,0,0.0000005651,-1.849211,0.000000025
H,0,0.764585,-1.25391,0.02216
H,0,-0.764588,-1.253915,-0.02216
O,0,2.197269,0.0000008464,0.007559
H,0,2.856985,0.000009,0.707698
H,0,2.706061,0.0000010019,-0.808791
O,0,-2.197269,-0.0000004964,-0.007559
H,0,-2.706061,0.000003,0.808791
H,0,-2.856985,0.000013,-0.707698
O,0,-0.0000005651,1.84921,0.000000025
H,0,0.764588,1.253913,0.022156
H,0,-0.764585,1.253909,-0.022155
```

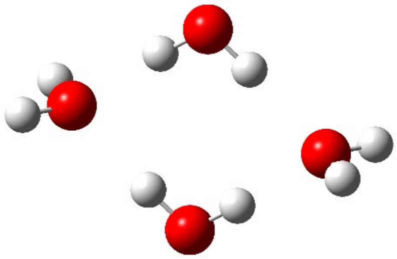

## {F16} $(H_2O)_5$

```
Charge =  0 Multiplicity = 1
O,0,-0.0805718491,0.0620383039,-0.2878768372
H,0,0.0227003615,0.9056633419,-0.758886545
H,0,0.4271712779,-0.5711259382,-0.8227542101
O,0,1.4514715875,-1.7520145905,-1.8619313505
H,0,1.2034992538,-2.6678065191,-1.7116232604
H,0,1.2522948503,-1.5931993261,-2.800543363
O,0,0.351230431,2.4929523733,-1.7268570389
H,0,0.9831624949,2.1194633851,-2.3710577794
H,0,-0.3978351535,2.7753313181,-2.2575746477
O,0,2.2054679714,1.2987131945,-3.4523757716
H,0,1.7915675456,0.5173951826,-3.8583563766
H,0,2.8825986264,0.9349559878,-2.8751091313
O,0,0.9644598882,-1.0059727443,-4.5415267481
H,0,0.047027195,-0.9195518091,-4.8178750731
H,0,1.4025194693,-1.4584713497,-5.268686847
```

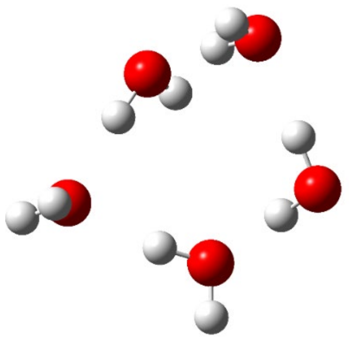

## {F17} $(H_2O)_6$

```
Charge =  0 Multiplicity = 1
O,0,-1.830824,0.773101,-1.412999
H,0,-1.176498,1.292049,-0.908105
H,0,-1.542773,0.835354,-2.327272
O,0,0.051318,2.016955,0.241006
H,0,0.928459,1.653048,0.003213
H,0,0.168187,2.968655,0.298612
O,0,-1.333932,-1.628604,-0.110803
H,0,-1.975857,-2.318895,-0.29517
H,0,-1.588694,-0.868113,-0.670306
O,0,1.44135,-1.913936,-0.168545
H,0,1.691951,-2.47521,-0.906814
H,0,0.46638,-1.887638,-0.195319
O,0,2.384638,0.694913,-0.451456
H,0,3.091011,0.800867,0.190927
O,0,-0.78835,0.062619,2.158839
H,0,-1.035289,-0.615315,1.512328
H,0,2.095485,-0.233949,-0.352374
H,0,-0.515957,0.808757,1.601936
```

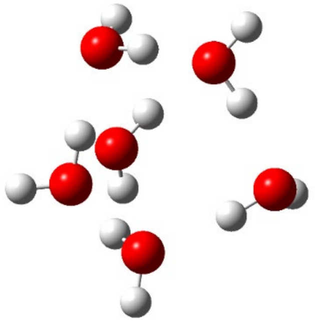

## {G2} $[Cu(AG_D)_2]^{2+}$ (mirror image)

```
Charge = 2 Multiplicity = 2
Cu,0,0.0232515895,-0.1534066206,-0.0253631415
N,0,-1.2646397875,-1.753649724,0.3771330313
H,0,-1.3051714619,-2.3702299217,-0.4321235206
H,0,-0.9455265299,-2.2991820052,1.1723073126
N,0,-1.7039054032,0.7796407023,-0.0335549664
H,0,-1.8989608487,1.7541886342,-0.2170805817
C,0,-2.7399732126,0.0682227754,0.3074431099
N,0,-2.5325678536,-1.2358631589,0.6568161039
H,0,-3.3074753818,-1.8821233489,0.588756756
N,0,-4.0125509224,0.5065524827,0.3404348152
H,0,-4.1512458152,1.5047956375,0.3505210083
H,0,-4.6960324408,-0.0397299361,0.8418760845
N,0,1.8290354104,-1.2101230176,-0.0812664774
H,0,1.9446304472,-1.813694698,0.7274102673
H,0,1.8405277401,-1.7950860153,-0.9145943395
N,0,2.8822710437,-0.2906526846,-0.1208587033
C,0,2.5375912562,1.004097709,-0.3918136495
N,0,1.2770827847,1.3246043849,-0.3307180504
N,0,3.5430485177,1.8437561897,-0.6992201036
H,0,3.3518148496,2.8315235291,-0.641537546
```

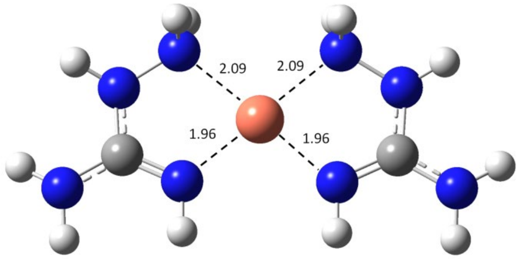

H,0,4.4890201269,1.559952931,-0.4961830706
H,0,1.0783784425,2.3015435201,-0.4965173032
H,0,3.7614054592,-0.635082365,-0.4838610153

## {G5} $[Fe(AG_D)_3]^{3+}$ (same orientation)

Charge = 3 Multiplicity = 6
Fe,0,-0.0662117452,-0.0289469165,-0.1879240991
N,0,-0.2705580486,1.7282483338,1.2686198592
H,0,-1.2321188227,2.0596710569,1.209995999
H,0,-0.0866220121,1.4798444446,2.2375111961
N,0,0.6095616658,2.735527059,0.8802425381
H,0,0.4865892085,3.6556427984,1.281914151
C,0,1.1851742141,2.6045476462,-0.3405633682
N,0,1.8932947361,3.6338277742,-0.8075034643
H,0,2.0730517013,4.4419184368,-0.2331329095
H,0,2.4249535862,3.5199425072,-1.6547970123
N,0,1.0174128605,1.4656042225,-0.9775814394
H,0,1.530414815,1.4071079087,-1.8486011421
N,0,1.60245049,-0.6356427651,1.2649196556
H,0,1.2667449043,-0.9002704588,2.1892257114
H,0,2.2279664702,0.1583737031,1.3802552463
N,0,2.3073578022,-1.6920832625,0.6974919149
C,0,1.7909060996,-2.2649399993,-0.4134106452
H,0,2.9683525547,-2.1950987197,1.2736536988
N,0,2.360332098,-3.3924080939,-0.8472985659
H,0,3.2210757498,-3.717551082,-0.4351588637
H,0,2.1134794417,-3.7410245941,-1.7591639134
N,0,0.7641193195,-1.6770519598,-0.9913540512
H,0,0.4387765579,-2.1579228332,-1.8209778619
N,0,-1.4007606034,-1.0852580019,1.3325668637
H,0,-1.2774420616,-0.7535023515,2.2862469748
H,0,-1.1528510213,-2.0732789966,1.3126691255
N,0,-2.730449789,-0.916281369,0.9598490195
H,0,-3.427646699,-1.5079614045,1.3916860576
C,0,-2.9649479489,-0.3637667406,-0.254357875
N,0,-4.2253965115,-0.3463629087,-0.6902907206
H,0,-4.9811155135,-0.6480689402,-0.0958668144
H,0,-4.4490846282,0.1515898433,-1.5359789292
N,0,-1.9366726842,0.1246648043,-0.915342449
H,0,-2.1902118563,0.5801942987,-1.7835031971

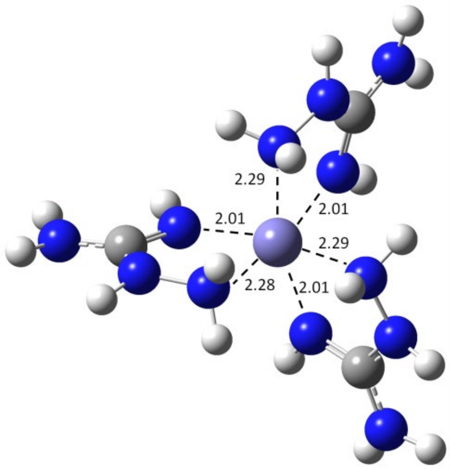

Supplement: Supplementary file 1 [file antioxidants-10-00208-s001.pdf]
